# Rapid learning of neural circuitry from holographic ensemble stimulation enabled by model-based compressed sensing

**DOI:** 10.1038/s41593-025-02053-7

**Published:** 2025-09-17

**Authors:** Marcus A. Triplett, Marta Gajowa, Benjamin Antin, Masato Sadahiro, Hillel Adesnik, Liam Paninski

**Affiliations:** 1 Mortimer B. Zuckerman Mind Brain Behavior Institute, Columbia University, New York, NY USA; 2 Grossman Center for the Statistics of Mind, Columbia University, New York, NY USA; 3 Center for Theoretical Neuroscience, Columbia University, New York, NY USA; 4 Department of Statistics, Columbia University, New York, NY USA; 5 Department of Molecular and Cell Biology, University of California, Berkeley, CA USA

**Keywords:** Neural circuits, Computational neuroscience

## Abstract

Discovering how computations are implemented in the brain at the level of monosynaptic connectivity requires probing for connections from potentially thousands of presynaptic candidate neurons. Two-photon optogenetics is a promising technology for mapping such connectivity via sequential stimulation of individual neurons while recording postsynaptic responses intracellularly. However, this technique is currently not scalable because stimulating neurons one by one requires prohibitively long experiments. Here we developed novel computational tools that, when combined, enable learning of monosynaptic connectivity from high-speed holographic ensemble stimulation. First, we developed a model-based compressed sensing algorithm that identifies connections from postsynaptic responses evoked by stimulating many neurons at once, greatly increasing mapping efficiency. Second, we developed a deep-learning method that isolates the postsynaptic response to each stimulus, allowing stimulation to rapidly switch between ensembles without waiting for the postsynaptic response to return to baseline. Together, our system increases the throughput of connectivity mapping by an order of magnitude, facilitating discovery of the circuitry underlying neural computations.

## Abstract (web summary)

The authors develop a new computational system for high-throughput mapping of synaptic connectivity using two-photon holographic optogenetics and intracellular recordings.

## Main

The structure of synaptic connectivity is central to how the brain implements neural computations. To uncover such connectivity, two-photon optogenetic stimulation has emerged as a promising technology due to its ability to flexibly probe neurons with single-cell resolution while monitoring postsynaptic currents (PSCs) using whole-cell recordings^1–6^. Yet existing optogenetic circuit mapping techniques have been limited to probing connectivity from small numbers of neurons that must be slowly stimulated one by one, and therefore require aggregating small-scale maps across experiments to obtain large-scale maps of connectivity^5^. By contrast, an ideal monosynaptic connectivity mapping technique would enable large numbers of synaptic connections to be identified at high speed within a single experimental session. This would provide a crucial advantage in that each experiment would produce a more comprehensive and representative map of neural circuitry, rather than having to pool together smaller experiments that each provide just a partial view of connectivity.

Multiple strategies could serve to advance optogenetic circuit mapping toward this state, although each introduces experimental and computational challenges. The simplest strategy to improve the throughput of a connectivity mapping experiment is to increase the rate at which stimulation switches between neurons. This is primarily determined by the refresh rate of the holographic spatial light modulator (SLM) if using advanced light sculpting techniques, or, in the case of laser-scanning approaches, just the time required for a neuron to integrate photocurrent until it elicits an action potential^7^. However, while this approach allows a mapping experiment to be completed in a shorter time frame, naively stimulating too quickly confounds postsynaptic measurements because the membrane conductance of the postsynaptic neuron will not have sufficient time to return to baseline conditions before the next stimulus is applied. The speed at which a mapping experiment can feasibly take place therefore becomes limited by the ability to computationally demix PSC waveforms that overlap in time.

Simulation studies predict that connectivity mapping could also be greatly accelerated through the use of compressed sensing, where, in principle, sparse connectivity could be reconstructed from few measurements provided that stimulation is applied to ensembles of randomly selected neurons at once^8–13^. However, the efficacy of existing compressed sensing algorithms is fundamentally limited outside of simplified simulations because they neglect the complex biophysics intrinsic to mapping experiments. For example, failing to elicit spikes in presynaptic neurons (due to inadequate laser power or physiological stochasticity^14^), as well as neurotransmitter failing to be released following successful presynaptic spikes^15^, are both known to significantly impact the accuracy of compressed sensing^8,13^. Further, high rates of spontaneous synaptic currents could lead to both false-positive connections and mischaracterized synaptic weights^6,11^.

To overcome these limitations and enable high-speed, large-scale connectivity mapping experiments, we developed new computational tools for inferring connectivity from holographic stimulation of neural ensembles. While existing optogenetic circuit mapping techniques perform slow single-neuron stimulation, our tools allow experiments to proceed with rapid holographic ensemble stimulation, minimizing downtime of the holographic SLM as evoked PSCs are isolated in time, deconfounded of spontaneous PSCs and cleaned of electrical noise by a computational demixing procedure. To infer connectivity, we developed a model-based compressed sensing algorithm that simultaneously estimates synaptic weights, presynaptic spikes, and how presynaptic spikes depend on laser power, all while accounting for critical biophysical constraints. To validate these tools, we performed connectivity mapping experiments using the recently engineered family of fast, potent ChroME2.0 opsins^16^ together with two-photon holography^17^ and intracellular recordings. We routinely mapped connectivity from hundreds of presynaptic candidates within 680 × 680 × 100 μm^3^ volumes of cortical slices, together totaling ~12,000 probed targets across experiments. By combining rapid ensemble stimulation with computational demixing and model-based compressed sensing, we reduced the stimulation time required to reconstruct connectivity by an order of magnitude over existing approaches, allowing large numbers of synaptic connections to be quickly mapped within individual experiments.

## Results

### Optogenetic circuit mapping framework

**Fig. 1:**
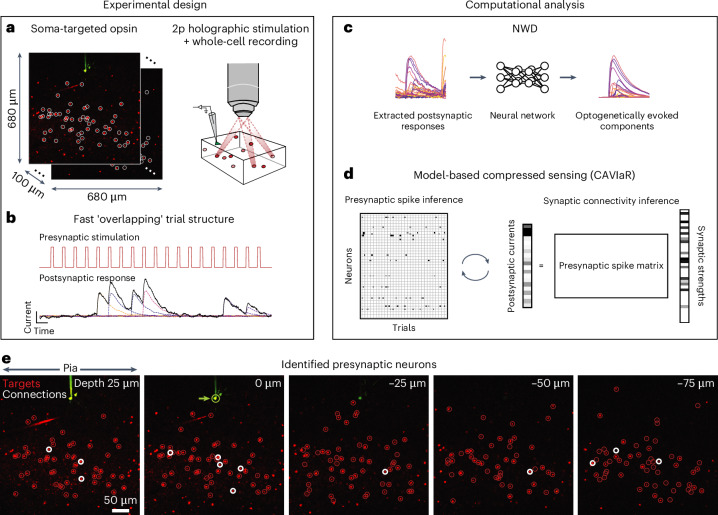
Overview of proposed high-throughput optogenetic connectivity mapping framework. **a**, A fast, potent and soma-targeted opsin (here ChroME2f) is expressed in a pool of candidate presynaptic neurons while intracellular currents are monitored from a voltage-clamped postsynaptic neuron. Two-photon (2p) holography is used to elicit spikes in ensembles of presynaptic candidates, increasing the number of putative synaptic connections tested compared to single-target stimulation. **b**, To further increase throughput, stimulation is applied at high speeds (for example, 30−50 Hz), such that the postsynaptic membrane conductance is not guaranteed to return to baseline conditions (that is, trials are ‘overlapping’ in time). This allows more synapses to be tested per minute, but complicates downstream identification of their existence and strength unless the evoked PSCs are subsequently demixed. **c**, A neural network is trained to isolate the components of the postsynaptic response that are plausibly due to the optogenetic stimulus, allowing high-fidelity measurements of postsynaptic current even at very high stimulation frequencies. **d**, The ‘demixed’ postsynaptic responses evoked by holographic ensemble stimulation are used in a model-based compressed sensing algorithm (CAVIaR) to reconstruct the underlying connectivity. Unlike traditional compressed sensing techniques, compressive mapping of synaptic circuitry requires inference of any optogenetically elicited presynaptic spikes (left) as well as the synapses themselves (right). CAVIaR alternates between inference of presynaptic spikes and synaptic connectivity, in addition to a number of other critical biophysical variables (see main text). **e**, Presynaptic neurons (white circles) identified via the proposed pipeline in an example experiment mapping connectivity from presynaptic PV neurons to a postsynaptic pyramidal neuron (green circle, marked by green arrow). Probed targets considered unconnected shown as red circles.

**Extended Data Fig. 1:**
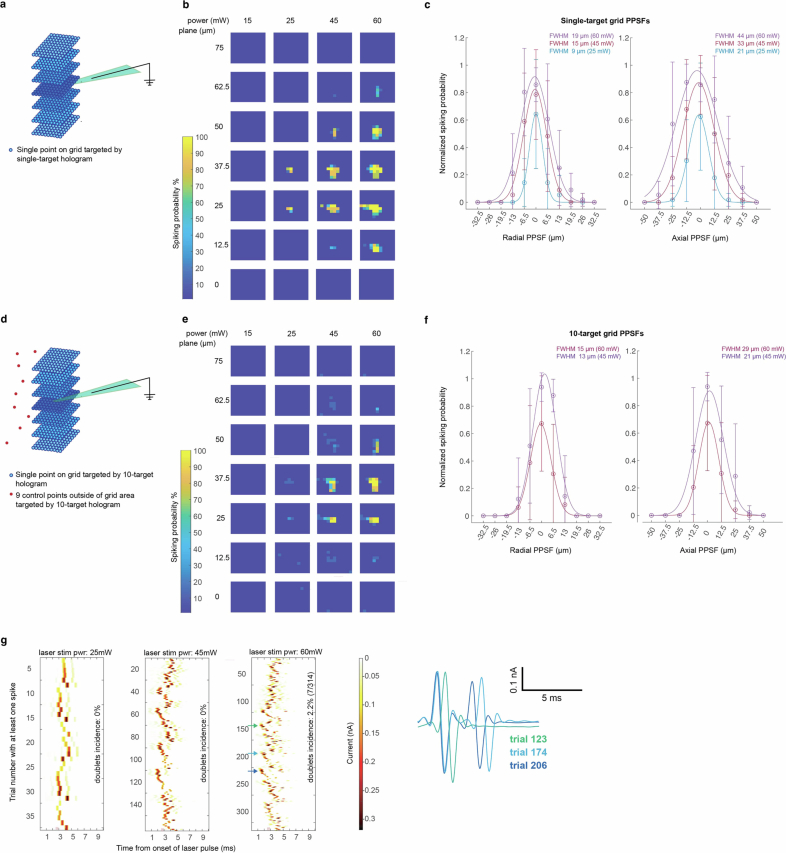
Physiological point spread functions (PPSFs) for holographic single-target and ensemble stimulation. **a**, Illustration of multi-plane grid of stimulation sites used to obtain single-target PPSFs. A loose-patched, opsin-positive cell was positioned to be in the center plane of the grid (37.5 *μ*m). Planes separated by 12.5 *μ*m. **b**, Single-cell example of mean spike probability when stimulating individual points on grid (7 repetitions per power). **c**, Mean radial (left panel) and axial (right panel) PPSF for a population of opsin-expressing PV cells. Error bars indicate standard deviation over n=8 cells. **d**, Illustration of multi-plane grid of stimulation sites used to obtain 10-target ensemble PPSFs. In these experiments, one point on the grid is targeted by the hologram while the remaining 9 “control” targets are outside of the grid area and kept fixed (that is, do not change from stimulus to stimulus). **e**, Single-cell example of mean spike probability when stimulating using a 10-target hologram (with one target being the opsin-positive cell; 7 repetitions per power). Panels (**a**) and (**c**) display results from the same cell. **f**, Mean radial (left panel) and axial (right panel) PPSF for a population of opsin-positive PV cells when stimulating 10 targets at once. Error bars indicate standard deviation over n=7 cells. **g**, Incidence of multiple action potentials (that is, doublets) in response to a single laser pulse. Trials shown are those where the patched cell spiked at least once. Postsynaptic neuron same as in panel (**b**). 10 ms time windows from the start of a 3 ms laser pulse are displayed. Example traces displayed on the right are marked by arrows.

**Extended Data Fig. 2:**
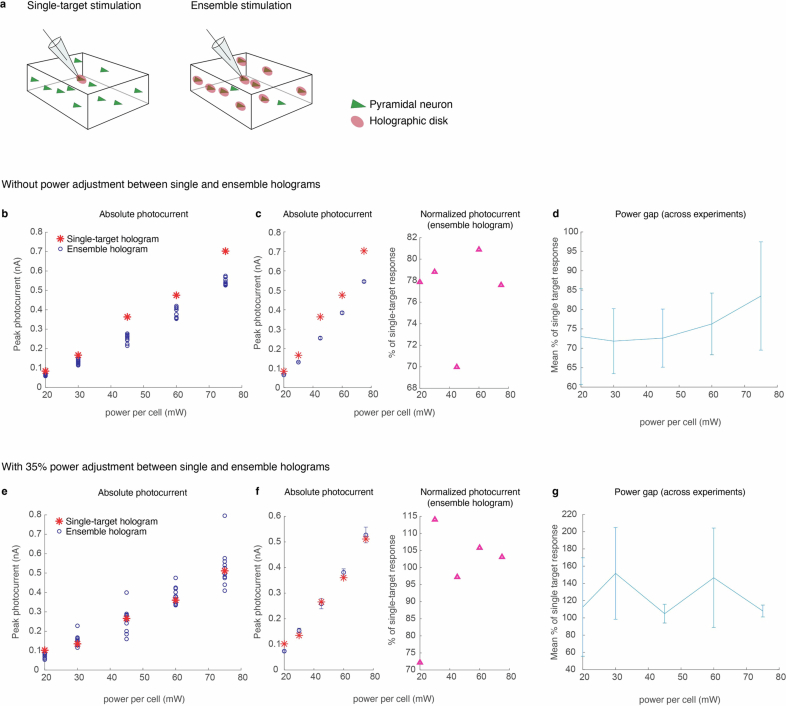
Resolving the “power gap” between single-target and ensemble holograms. **a**, Illustration of the two compared stimulation conditions. An opsin-positive cell was patched and photocurrents were recorded in whole-cell configuration while the regular connectivity mapping protocol was used. Trials when single-target holograms were targeting the patched cell (left) were compared with trials when the patched cell was a part of a 10-target ensemble hologram (right). **b**, Photocurrents from an example experiment. Red stars indicate the photocurrent amplitude across increasing stimulation power for a single-target hologram applied to the patched cell, blue circles represent the response amplitude across power for different ensemble holograms containing the patched cell. **c**, Same data as in (**b**), but showing the mean ± s.e.m. for the different ensemble hologram sets (left), and normalized to the corresponding single-target photocurrents (right, magenta triangles). **d**, Mean photocurrents evoked by ensemble holograms, normalized to photocurrents evoked by single-target holograms at the corresponding laser powers. Error bars show one standard deviation. Data collected across a population of cells; n=6 experiments performed on 1 pyramidal cell (Emx-Cre), 1 PV cell (PV-Cre), 4 SST cells (SST-Cre). **e-g**, same as (**b**)-(**d**) but with laser power for ensemble holograms increased by 35%. For (**g**), data collected across n=2 experiments (1 pyramidal cell, 1 SST cell).

Accurately characterizing the presence and strength of a synaptic connection requires precise control over the initiation of presynaptic action potentials. Ideally, each stimulus would evoke just a single presynaptic spike as this provides a direct measurement of synaptic charge transfer. To this end, we combined the family of potent soma-targeted ChroME opsins^14,16^ with the scanless computer-generated holography system 3D-SHOT^17^ (Fig. 1a). We first expressed ChroME2f in parvalbumin-expressing (PV) neurons by viral transfection with an adeno-associated virus^16^. We calibrated laser powers and illumination time such that brief (3–5 ms) periods of stimulation almost always led to either 0 or 1 action potential, with minimal instances of multiple action potentials resulting from a single pulse (Extended Data Fig. 1). Next, we confirmed through a similar process that we could reliably evoke action potentials when holographically stimulating ensembles of neurons at once (Extended Data Figs. 1 and 2). Finally, we combined holographic stimulation of ensembles of PV neurons with whole-cell recordings from pyramidal neurons. We focused our initial experiments on mapping connections of this kind for two reasons. First, measurements of evoked PSCs would not be affected by direct photocurrent artifacts caused by stimulating at locations near the recording electrode, as a postsynaptic pyramidal neuron would not be sensitive to stimulation itself. Second, PV neurons make strong synaptic connections onto pyramidal neurons^5,18^, and therefore were likely to be reliably identified computationally.

Having established the high spatiotemporal precision of our holographic stimulation technique here and in previous work^16,17,19^, we then developed a computational system for processing optogenetic data and inferring synaptic connectivity. The first component of our system seeks to extract precise measurements of optogenetically evoked PSCs. While there are many possible adverse factors that could generate variability in the membrane conductance and PSC measurements, we focused on three that we considered most critical to mapping experiments: (1) electrical noise arising from the recording electrode, which increases the variability of the observed synaptic currents and can require additional repetitions of each presynaptic stimulus to overcome; (2) spontaneous currents that, depending on their timing relative to the optogenetic stimulus, can obscure optogenetically evoked PSCs or increase the number of false-positive connections; and (3) postsynaptic responses from preceding or subsequent trials when the interstimulus interval is shorter than the PSC decay time, which are prevalent when attempting to perform mapping experiments at very high speeds. To simultaneously eliminate these factors, we developed a deep neural network architecture for demixing and denoising PSC waveforms (Fig. 1b,c) in a process that we call neural waveform demixing (NWD). The NWD network attempts to isolate optogenetically evoked PSCs, such that confounding synaptic currents not driven by the optogenetic stimulus are ‘subtracted out’ by the network and the resulting currents cleaned of electrical noise. Consequently, the NWD network allows experiments to proceed with very short interstimulus intervals as evoked PSCs from previous and subsequent trials are subtracted out and the initial baseline current reset to zero (further details given below).

The second component of our system is a model-based compressed sensing algorithm (Fig. 1d). The key challenge for a compressive approach to connectivity mapping is to robustly infer presynaptic spikes from PSCs evoked by ensemble stimulation despite a multitude of unobserved sources of biophysical variability. To do this, we embedded the compressed sensing step in a hierarchical Bayesian statistical model that captured the most critical sources of biophysical variability. We then developed a variational inference technique called CAVIaR (coordinate-ascent variational inference and isotonic regularization) to learn posterior distributions over the model parameters (Methods). CAVIaR identifies the presence and strength of individual synaptic connections from ensemble stimulation (Fig. 1e), substantially increasing the rate at which neural circuits can be mapped compared to single-target stimulation and improving mapping accuracy compared to existing compressed sensing techniques.

### Neural waveform demixing allows mapping experiments to proceed rapidly

**Fig. 2:**
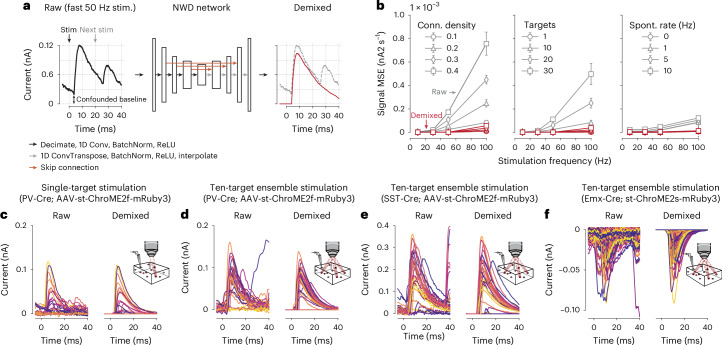
Simultaneous demixing and denoising of optogenetically evoked postsynaptic currents. **a**, NWD overview. Raw electrophysiological traces (left) are confounded by synaptic currents from previous and subsequent trials during fast stimulation, in addition to spontaneous synaptic currents. The NWD network (middle) is trained to isolate the PSC waveform associated with the optogenetic stimulus, resulting in a demixed trace (right, red) with the confounding synaptic currents subtracted and baseline current (at 0 ms) adjusted to 0 nA. Note that a 5-ms ‘pre-stimulus’ window is provided to the network as additional context. **b**, Performance of NWD in simulation as a function of stimulation frequency under increasing connection density (left), number of simultaneously targeted cells (middle) and rate of spontaneous activity (right). In all cases NWD leads to a substantial improvement in signal fidelity (measured by the mean square error of the raw versus demixed trace compared to the ground-truth evoked PSC). Errors are evaluated across all conditions using a single trained network. Number of simultaneously stimulated targets in left and right panels, 10. Connection density in middle and right panels, 0.1. Spontaneous PSC rate in left and middle panels, 1 Hz. Error bars show mean ± 1 s.d. over ten simulations. **c**,**d**, Examples of NWD applied to optogenetically evoked inhibitory PSCs under single-target (**c**) and ten-target (**d**) holographic stimulation of PV neurons expressing ChroME2f. **d**, Shows responses to stimulating ensembles containing the neuron in panel **c**. **e**, Same as **d**, but for stimulation of SST neurons. **f**, Same as **c**, but for stimulation of pyramidal neurons expressing ChroME2s^16^ while recording from another pyramidal neuron.

The NWD network has a sequential U-Net architecture^20,21^ that uses one-dimensional (1D) convolutional filters to learn representations of the input signal at increasing levels of temporal compression. This allows the network to integrate information across the entire PSC trace, such that confounding synaptic currents preceding or following an admissible PSC ‘initiation zone’ (typically selected to be a window of 3–12 ms following stimulation) can be accurately subtracted and the baseline current reset to zero (Fig. 2a). As the network is trained to generate noise-free, demixed PSCs from tens of thousands of simulated noisy inputs, NWD also markedly improves the overall signal quality. Furthermore, unlike computationally intensive algorithms for deconvolving intracellular currents^22^, NWD can isolate the PSC evoked by optogenetic stimulation with just a simple forward pass through the network, with the resulting demixed PSCs usable in any connectivity inference algorithm and in real time.

**Extended Data Fig. 3:**
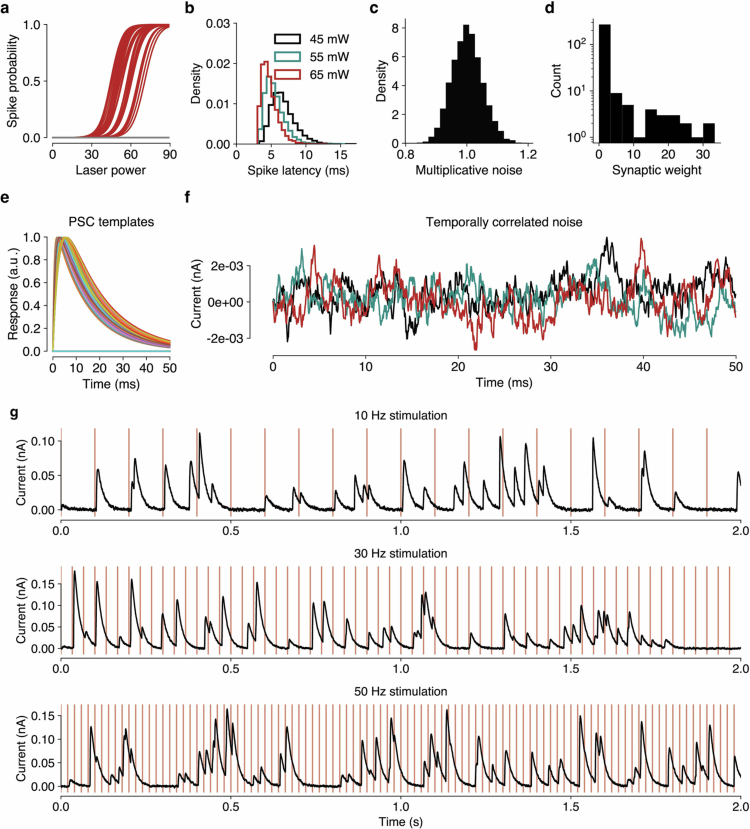
Components of an example simulated connectivity mapping experiment. **a**, Presynaptic spike probabilities as a function of laser power. Connected neurons shown in red, disconnected neurons shown in gray. **b**, Example histograms (shown as normalized densities) of presynaptic spike latencies for three typical laser powers. Average spike and transmission times decrease quadratically with increasing power. **c**, Example log-normal distribution of multiplicative noise terms that induce trial-to-trial variability in PSC amplitude. **d**, Distribution of synaptic weights, shown on log scale. **e**, Example PSC templates. Disconnected neuron templates represented by the zero vector. **f**, Three example noise processes sampled from a first-order autoregressive process. **g**, Example connectivity mapping experiment under increasing rates of stimulation. Simulation has 300 neurons with 10% connection probability and spontaneous synaptic currents occurring at 5 Hz.

Performing optogenetic connectivity mapping experiments at high speed poses a trade-off: the experiment can be completed more quickly, but measurements of the postsynaptic response are more confounded by currents from previous and subsequent trials. For this reason, a typical rate of stimulation for a connectivity mapping experiment is ~10 Hz (stimulating a neuron approximately every 100 ms (refs. ^5,6,23^), much longer than the decay time of a typical PSC), which we take as a baseline rate in later method comparisons. To characterize the improvement in experiment speed-up resulting from the application of NWD, we simulated connectivity mapping experiments at full 20-kHz sampling resolution with varying synaptic connection densities and stimulation frequencies (Fig. 2b and Extended Data Fig. 3). In a sparsely connected circuit, the primary action of NWD is to eliminate spontaneous synaptic currents and reduce electrical noise, leading to just a marginal improvement in signal fidelity compared to the raw PSC traces (Fig. 2b; 0.1 connection density example). However, as synapses become more prevalent the occurrence of postsynaptic events greatly increases, with many confounding synaptic currents elicited from previous trials. Application of NWD therefore leads to a substantial improvement in the signal fidelity of the postsynaptic response at high stimulation frequencies (Fig. 2b; 0.2–0.4 connection density examples). Similarly, the fidelity of the postsynaptic response degrades as a function of the number of simultaneously stimulated neurons and the background rate of spontaneous synaptic currents (Fig. 2b; middle and right); however, NWD largely eliminates this effect, and thus reduces experimental constraints on the duration of interstimulus intervals as confounding synaptic currents are substantially reduced. Stimulation can therefore be increased to rates closer to the refresh rate of the SLM. In practice, this enables us to stimulate much faster than the ~40-ms decay time typical of PSC traces in our preparations (for example Fig. 2c–f).

We applied NWD to each of our experimental settings, including demixing of inhibitory PSCs from PV and somatostatin (SST) mapping experiments under both holographic single-target and ensemble stimulation (Fig. 2d,e), as well as demixing of excitatory PSCs from pyramidal mapping experiments (Fig. 2f). We found that we could improve the performance of the NWD network by tuning it to the time constants of either inhibitory or excitatory synaptic currents (Methods and Supplementary Figs. 1–3). In each case the NWD network led to a substantial reduction in confounding synaptic currents and electrical noise, in agreement with our simulated results (Fig. 2b).

### Ensemble stimulation can test for many synaptic connections simultaneously

**Fig. 3:**
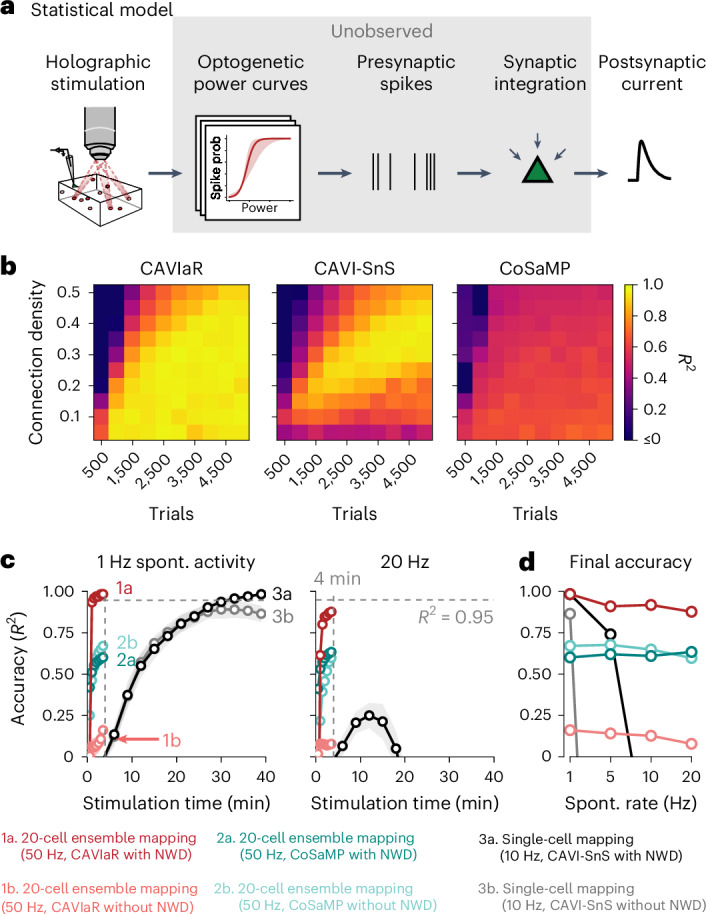
CAVIaR accurately maps synaptic connectivity at high speed in simulation. **a**, Statistical model relating patterns of holographic stimulation to measurements of postsynaptic current via latent optogenetic power curves, presynaptic spikes, and synaptic weights. **b**, Comparison of CAVIaR (ours, left) with CAVI-SnS (middle) and CoSaMP (right) for varying connection densities as a function of increasing amounts of experimental data. Simulated presynaptic candidate population size of *n* = 300 (hence, for example, 4,500 trials corresponds to an average of 15 stimuli per neuron). All PSCs are demixed using NWD. Note that CoSaMP requires the number of desired connections to be specified in advance; we therefore provided CoSaMP with the true number of connections, such that the reported performance represents an upper bound. *R*^2^ represents the coefficient of determination between the ground-truth and inferred connectivity vectors. Simulated neurons do not explicitly coincide with a specific genetically defined cell type, but could match various cell types depending on the simulated connectivity rate. **c**, Order of magnitude improvement in mapping efficiency using NWD-accelerated connectivity mapping with ensemble stimulation. Population size, 1,000. Connection density, 0.1. Left, convergence speed of CAVIaR, CoSaMP and CAVI-SnS with and without NWD, assuming a background rate of spontaneous PSCs of 1 Hz. In this simulation, while CAVI-SnS requires more than 30 min of stimulation to reach an accuracy of 0.95, CAVIaR requires just 30 s. A high rate of spontaneous activity (20 Hz, right) causes fast degradation of CAVI-SnS performance due to an accumulation of false positives, but impacts CAVIaR much less. In both conditions CoSaMP does not converge to the true connectivity. Note that given additional experiment time the accuracy of CAVIaR without NWD substantially improves, but does not match CAVIaR with NWD (Extended Data Fig. 5d). Error bars show mean ± 1 s.d. over 20 simulations. **d**, Final accuracy of all methods as a function of spontaneous PSC rate. CAVIaR is robust against spontaneous PSCs, unlike CAVI-SnS. Spont., spontaneous.

We sought to capitalize on recent technological developments in opsin engineering^2,3,14,16^, two-photon holography^17,24–26^ and fundamental mathematical results on efficient signal reconstruction in sparse settings^27–29^ by developing a statistical method for inferring synaptic connectivity from holographic ensemble stimulation. This was based on the logic that pairing rapid stimulation and computational demixing with the ability to test for many potential connections with each hologram could dramatically speed up circuit mapping. We therefore created and tested a variety of connectivity inference algorithms, and ultimately found that a variational inference approach for a hierarchical Bayesian statistical model was able to overcome the limitations of existing compressed sensing approaches. Our model relates patterns of holographic stimulation to PSCs through a series of key latent variables: (1) optogenetic ‘power curves’ that characterize the relationship between laser power and presynaptic spike probability due to variation in opsin expression and rheobase across neurons; (2) presynaptic spikes successfully elicited by photostimulation and transmitted to the postsynaptic neuron; and (3) synaptic weights that determine the amplitude of the resulting PSCs (Fig. 3a). We learn posterior distributions over the latent variables using CAVIaR, an algorithm that iteratively updates the parameters of a variational approximation to the posterior such that the inferred presynaptic spikes respect the biophysical plausibility constraint of having isotonically increasing spike probabilities as a function of laser power (namely, such that the probability of evoking a presynaptic spike increases with laser power on average^16^; see Methods).

A key component of our experimental design was to randomly switch between three or more laser powers while mapping. As CAVIaR infers presynaptic spikes, we could then estimate optogenetic power curves to determine whether the plausibility constraint was satisfied. Any putative presynaptic neurons for which spike counts happen to implausibly decrease with increasing power are considered spurious, and immediately disconnected by CAVIaR. When using our software in practice, a user simply sets a parameter that imposes a minimum success rate (between 0 and 1) for evoking presynaptic spikes (given by the isotonically constrained power curve at maximal power) and CAVIaR identifies putatively connected neurons meeting that criterion; however, PSCs can occur spontaneously, inflating the estimated presynaptic spike rate and increasing the number of false positives. We therefore simultaneously estimate the background rate of spontaneous currents and use this to adaptively adjust the plausibility criterion during inference (Supplementary Fig. 4).

**Extended Data Fig. 4:**
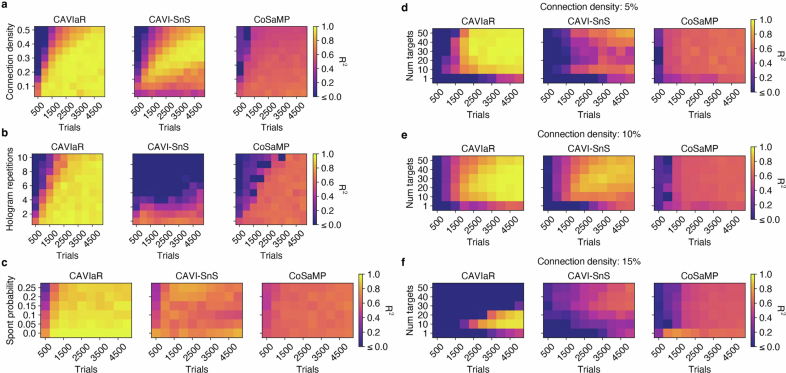
Performance and phase transitions for three connectivity inference techniques. **a**, Performance as a function of underlying density of synaptic connectivity (connection probability). Same as Fig. 2b. **b**, Performance as a function of stimulus diversity (hologram repetitions). **c**, Performance as a function of probability of spontaneous PSC lying within admissible PSC initiation window. Fraction of connected neurons in (**b**)-(**c**), 0.1. Note that in order to illustrate the behavior of the three algorithms, these simulations involve varying only one parameter at a time while the others are kept fixed. However, the precise shape of these heatmaps could change if parameters are varied jointly. Default parameters: connection density, 0.1; hologram repetitions, 1; spontaneous PSC probability, 0.05. **d-f**, The optimal number of targets for CAVIaR, CAVI-SnS, and CoSaMP depends on the density of synaptic connectivity. In these simulations, number of presynaptic candidate neurons, 1000; spontaneous PSC probability, 0.05; number of hologram repetitions, 1. When mapping populations with a 10% connection density, 20-target stimulation is optimal (**e**). However, at 5% connection density this increases to 30-40 targets (**d**), and at 15% connection density this reduces to 10 targets (**f**).

We compared CAVIaR to two other approaches. (1) Standard compressed sensing. While there are a number of widely used algorithms for compressed sensing^30^, they each solve a common mathematical problem and therefore lead to closely related results. Compressive sampling matching pursuit (CoSaMP^31^) is a particularly scalable algorithm for solving the compressed sensing problem and was used in the first simulation study on compressive connectivity mapping^8^, which we therefore benchmark CAVIaR against and consider representative of the performance from related studies (for example, ref. ^23^). (2) A variational inference technique for a spike-and-slab connectivity model similar to CAVIaR but that, among other methodological differences, does not impose isotonic regularization or account for spontaneous synaptic currents (CAVI-SnS^11^). We evaluated the ability of each method to reconstruct connectivity in simulations where the sparsity of the underlying connectivity varied, as compressed sensing-based techniques are known to be particularly sensitive to this parameter^27^. We found that CAVIaR greatly outperformed conventional compressed sensing (Fig. 3b; left versus right), primarily because the latter does not have a model for stochastic, power-dependent presynaptic spikes. Notably, in line with earlier results^8^, we found that algorithms failing to account for this stochasticity will yield strongly biased synaptic weight estimates as they will average over postsynaptic measurements when no presynaptic spikes occurred. CAVIaR also outperformed CAVI-SnS, which transiently demonstrated high accuracy, but ultimately generated increasingly worse estimates of connectivity with increasing amounts of data as it lacked mechanisms to prevent spontaneous PSCs from being misidentified as false positives. We found that CAVIaR consistently achieved state-of-the-art performance when repeating the analysis while varying the number of times each stimulus was repeated, the number of simultaneous targets, and the rate of background spontaneous activity as a function of the amount of data collected (Extended Data Fig. 4 and Supplementary Fig. 5).

**Extended Data Fig. 5:**
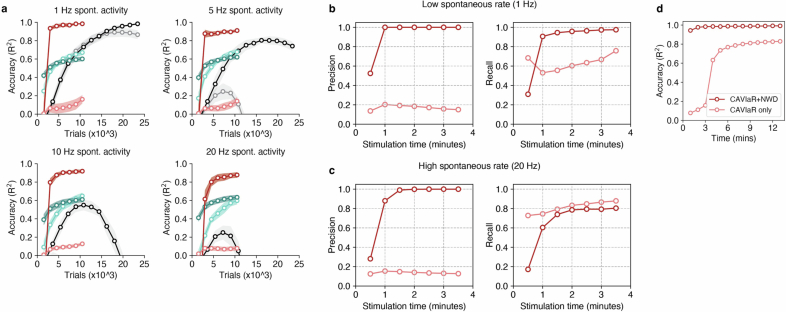
Additional convergence rates in simulation. **a**, Cf. Fig. 2c, data replotted as a function of trials. While mapping as a function of stimulation trials neglects the critical speed-up enabled by NWD, CAVIaR nevertheless shows a substantial improvement in mapping efficiency. **b**, Convergence of precision and recall using CAVIaR in simulations with low rates of spontaneous PSCs (1 Hz). **c**, Same as (**b**), but with a 20 Hz rate of spontaneous PSCs. Population size, 1000; connection density, 10%; number of simultaneously stimulated targets, 20; stimulation frequency, 50 Hz. Dark line represents performance of CAVIaR with NWD; light line, without NWD. Note that recall is higher in (**c**) without NWD due to an excessive number of neurons being declared connected (cf. precision <0.2 for CAVIaR without NWD). Each data point is an average over 10 simulations. **d**, Performance of CAVIaR without NWD for longer experiment time shows eventual performance improvement, but does not match performance with NWD. Each data point is an average of 10 simulations.

Next, we estimated the speed-up obtained by combining rapid ensemble stimulation with NWD and CAVIaR, and compared this against previously established techniques. Our simulations showed that even with a large population size (in this case mapping 1,000 potential presynaptic neurons), the speed of connectivity mapping was optimal when stimulating ensembles of 20 neurons at a time, beyond which the accuracy of presynaptic spike inference gradually degraded, though this also depended on the density of synaptic connectivity (Extended Data Fig. 4). Our speed-up simulations were therefore performed using 20-neuron stimulation. We anticipated that the background rate of spontaneous PSCs would also be a key factor influencing the ultimate accuracy of each connectivity inference method, as a high number of spontaneous PSCs that happen to fall within the stimulation window could mimic a synapse and lead to false positives. Single-target mapping at 10 Hz with CAVI-SnS required more than 30 min of stimulation to cross an accuracy of 0.95 in simulations with 1,000 neurons, 10% connectivity, and 1 Hz spontaneous activity (Fig. 3c, left; accuracy measured as the *R*^2^ between the true and estimated synaptic weights). By comparison, NWD-enabled 50-Hz stimulation of 20-neuron ensembles recovered synaptic connectivity at an accuracy exceeding 0.95 with just 30 s of stimulation using CAVIaR, resulting in a rate of connectivity inference of more than 2,000 neurons per minute in low spontaneous activity conditions (exceeding an order of magnitude more neurons tested per minute compared to single-target stimulation with CAVI-SnS). In the same time period, CoSaMP obtained an accuracy of less than 0.6, and in our simulations never achieved an accuracy of 0.95 regardless of stimulation time (Fig. 3c,d and Extended Data Fig. 4). We also confirmed that similar improvements in mapping efficiency are obtained when accuracy is evaluated as a function of stimulation trials, rather than time (Extended Data Fig. 5a).

**Extended Data Fig. 6:**
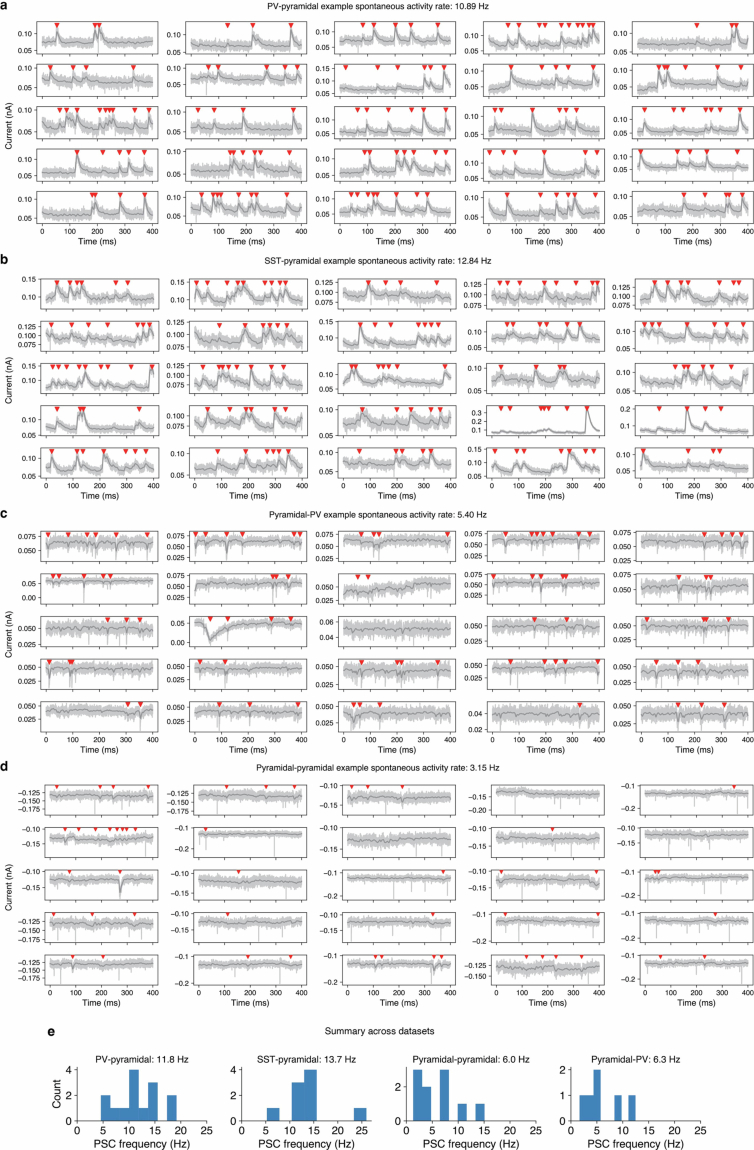
Determination of spontaneous PSC rates across all cell-type combinations used in this study. **a-d**, Example detected spontaneous PSCs (red triangles) from 400 ms windows of intracellular recordings without stimulation. Panels show only a subset of trials without stimulation to facilitate visualization. **e**, Distribution of spontaneous PSC frequencies across all datasets used in this study. Panel headings show corresponding averages. Note that different spontaneous PSC rates can arise due to the different holding potentials used for the patch-clamped neuron.

We then increased the rate of spontaneous PSCs to 20 Hz, a more challenging regime in which the spontaneous PSC rate exceeded those in our own mapping experiments (Extended Data Fig. 6). This revealed several important behaviors of CAVIaR and CAVI-SnS (Fig. 3c, right; Fig. 3d). Namely, the accuracy of CAVI-SnS both with and without NWD behaved non-monotonically (similar to Fig. 3b, final *R*^2^ at a 20 Hz spontaneous rate with and without NWD < 0), with connectivity estimates ultimately getting worse with additional experiment time due to the accumulation of spontaneous PSCs. However, CAVIaR with NWD obtained nearly the same accuracy when spontaneous PSCs occurred at 20 Hz as it did when they occurred at 1 Hz due to CAVIaR’s built-in mechanisms for estimating and adapting to spontaneous PSCs (final *R*^2^ with spontaneous rate 1 Hz, 0.98; 20 Hz, 0.88; Fig. 3c,d). Similarly, CAVIaR with NWD achieved high precision and recall in simulations with a spontaneous PSC rate of 20 Hz (final precision, 1; recall, 0.8; Extended Data Fig. 5b,c). These results thus indicate that our model-based compressed sensing approach should be robust in conditions with high spontaneous activity.

### Validating CAVIaR using mapping experiments in cortical slices

**Fig. 4:**
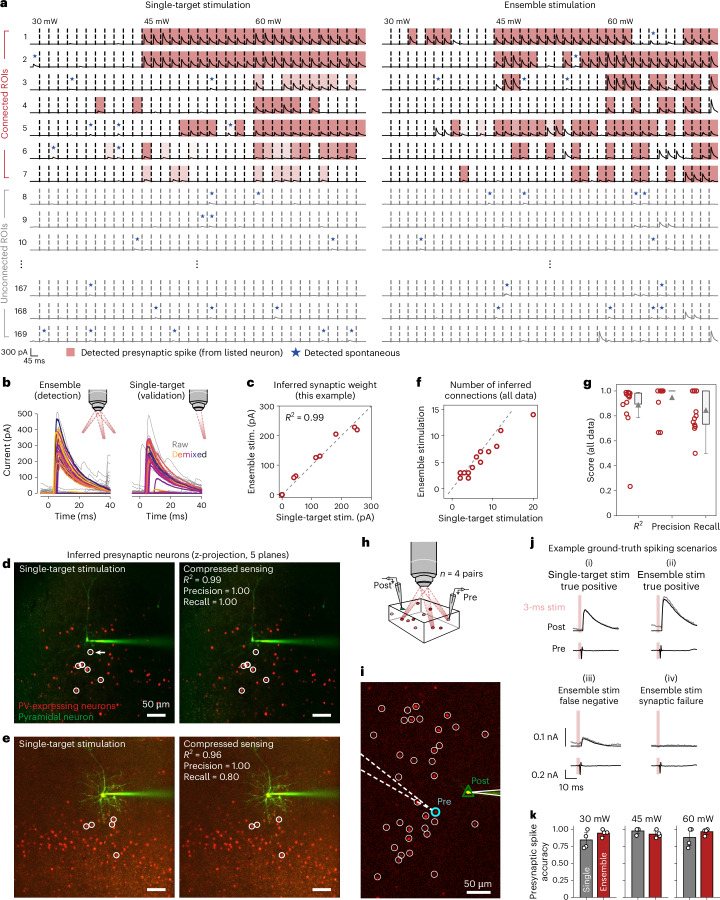
Validation of model-based compressed sensing approach for mapping PV–pyramidal connections. **a**, ‘Checkerboard’ visualization of CAVIaR inferences. Left column shows demixed PSCs evoked by single-target stimulation of the listed ROI (‘target’). Right column shows PSCs evoked by holographic stimulation of ensembles containing the listed ROI. Trials separated by dashed lines and rearranged to be in order of increasing laser power (from 30 mW to 60 mW). While all single-target stimulation trials are shown on the left, for ease of visualization only a fraction of ensemble stimulation trials are shown on the right. Shaded trials indicate when CAVIaR detects that the observed PSC was evoked by successfully spiking the listed (presynaptic) ROI; opacity of shading represents the estimated posterior uncertainty of the spike (lighter, less certain; darker, more certain). Blue stars represent when CAVIaR determines that the PSC is spontaneous (due to, for example, an uncharacteristic amplitude). Note that responses are only shaded when the neuron in the listed row actually spiked (as determined by CAVIaR). PSC waveforms that are not shaded and not labeled as spontaneous can still be attributed to other neurons that were stimulated in the same ensemble. Out of 169 stimulated ROIs, the same set of 7 were identified by CAVIaR as being presynaptic by both single-target and ensemble stimulation. **b**, Example PSCs (corresponding to neuron marked by white arrow in **d**, below) evoked by holographic stimulation of ensembles containing a putatively connected neuron (left). Single-target stimulation of the same neuron validates the existence of the individual synapse (right). Gray traces show raw PSCs, colored traces show demixed PSCs. Traces colored at random. **c**, Synaptic weights estimated by CAVIaR independently from single-target stimulation and ensemble stimulation trials show strong agreement (*R*^2^, 0.99). Seven synaptic connections shown; points at zero indicate no connection. **d**, *Z*-projection (over five different planes) of stimulation FOV for experiment corresponding to **a**–**c**. White circles show identified presynaptic neurons. Left, presynaptic neurons identified using single-target stimulation; right, presynaptic neurons identified using ensemble stimulation. NWD and CAVIaR were used in both cases. Seven connections are found using both single-target stimulation and ensemble stimulation. Note that one connection is hidden by another on a higher plane and therefore not directly visible. **e**, Additional example of similarity between synaptic connectivity maps obtained using single-target (left) vs ensemble stimulation (right) in combination with NWD and CAVIaR. **f**, Number of connections identified by CAVIaR using both single-target and ensemble stimulation across 14 experiments. Dashed gray line shows identity. **g**, Agreement between 14 connectivity maps obtained using single-target stimulation compared to compressed sensing. Agreement measured using the *R*^2^ (which accounts for synaptic strength; mean ± 1 s.d., 0.89 ± 0.19) and the precision and recall (which measures agreement using only a binary classification; mean ± 1 s.d.; precision 0.95 ± 0.12, recall 0.84 ± 0.15). Bounds of box plots represent interquartile range, triangles show the mean, and whiskers represent lower and upper limits (excluding outliers). **h**, Joint whole-cell and cell-attached recordings provide ground-truth presynaptic spikes associated with postsynaptic responses during single-target and ensemble stimulation. **i**, Example image showing locations of presynaptic and postsynaptic neurons, among other segmented ROIs. **j**, Example scenarios from a paired recording associated with successful optogenetic generation of presynaptic spikes. While trials (i) and (ii) resulted in correct predictions of presynaptic spiking by CAVIaR (are true positives), trials (iii) and (iv) were declared trials on which the stimulated neuron did not spike, either due to an unusually low-amplitude PSC or synaptic failure. Underlaid gray traces show raw PSC without NWD, black traces show PSC with NWD. Scales at the bottom left apply to all subpanels. **k**, Performance of presynaptic spike inference over *n* = 4 paired recordings, separated by single-target versus ensemble stimulation and laser power. Error bars show mean ± 1 s.d. over four data points. Performance of presynaptic spike inference does not depend on power or single-target versus ensemble stimulation (*P* > 0.05 for all pairs, dependent *t*-test), though lack of significance could be due to sample size.

**Extended Data Fig. 7:**
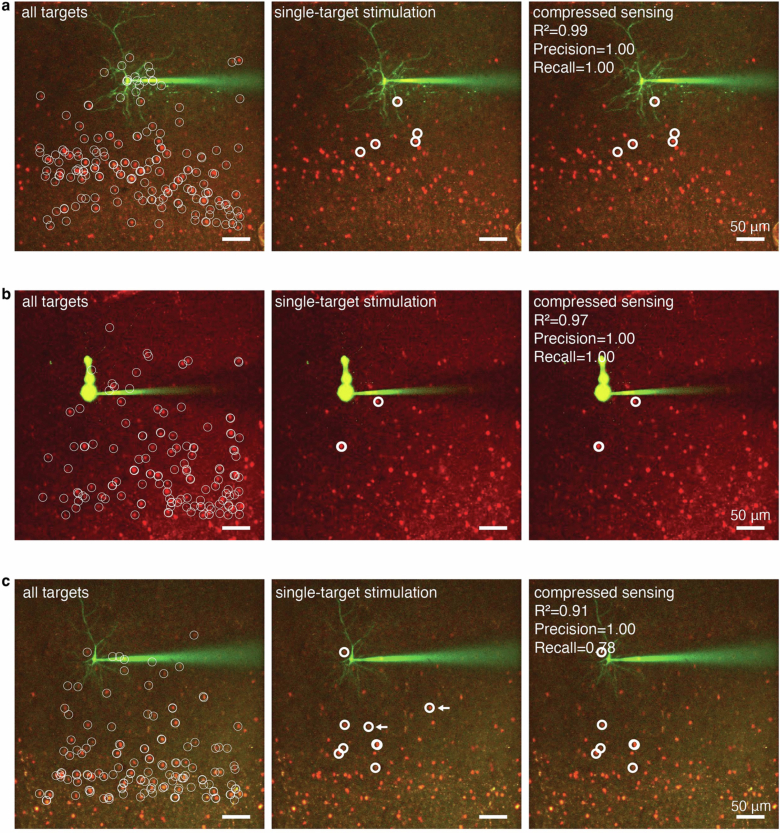
Additional comparisons between connectivity maps obtained using single-target and ensemble stimulation. Panels (**a**)-(**c**) show example maps from three different cortical slices. Arrows in panel (**c**) show connections found using single-target stimulation that were not identified using ensemble stimulation.

Having established the accuracy and speed of our connectivity mapping system in simulations, we next sought to validate our computational tools experimentally. We first wanted to confirm that connectivity maps obtained using ensemble stimulation were closely aligned to those obtained using the existing approach of single-target stimulation. We therefore performed experiments where the same population of PV neurons was mapped using randomly interleaved trials of single-target and holographic ensemble stimulation (ensembles of ten neurons, stimulation performed at 30 Hz). We separated trials into two sets depending on whether they were obtained using single-target or ensemble stimulation, demixed the optogenetically evoked PSCs using NWD, and detected putative synapses in each set independently using CAVIaR (Fig. 4a–d). For the example shown in Fig. 4a, model-based compressed sensing identified the same set of synaptically connected PV neurons as with single-target stimulation and almost identical synaptic strengths (varying from 57 to 228 pA; Fig. 4c,d). Across 14 different experiments we probed 2,619 total presynaptic candidates (mean number of targets probed, 187; minimum, 107; and maximum, 269). CAVIaR identified very similar numbers of connections when using single-target stimulation compared to ensemble stimulation (Fig. 4e,f; *R*^2^, 0.81). Further, we confirmed that these were primarily the same set of connections when evaluated based on both their numerical synaptic strengths (Fig. 4g, left; average *R*^2^ between connectivity vectors estimated using single-target and ensemble stimulation, 0.89), as well their binary classification (whether a region of interest (ROI) was connected or not, without regard to synaptic strength; Fig. 4g, right; average precision, 0.95; and average recall, 0.84). Additional examples are given in Extended Data Fig. 7.

We next wanted to confirm that CAVIaR’s inferences are well calibrated. Specifically, as compressive approaches to connectivity mapping depend closely on the accuracy of the presynaptic spike inference step^8,11^, we wanted to determine whether the spikes inferred by CAVIaR were allocated to the correct presynaptic neurons. We therefore performed multiple (*n* = 4) paired recordings to obtain ground-truth spikes. We first used single-target stimulation to identify a likely presynaptic neuron in real time, then established a cell-attached patch clamp on the presynaptic neuron, and then randomly alternated between single-target and ensemble stimulation across multiple power levels while recording presynaptic spikes (Fig. 4h,i). We confirmed that each of the neurons identified as connected using single-target stimulation following establishment of the patch clamp were also considered connected when the population was subsequently mapped using ensemble stimulation.

Paired recordings revealed a range of different scenarios associated with ground-truth spiking (Fig. 4j). This included true positives, where CAVIaR correctly inferred a presynaptic spike from a given PSC evoked by single-target or ensemble stimulation (scenarios (i) and (ii) in Fig. 4j). This also included false negatives, however. For example, in some cases CAVIaR incorrectly estimated that an unusually small PSC did not arise by a stimulation-evoked spike of the patched presynaptic neuron but rather from another, more weakly connected neuron that was stimulated at the same time (scenario (iii)). False negatives also occurred due to synaptic failure, where the presynaptic neuron successfully elicited, but did not transmit, a spike (scenario (iv)). Despite the latter two challenging scenarios, CAVIaR achieved high accuracy in its overall estimates of presynaptic spikes with no significant degradation in its ability to infer presynaptic spikes for individual neurons, even when stimulating ten neurons at once and over a range of laser powers (Fig. 4k; all pair-wise differences not significant, *P* > 0.05, dependent *t*-test).

### CAVIaR estimates converge rapidly and accurately predict postsynaptic responses to novel holographic stimulation patterns

To go beyond single-target confirmation of the presence and magnitude of the synapses identified using CAVIaR (Fig. 4f,g), we next asked whether the inferred connectivity could be used to predict the postsynaptic response to holograms targeting combinations of neurons that the model had never seen before. We also wanted to know whether ensemble stimulation was systematically engaging polysynaptic effects. For example, PV neurons are known to provide high levels of mutual inhibition^32^, which could be triggered when stimulating many PV neurons at once. In this case, other PV neurons would be inhibited, potentially reducing the number of driven PV neurons and resulting in smaller inhibitory PSCs than would be expected by CAVIaR.

**Fig. 5:**
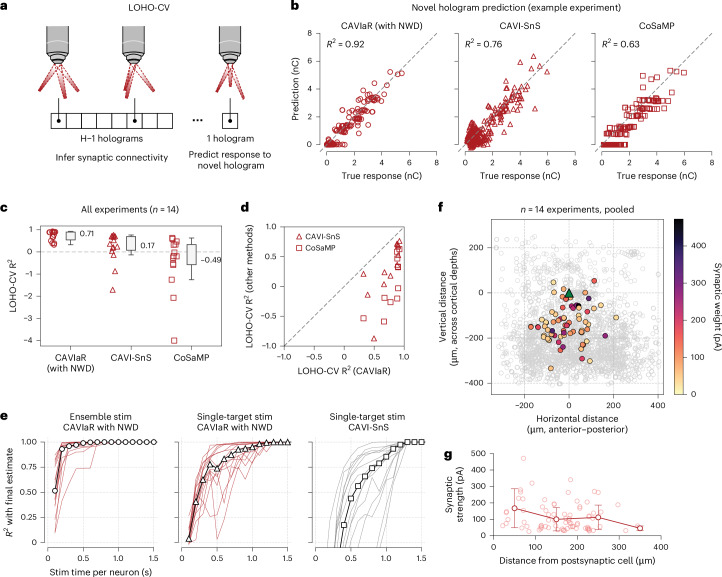
Predictive performance and convergence rates using model-based compressed sensing outperform existing methods. **a**, Leave-one-hologram-out cross-validation can be used to determine the predictive performance of connectivity mapping techniques. **b**, LOHO-CV *R*^2^ scores for CAVIaR, CAVI-SnS and CoSaMP on an example mapping experiment, showing that CAVIaR obtains the highest accuracy. No method suggests recruitment of polysynaptic effects, which would manifest as systematic off-diagonal data points. For these comparisons, CoSaMP was provided with the number of connections identified by CAVIaR. **c**, Summary of LOHO-CV *R*^2^ scores across 14 different PV–pyramidal mapping experiments. Dashed gray line denotes an *R*^2^ of 0; scores below this line indicate poorer predictive performance than simply predicting the mean of the true responses without regard to which neurons were stimulated. **d**, Direct comparison of CAVIaR LOHO-CV *R*^2^ scores to CAVI-SnS and CoSaMP, showing that CAVIaR outperforms both methods on each dataset. **e**, Convergence time for proposed model-based compressed sensing technique (ensemble stimulation with CAVIaR and NWD) compared to conventional single-target mapping. Stimulation performed at 30 Hz. Ensemble stimulation applied to ten targets at once. Total convergence time for each experiment calculated as the time per trial (33 ms for a 30-Hz stimulation speed) multiplied by the number of trials. For each experiment, the convergence time per neuron is obtained by dividing the total convergence time by the number of neurons in the experiment. Convergence time reported per neuron due to varying population sizes (larger populations take longer to map). Red/gray lines show convergence times for individual experiments. Each point is obtained by evaluating the *R*^2^ between the estimated connectivity at that time point and at the end of the experiment. Black line with markers shows median across experiments. **f**, *Z*-projected map of connection strength across 14 pooled experiments. Colored circles indicate position of identified presynaptic neurons, open gray circles indicate positions of all probed ROIs. Individual maps aligned to have postsynaptic neuron at location (0, 0). **g**, Decreasing synaptic strength as a function of distance from the postsynaptic neuron (14 experiments, pooled). A similar trend holds for connection probability (Supplementary Fig. 7). Faint circles show the strength of each connection; dark lines show mean ± 1 s.d. for connection strengths binned at 100 μm.

To simultaneously answer these questions, we performed an out-of-sample testing method that we refer to as leave-one-hologram-out cross-validation (LOHO-CV; Fig. 5a). Assuming that *H* different holograms are used in the experiment (where each hologram targets a different set of neurons), LOHO-CV works by running the CAVIaR algorithm on data corresponding to *H* − 1 holograms, such that the algorithm has never observed postsynaptic responses from simultaneous stimulation of the neurons targeted in the *H*th hologram. Then, samples from the posterior distribution over the model parameters are used to predict the mean postsynaptic response to hologram *H* at three different power levels, a process that includes sampling over the model’s uncertainty about whether spikes will be elicited by stimulation at any given laser power. The predicted response is compared against the held-out response, and the process continues with a different hologram selected to be held out until the response to every hologram has been predicted.

LOHO-CV confirmed that CAVIaR achieved high accuracy in predicting responses to novel holograms (Fig. 5b; example *R*^2^, 0.92; average over 14 experiments, 0.71). This indicated that CAVIaR did not miss synaptic connections that evoked large and reliable PSCs (such missed connections would appear as data points along the *y* = 0 axis in Fig. 5b). Additionally, high LOHO-CV performance implied that CAVIaR did not make systematic errors arising due to polysynaptic effects, which would manifest as clear off-diagonal points in Fig. 5b. We performed a similar cross-validation analysis for CAVI-SnS and CoSaMP, which achieved substantially lower performance compared to CAVIaR (Fig. 5b, middle and right). Repeating the analysis across all 14 experiments showed that these two previously developed techniques exhibited systematic errors in their ability to predict responses to unseen holographic stimuli (Fig. 5c,d), indicating that they did not learn connectivity consistent with the underlying physiology.

We next wanted to determine how efficient our protocol was for discovering synaptic connectivity as a function of continuous stimulation time (namely, without regard to experiment time devoted to pausing stimulation to measure access resistance, adjusting the seal of the patch pipette, etc.). To do so, we subsampled mapping data from the total set of available trials and, for each subset, applied NWD and CAVIaR to estimate connectivity. This allowed us to monitor the rate of convergence of CAVIaR to its final estimates in increments of tens of seconds. Note that because our principal aim was validation of the connectivity inferences themselves rather than a demonstration of speed-up specifically, each hologram was presented multiple (three) times at random throughout the experiment. This experimental design provided a means to characterize performance via LOHO-CV analysis, but could only establish a lower bound on mapping speed because simulations demonstrate that optimal speeds are obtained only when unique holograms are used on every trial (Extended Data Fig. 4b). Nevertheless, we found that our mapping protocol converged to an *R*^2^ of 0.9 with less than just 200 ms of stimulation time per neuron at 30 Hz (Fig. 5e, left plot; obtained by normalizing mapping time by number of neurons in experiment). By comparison, single-target stimulation using CAVIaR and NWD reached an *R*^2^ of 0.9 in 800 ms per neuron (though with much higher variance; Fig. 5e, middle plot), and single-target stimulation using the existing approach of CAVI-SnS required 1.1 s per neuron (Fig. 5e, right plot), more than five times slower than model-based compressed sensing and (in accordance with our simulation and LOHO-CV results; Fig. 3c and Fig. 5c) with substantially lower predictive accuracy.

We also repeated the analysis by plotting the rate of convergence using single-target mapping and compressed sensing, but as a function of the number of times each neuron is stimulated and as a function of the number of stimulation trials instead of in units of time (Supplementary Fig. 6). This showed that single-target mapping converges in slightly fewer stimulations per neuron than compressed sensing (Supplementary Fig. 6a–c), presumably because CAVIaR must solve a spike inference problem that is challenging for ensemble stimulation but straightforward for single-target stimulation. However, when plotted as a function of trials (Supplementary Fig. 6d–f) and as a function of trials normalized by population size (Supplementary Fig. 6g–i), compressed sensing converged considerably faster, as neurons are stimulated far more times over the course of an experiment using ensemble stimulation.

Aggregating the connections resulting from the use of ensemble stimulation, NWD and CAVIaR recapitulated the characteristic^5^ distance-dependent structure of PV–pyramidal connectivity (Fig. 5f,g and Supplementary Fig. 7).

### Inference of synaptic connectivity from ensemble stimulation across multiple cortical cell types

**Fig. 6:**
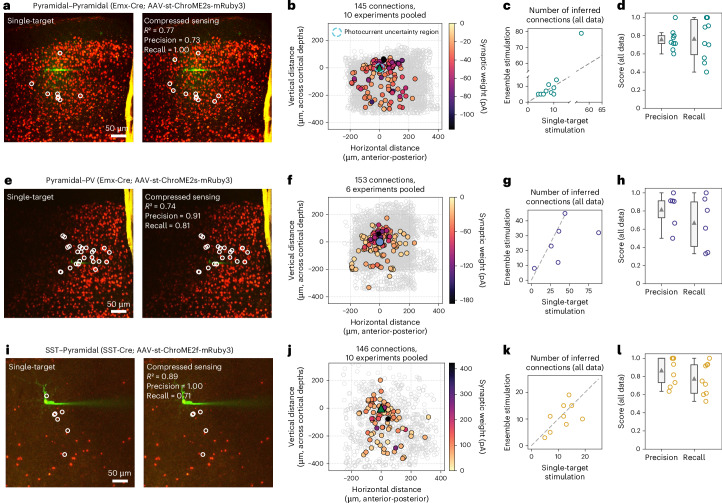
Model-based compressed sensing of synaptic connectivity applied to multiple cortical cell-type combinations. **a**, Comparison of pyramidal–pyramidal connectivity maps obtained on the same population of neurons using single-target stimulation and model-based compressed sensing (ten-target ensembles, 30-Hz stimulation, connections identified using CAVIaR with NWD). Experiment performed over five planes, image shows *z*-projection. Agreement between synaptic weights estimated by CAVIaR independently from single-target stimulation and ensemble stimulation trials. **b**, Map of synaptic connections identified using ensemble stimulation across ten pooled experiments. Dashed cyan circle represents region with 30 *μ*m radius where synaptic connections are most uncertain due to potential photocurrent contamination in opsin-positive postsynaptic neurons (however, see Extended Data Fig. 9 and Supplementary Fig. 9 for the suppressive effect of NWD). Identified connections split by plane shown in Supplementary Fig. 10. **c**, Number of connections identified by CAVIaR using both single-target and ensemble stimulation across ten experiments. Dashed gray line shows identity. **d**, Agreement between connectivity maps obtained using single-target stimulation compared to model-based compressed sensing. Mean precision, 0.76; mean recall, 0.77. Gray triangles indicate mean values. **e**–**h**, same as **a**–**d**, but for mapping pyramidal–PV connections over six experiments. Blue circle represents postsynaptic PV neuron. Mean precision, 0.82; mean recall, 0.67. Lower recall for pyramidal–PV connections could arise due to the faster and more irregular PSC kinetics encountered compared to PV–pyramidal connections. **i**–**l**, Same as **a**–**d**, but for mapping SST–pyramidal connections over six experiments. Mean precision, 0.87; mean recall 0.78. For the box plots in **d**,**h**,**l**, the bounds of the boxes represent the interquartile range, the triangles show the mean and the whiskers represent the lower and upper limits (excluding outliers).

**Extended Data Fig. 8:**
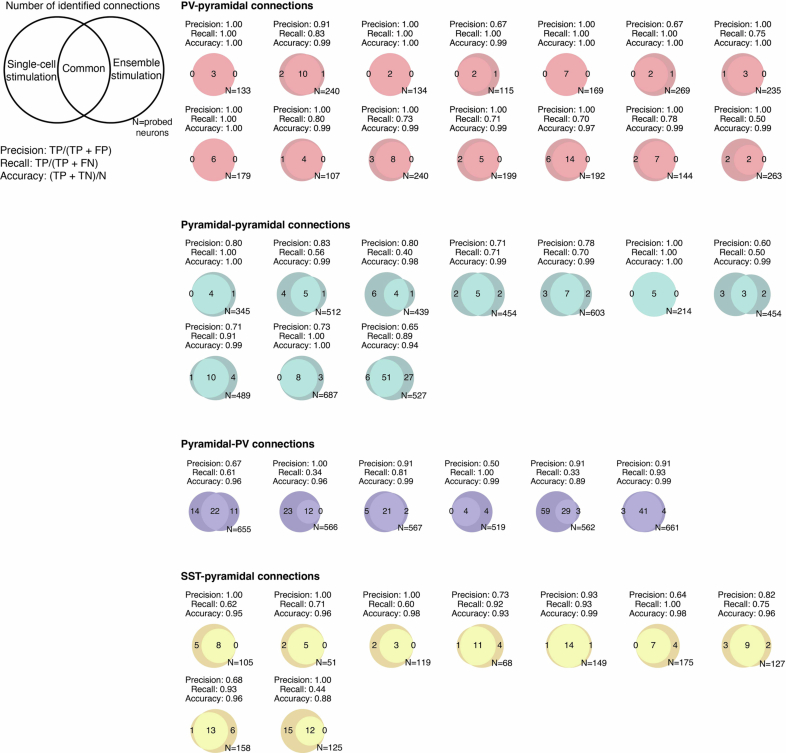
Comprehensive overview of CAVIaR performance for all mapping experiments used in this study. Top-left: The performance of CAVIaR is measured using the precision (fraction of connections identified using ensemble stimulation that are “true” connections), recall (fraction of “true” connections that are correctly identified using ensemble stimulation), and accuracy (total fraction of correctly classified connections). Here “true” connections are those identified using single-target stimulation, though note that single-target stimulation can also yield false positives and false negatives. TP: true positive, TN: true negative, FP: false positive, FN: false negative, N: total number of probed targets.

Finally, we wanted to characterize how model-based compressed sensing performed across a variety of cortical cell-type combinations. We used an Emx-Cre line to express the more potent ChroME2s opsin in pyramidal neurons (which benefit from the greater potency of ChroME2s as the rheobase of pyramidal neurons can often be higher than interneurons, see for example the NeuroElectro database^33^) and an SST-Cre line to express ChroME2f in SST neurons. Then, in addition to our previous experiments mapping PV–pyramidal connections, we also mapped pyramidal–pyramidal (*n* = 10 experiments), SST–pyramidal (*n* = 9) and pyramidal–PV connections (*n* = 6, Fig. 6; for summary across all experiments see Extended Data Fig. 8). In each case we used our previous experimental design, where we randomly interleaved single-target and ensemble stimulation trials, and used NWD and CAVIaR to infer connectivity independently from each trial type.

**Extended Data Fig. 9:**
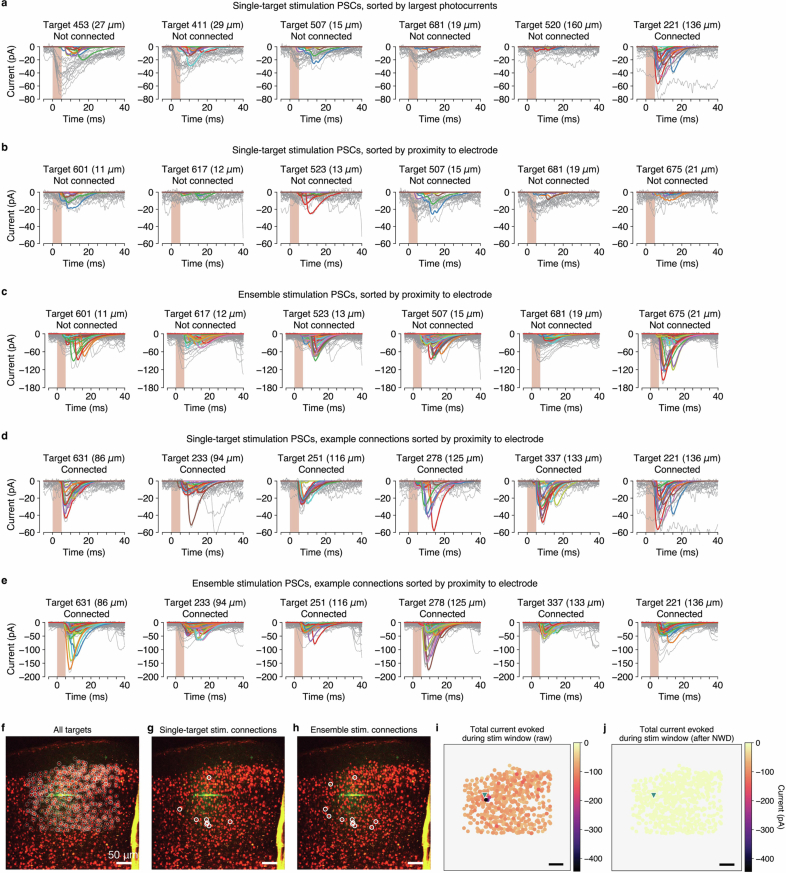
Analysis of the effect of NWD on direct photocurrent artifacts in pyramidal–pyramidal (Emx-Cre; AAV-st-ChroME2s-mRuby3) mapping experiments (corresponds to Fig. 6a). **a-e**, Photocurrents and postsynaptic responses for multiple conditions (single-target stimulation, ensemble stimulation), connection statuses (connected, not connected), and orders (sorted by largest photocurrents, or by proximity to electrode). Raw traces shown in gray, demixed traces shown in color. Numbers inside parentheses show distance from electrode tip. **f-h**, Stimulation FOVs showing all targets (**f**), connections inferred using single-target stimulation (**g**), and connections inferred using ensemble stimulation (**h**). **i, j**, Spatial representation of the magnitude of evoked currents during stimulation period before (**i**) and after (**j**) applying NWD. Magnitude given as the total summed current (in picoamperes) during the stimulation window (shaded red regions in **a-e**) across all stimulation trials and powers. Heightened currents near the electrode tip (base of green triangle) correspond to direct photocurrents on the patched cell.

Our experiments mapping pyramidal–pyramidal connections involved probing hundreds of potential presynaptic candidates per cortical slice (Fig. 6a,b; mean number of targets, 472; minimum, 214; maximum, 687). This experimental preparation posed a particular challenge because the high opsin expression density made it difficult to identify any pyramidal neurons that were entirely opsin-negative, and consequently we typically patched an opsin-positive postsynaptic pyramidal neuron (fraction of pyramidal–pyramidal mapping experiments with opsin-positive postsynaptic neuron, 9/10). When probing nearby presynaptic candidates we thus frequently encountered direct photocurrent artifacts in our whole-cell recordings that could affect our ability to discriminate synaptic connectivity (direct photocurrents could increase the false-positive rate in the immediate vicinity of the patch electrode); however, because NWD is trained to isolate PSCs occurring within a specified window (typically 3–12 ms after stimulation), confounding photocurrents (which begin rising immediately upon stimulation) were suppressed by the demixing process (Extended Data Fig. 9). Following the application of CAVIaR to infer connectivity, there was (as expected) some variability between connectivity maps obtained using single-target stimulation and model-based compressed sensing, but the identity, strength, and overall number of identified connections tended to be conserved across experiments (Fig. 6a,c,d; mean *R*^2^ between single-target and model-based compressed sensing connectivity vectors, 0.39; mean precision, 0.76; and mean recall, 0.77).

Mapping pyramidal–PV connections involved probing similar numbers of potential presynaptic candidates as in our pyramidal–pyramidal maps due to a similarly high opsin expression density and intrinsic cell-count (Fig. 6e; mean number of targets across six experiments, 588; minimum, 519; maximum, 661); however, because in this preparation opsin is expressed specifically in pyramidal neurons, when recording from PV neurons we faced no risk of direct photocurrent artifacts. Across six experiments, connectivity maps obtained using single-target stimulation and model-based compressed sensing achieved a mean *R*^2^ of 0.41, a mean precision of 0.82 and a mean recall of 0.67 (Fig. 6f–h).

Finally, we considered whether using compressed sensing to map connections from SST neurons to pyramidal neurons could pose an additional challenge because SST–pyramidal connections are largely axo-dendritic, potentially causing significant filtering and attenuation of PSCs through the dendrites of pyramidal neurons^34–36^, though previous work has shown the feasibility of using single-target stimulation (via two-photon glutamate uncaging) to map connections of this type^37^. Across nine experiments we found that, while some variability persisted, the connectivity maps obtained using single-target stimulation and model-based compressed sensing were highly conserved, despite this more challenging experimental setting (Fig. 6i–l; mean *R*^2^, 0.6; mean precision, 0.87; mean recall, 0.78; mean number of targets, 120; minimum, 51; and maximum, 175).

By performing a relatively small number of experiments (6–14) for each of the mapped populations, we acquired connectivity maps that corresponded to the probing of thousands of presynaptic candidates within the local volume. These maps recapitulated the increased connection probability in the proximity of the postsynaptic neuron (≤100 μm around the cell) characteristic of connectivity mapping studies (Fig. 6b,f,j), which typically require pooling together many more smaller-scale experiments^2,5,38,39^. While a detailed analysis of the spatial properties of individual connectivity maps is beyond the scope of this study, our results indicate the possibility of investigating map-to-map variability (Fig. 4d,e and Extended Data Fig. 7)^40^. Further, our results show the feasibility and utility of compressive connectivity mapping across a diversity of cortical cell-type combinations, despite challenges arising due to synaptic physiology or experimental preparation (for example, direct photocurrent artifacts).

## Discussion

Uncovering how neural computations are implemented in the cortex requires knowledge of the existence and strength of the underlying synaptic connections. We therefore developed a pair of complementary computational tools (NWD and CAVIaR) that together enable high-speed acquisition of large-scale synaptic connectivity maps using two-photon holographic optogenetics. Other studies that develop compressive connectivity mapping techniques either use generic compressed sensing algorithms or have only been demonstrated in simulations^8,11–13,23^. Here, however, we show that considerable methodological extensions over these previous algorithms are required to ensure that connectivity is accurately determined in light of the substantial sources of biophysical variability that are intrinsic to real holographic stimulation experiments. We found that use of NWD and CAVIaR provided a mapping speed-up of over an order of magnitude compared to existing approaches in realistic settings, granting the speed and scalability necessary to collect large-scale maps of connectivity within individual experimental sessions.

While in our experiments we typically mapped connectivity from several hundred neurons, this was primarily limited by factors outside of the influence of NWD and CAVIaR, such as the number of neurons targetable by our SLM or the rate at which phase masks could be computed. We expect that the neural population sizes that can practically be mapped within a single experiment should continue to grow with steps to increase the targetable field of view (FOV) (for example, by translating the microscope or SLM stimulation field), with larger commercially available SLMs, or with the use of holographic mesoscopes^41^ granting optical access to potentially many thousands of neurons.

An important direction for future work is the incorporation of simultaneous calcium or voltage imaging into our connectivity mapping experiments^2^. Such an approach should, in principle, resolve exactly which presynaptic candidate neurons are activated on each trial. We expect that this would enable the mapping of larger neural populations and with greater certainty, as fewer trials are required to overcome ambiguities about which neurons elicited presynaptic spikes; however, this approach would not be without its own difficulties: our stimulation regime aims to elicit just a single spike with each pulse, resulting in low-amplitude calcium or voltage transients compared to typical imaging conditions, and synaptic failures could still occur. Additional computational methods would therefore likely be required to overcome these challenges to fully utilize such multimodal data.

Another direction for future research is the modeling and inference of short-term synaptic plasticity under stimulation regimes designed to induce such effects; however, this inference problem could be challenging as it is difficult to precisely resolve amplitude changes in the individual PSCs that summate to give the response to an ensemble stimulus. A more effective strategy in practice would likely be to first perform compressed sensing to identify connected neurons, and then switch to single-target stimulation to probe plasticity-related variables in finer detail. Further, while in this study opsin expression was restricted to specific presynaptic cell types, an important direction is the mapping of both excitatory and inhibitory connections within the same experiment. This could enable the study of the dynamic interplay between excitation and inhibition, believed to play an important role in the function of neural circuits^42^.

For researchers looking to implement compressive connectivity mapping in their own experiments, there are a number of important considerations regarding intrinsic and synaptic plasticity^43^, cell-type specificity, synaptic nonlinearity^44–46^, polysynaptic effects, calibration of the optical system and the use of other computational tools^47,48^ that should be kept in mind (Supplementary Discussion).

**Extended Data Fig. 10:**
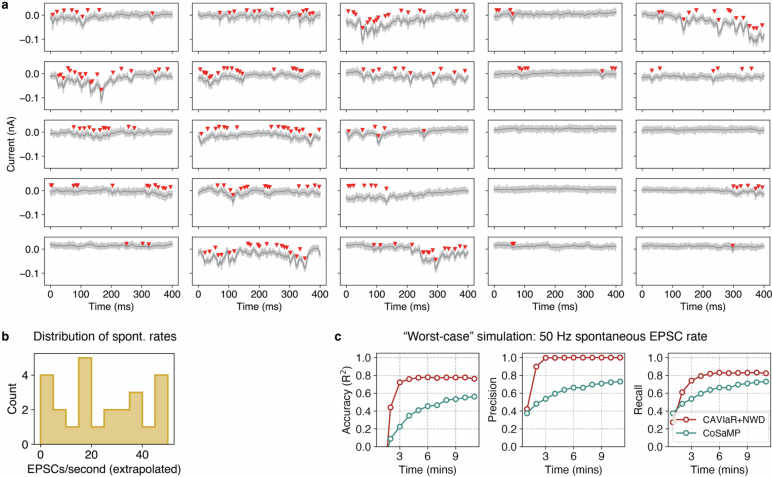
Analysis of CAVIaR performance under in vivo-like conditions. **a**, Detection of spontaneous EPSCs from an in vivo experiment during 25 periods without stimulation. Light gray traces show raw data, dark gray traces are low-pass filtered. Red triangles indicate putative EPSC peaks. **b**, Distribution of EPSC rates across 25 periods of in vivo spontaneous activity. Mean, 23.6 Hz. Max, 50 Hz. EPSC rates extrapolated from 400 ms windows. **c**, Performance of CAVIaR (with NWD) and CoSaMP with a 50 Hz rate of spontaneous PSCs, intended to reflect in vivo noise conditions. N=1000 neurons, 20-target stimulation, 50 Hz stimulation speed, 10% connection density.

Combining NWD and CAVIaR with two-photon holographic optogenetics enables new experiments where extensive maps of connectivity can be obtained with just a few minutes of ensemble stimulation. This could allow for very high-throughput collection and screening of synaptic connectivity maps from brain slices, or for future in vivo applications^14,17,23^ where synaptic connectivity can be directly related to functional activity. Critically, in vivo experiments are impacted by higher background rates of spontaneous activity and more prevalent polysynaptic effects. While further validation must be performed to ensure accuracy in vivo, the impact of each of these impediments is demonstrably reduced by our computational methods (Extended Data Fig. 10 and Supplementary Fig. 8), implying that NWD and CAVIaR may be important tools for precise and scalable connectivity mapping in this experimental regime.

## Methods

### Experimental methods

#### Animals

All experiments on animals were conducted with approval of the Animal Care and Use Committee of the University of California, Berkeley. In all experiments we attempted to use male and female mice equally. Mice used for experiments in this study were transgenic Emx-Cre, PV-Cre or SST-Cre mice obtained by crossing the corresponding lines in-house with a wild-type (CD-1 (ICR) white strain, obtained from The Jackson Laboratory). Mice were housed in cohorts of five or fewer in a reverse 12-h light–dark cycle, with experiments occurring during the dark phase.

#### Opsin expression method

We used a neonatal injection procedure to induce expression of ChroME2s (AAV9.CAG.DIO.ChroME-ST.P2A.H2B-mRuby6) in the visual cortex of Emx-Cre animals or ChroME2f (AAV9.CAG.DIO.Chrome2f.P2A.H2B-mRuby3.WPRE.SV43) in PV-Cre and SST-Cre animals. Both constructs expressed the mRuby3 fluorophore in opsin-positive cell nuclei. Young pups at P3 or P4 were anesthetized by placing them on ice for approximately 3 min. Next, each animal was stabilized under the nanoliter-injector (WPI) and a small portion (30 nl per injection) of virus was injected directly in 3–5 places around V1 via the skin and skull and at 3–5 depths to target L2/3. After the procedure the animal was placed on a heating pad until it recovered. At the end of the procedure the injected litter was returned to their cage with their parents and housed together until reaching approximately 21 days of age.

#### 3D-SHOT holography setup

All experiments were performed using the 3D-SHOT multiphoton holography setup (see ref. ^14^ for details). In brief, the setup was custom built around a commercial Sutter movable object microscope platform (Sutter Instruments) and combined a 3D photostimulation path, a fast resonant galvo two-photon raster scanning imaging path and a wide-field one-photon epifluorescence/IR (infrared) transmitted light imaging path. The stimulation and imaging beams were merged together using a polarizing beamsplitter placed before the microscope tube lens.

A femtosecond fiber laser was used for two-photon photostimulation (Monaco 1035-80-60; 1,040 nm, 1 MHz, 300 fs, Coherent). The stimulation laser was directed onto a blazed diffraction grating (600 l mm^−1^, 1,000 nm blaze, Edmund Optics 49-570) for temporal focusing. To be able to utilize the total available laser power (60 W laser output), the beam was enlarged by a factor of 2.5 to prevent heat damage of the grating surface. The spot on the grating was relayed onto a rotating diffuser where it formed a temporally focused spot. The rotating diffuser was used to both randomize the phase pattern imprinted on the temporally focused spot and to expand the beam in the direction orthogonal to the temporal focusing direction and fully fill the SLM (HSP 1,920 × 1,152 pixels, Meadowlark Optics). The SLM plane was relayed through 4f systems to the back aperture of an Olympus ×20 water immersion objective, resulting in a custom 3D distribution of temporally focused spots at the focus of the objective. Holographic phase masks were calculated using the iterative, in-house written and GPU-optimized Gerchberg–Saxton algorithm^49^ and the intensity distribution was corrected to accommodate for diffraction efficiencies (compensating for possible attenuation effects from the SLM when targeting different regions for stimulation).

The two-photon imaging path relied on a Ti:sapphire laser, Mai Tai (Spectra Physics), with external power control via Pockels cell (Conoptics). For fast raster scanning, the system was equipped with conjugated 8-kHz resonant galvo–galvo systems. The imaging path hardware was controlled by ScanImage software and custom MATLAB code was used to control the SLM for targeted photostimulation and synchronize with imaging.

Epifluorescent one-photon excitation was via a Spectra X (Lumencor) light source filtered by an appropriate excitation filter set. For slice transillumination we used a 750-nm and IR diffuser. The image was collected using an Olympus ×20 magnification water immersion objective and a CCD camera and displayed on a screen enabling targeted patch clamping.

#### Slice electrophysiology

In vitro slice recordings were performed on 300 μm-thick coronal slices coming from 4–6-week-old animals expressing opsin in L2/3 of V1. During slicing the level of opsin expression was checked using a simple laser light to visualize the targeting of the opsin to V1 and the general brightness of the mRuby3 nuclear marker.

Whole-cell patch-clamp protocols were performed in artificial cerebrospinal fluid perfusion solution (119 mM NaCl, 26 mM NaHCO_3_, 20 mM glucose, 2.5 mM KCl, 2.5 mM CaCl, 1.3 mM MgSO_4_ and 1.3 mM NaH_2_PO_4_) in temperature-controlled (33 °C) conditions. Patch pipettes (4–7 M*Ω*) were pulled from borosilicate glass filaments (Sutter Instruments) and filled with a cesium (Cs2^+^)-based internal solution (135 mM CeMeSO_4_, 3 mM NaCl, 10 mM HEPES, 0.3 mM EGTA, 4 mM Mg-ATP, 0.3 mM Na-GTP, 1 mM QX-314, 5 mM TEA-Cl and 295 mM mOsm, pH 7.45) also containing 50 μM Alexa Fluor hydrazide 488 or 594 dye (Thermo Fisher Scientific). For loose-patch recordings the pipettes were filled with standard artificial cerebrospinal fluid. Data were recorded at 20 kHz using a 700b Multiclamp Axon Amplifier (Molecular Devices). The headstage with the electrode holder (G23 Instruments) was controlled by a motorized micromanipulator (MP285A, Sutter Instruments). All data were acquired and analyzed with custom code written in MATLAB using the National Instruments Data Acquisition Toolbox.

#### Learning physiological point spread functions

An opsin-positive cell was loose-patched at various slice depths between 20 and 90 μm and a volume of tissue surrounding that cell was then probed using a dense grid of holograms. The total size of the grid was 65 × 65 × 75 μm, resulting in a 10 × 10 × 7 voxel grid. The interval between the centers of the hologram targets were five pixels (6.5 μm) in the *x*/*y* dimensions and 12.5 μm in the *z* dimension. The phase masks of the grid holograms were pre-computed and stored in memory before performing the measurement. Two versions of the physiological point spread function experiments were performed. One where each hologram was a single spot randomly selected from the grid (*n* = 8 experiments), and another where each hologram contained ten spots: one spot randomly taken from the grid and nine spots (either fixed or randomly placed) outside the grid (*n* = 7 experiments).

Evoked spiking in response to 3–5-ms laser stimulation (30 Hz) across powers between 10 and 60 mW per spot were collected and analyzed using a custom written script in MATLAB. Each trial was randomly repeated 5–7 times. Evoked spiking was calculated by simple thresholding and the mean spiking probability across each grid point and power was calculated for single and ten-spot ensemble experiments. A Gaussian fit to the per-cell normalized mean spiking probability data was applied to estimate the full width at half maximum of the spiking physiological point spread function.

#### Whole-cell targeted mapping experimental protocol

First, a L2/3 opsin-negative cell of pyramidal shape was sealed on (below 40 μm cortical depth, opsin absence judged by fluorophore presence). The tissue was then quickly imaged with approximately 40 frames at 4–5 planes spaced by 25 μm. The cell was always positioned to be in the second plane from the top within the collected stack. The position of the presynaptic candidates were automatically identified by an in-house algorithm detecting round shapes in a specified FOV due to the presence of mRuby3 fluorophore in the opsin-positive cell nuclei.

Next, we computed 20–25 different sets of holograms, where every set of holograms was designed so that every target was stimulated once. Each hologram either targeted an ensemble of presynaptic candidates, or targeted one cell at a time. Further, each set of holograms was organized into sweeps, where each sweep was composed of a baseline period, followed by a single set of holographic stimulation trials, and ended with a short baseline period. A single set of ensemble holograms was obtained by randomly partitioning all targets into ten-target ensembles (without repeating any targets). Additional sets of holograms were obtained by repeatedly performing this random partitioning.

For example, an experiment probing 100 presynaptic candidates would contain data from 45 sweeps of 100 single-target holograms across three powers (15 repetitions per power), and 20 sweeps representing 20 sets of ten-target holograms. Each set of ten-target holograms would consist of 100 targets split into ten different ten-target holograms. This would ultimately result in 200 unique ensemble holograms organized in 20 sweeps. Each sweep would be performed across three powers and repeated three times per power (leading to 180 ensemble sweeps). In total, this would result in 225 sweeps (45 single-target sweeps and 180 ensemble sweeps). Notably, the order of all holograms and powers within each repetition and sweep was randomized.

For pyramidal–pyramidal mapping, the postsynaptic neuron was targeted based on its characteristic pyramidal shape and the absence of an mRuby3 signal in the cell. In an area with high opsin expression it was difficult to identify a completely opsin-negative cell. Thus, for a large portion of putatively opsin-negative cells, stimulating near the electrode still elicited a small direct photocurrent artifact (with a typical amplitude of 50–300 pA). For mapping pyramidal–PV connections, the postsynaptic cell type was confirmed to be PV by observing a fast-spiking response type as a result of a typical current step protocol.

Stable access to each cell was obtained during hologram computation. Then, one-photon photocurrents were measured to assess the overall level of opsin expression before proceeding to two-photon connectivity mapping experiments. Once hologram computation was completed, presynaptic candidate neurons were mapped using randomly interleaved trials containing either single-target holograms or samples from the set of ten-target holograms. Across all trials, single-target holograms were repeated 7–15 times, and each ten-target hologram was repeated up to three times. All stimulations were performed at 30 Hz and at 3–4 different laser powers in both mapping regimes.

Throughout the mapping protocol, the membrane resistance (Rm) and access resistance (Rs) were regularly monitored by applying a series of hyperpolarizing steps every ten trials (corresponding to 20–180 s, depending on the number of presynaptic candidate neurons and the laser power). Sweeps were excluded if the Rs exceeded 35 M*Ω*, and experimental recordings were discarded if less than 40% of the original sweeps were retained. Next, an initial value of the Rs was calculated as the average of the first five Rs measurements logged during connectivity mapping. Then, a rolling average of the Rs over a window of five measurements was applied, and sweeps in which the Rs increased beyond 35% of the initial Rs value were also excluded. If less than 20% of sweeps remained, the recording was discarded. Recordings where zero or one connection(s) were identified were considered as having insufficient opsin expression and excluded from analysis.

Across all datasets, the mean Rs ± s.e. was 20.72 ± 1.52 M*Ω*. By connection type, the mean Rs measurements were 21.27 ± 2.08 M*Ω*, 21.37 ± 3.79 M*Ω*, 24.73 ± 1.61 M*Ω* and 16.47 ± 2.80 M*Ω* for PV–pyramidal, pyramidal–pyramidal, pyramidal–PV and SST–pyramidal, respectively.

#### Power calibration for single and ensemble target

To ensure that the connectivity maps obtained using single-target and ensemble stimulation were comparable, we carefully characterized the power needed to evoke similar photocurrent amplitudes at each presynaptic candidate in both stimulation regimes. To do this, a set of whole-cell experiments was performed in several cell types (pyramidal, PV and SST). In each such experiment, an opsin-positive cell was patched and a direct opsin-photocurrent was recorded while all other experimental parameters were kept the same as in the connectivity mapping experiments. We used a range of powers that were typical for our connectivity mapping protocols. The requested laser power was a multiple of the diffraction efficiency of a given hologram (either single-target or ensemble) and power per cell multiplied by a number of targets in a hologram for a given condition.

In these experiments (Extended Data Fig. 2), we compared the opsin-photocurrent amplitude evoked in response to stimulation of a single target (the patched opsin-positive cell) with the opsin-photocurrent amplitudes evoked by ensemble holograms containing the patched opsin-positive cell. This set of experiments provided a measurement of the difference in the power delivered to the patched cell between these two stimulation regimes. Correcting this difference required ~35% additional power to the ensemble holograms (Extended Data Fig. 2b–d). Applying the correction compensated for the necessary photocurrent and was implemented in all mapping experiments (Extended Data Fig. 2e–g).

#### Pair-patch experimental protocol

The pair-patch mapping protocol was similar to the single-patch protocol, but contained an additional ‘pre-mapping’ step. After establishing stable access to the opsin-negative pyramidal cell, all presynaptic candidates were probed with ten trials of single-power, single-target holograms. A fast online analysis based on the *z*-score provided the coordinates of cells that responded to this simple connection screening procedure. One such identified cell would be approached and loose-patched with a second electrode while monitoring the stability of the postsynaptic recording. The main prerequisite for deciding which cell would be loose-patched (from a handful of putatively connected cells obtained via our simple screening procedure) was the ability to identify the cell in the slice under IR illumination and its accessibility. While the loose patch was established, the surrounding area would be re-imaged to update the positions of the presynaptic candidates. Mapping would then be performed according to the above-described procedure, while recording both postsynaptic responses and presynaptic spiking.

#### Whole-cell grid mapping experimental protocol

Opsin-negative cells (or those expressing minimal amounts of opsin) were whole-cell patched at various slice depths between 30 and 90 μm. Cells were voltage-clamped at a holding potential of −70 mV. After establishing stable access to a cell, we acquired its response to one-photon pulses of increasing duration (1, 3 and 5-ms pulses at 10 Hz). A volume of tissue surrounding that cell would then be probed using a dense grid of holograms across 3–4 powers. The total size of the grid was ~160 × 160 × 100 μm, resulting in a 25 × 25 × 4 voxel grid. The distance between the centers of the hologram targets was five pixels (6.5 μm) in the x/y dimensions and 25 μm in the *z* dimension. The phase masks of the grid holograms were pre-computed and stored in memory before performing the measurement. Recording parameters were frequently checked and logged by pausing the mapping protocol and probing the cell with a series of hyperpolarizing pulses.

#### Statistics and reproducibility

No statistical method was used to predetermine sample sizes, and experiments were generally not anonymized during analysis. Experiments were excluded from analysis if their access resistance exceeded a threshold or changed significantly during the course of the recording, or due to insufficient opsin expression, as described in the mapping protocols in the preceding sections.

### Computational methods

#### Neural waveform demixing network architecture and training

The NWD network is a sequential U-Net^20^ that forms compressed representations of 45-ms single-trial PSC traces via a ‘contraction path’ and generates a PSC waveform at the original trial length but with confounding synaptic currents removed via an ‘expansion path’. Each block in the contraction path consists of a 2× temporal decimation step, a 1D convolution, a batch-norm step, and a rectified linear activation function. Each block in the expansion path consists of a ‘transposed’ 1D convolution (also known as a fractionally strided convolution, or as a pseudo deconvolution), a batch-norm step, a rectified linear activation function and a 2× linear interpolation step. There are four contraction blocks and four expansion blocks, with the outputs of the contraction blocks submitted to the expansion blocks at the corresponding temporal resolution through skip connections as in the original U-Net architecture.

The NWD networks used in this study were trained using 50,000 simulated PSC traces. The simulated PSCs obeyed the following generative structure. First, a template PSC ϱ(⋅ ; *τ*_*r*_, *τ*_*d*_, *Δ*) with parameters *τ*_*r*_, *τ*_*d*_, *Δ* took the form

1 $$\varrho (t;{\tau }_{r},{\tau }_{d},\Delta )=\left[\exp \left(\frac{t-\Delta }{{\tau }_{d}}\right)-\exp \left(\frac{t-\Delta }{{\tau }_{r}}\right)\right]{{\mathbb{1}}}_{[t\ge \Delta ]}.$$

Every simulated PSC *c*(*t*) was the sum of a random number of templates, accounting for the stimulation of multiple connected presynaptic cells,

2 $$c(t)=\mathop{\sum }\limits_{j=1}^{J}\varrho\left(t;{\tau }_{r}^{j},{\tau }_{d}^{j},{\Delta }^{\,j}\right),$$

3 $${\tau }_{X}^{j} \sim \,\text{Uniform}\,\left({\tau }_{X}^{\min },{\tau }_{X}^{\max }\right),$$

4 $${\Delta }^{\,j} \sim \,\text{Uniform}\,\left({\Delta }^{\min },{\Delta }^{\max }\right),$$

5 $$J \sim p(\,J\,),$$

where *X*∈ {*r*, diff} with *τ*_*d*_ = *τ*_*r*_ + *τ*_diff_ to ensure *τ*_*d*_ > *τ*_*r*_, and where *p*(*J*) determined the probability of selecting $J\in {\mathbb{N}}$ (including the possibility of *J* = 0). The PSC *c*_*k*_ for training example *k* was then given as a function of time *t* by

6 $${c}_{k}(t)={c}_{k}^{\,\text{prev}}(t)+{c}_{k}^{{\rm{tar}}}(t)+{c}_{k}^{\text{next}\,}(t)+{g}_{k}(t)+{\epsilon }_{k}(t)$$

where ${c}_{k}^{\,\text{prev}\,}$, ${c}_{k}^{\,\text{tar}\,}$ and ${c}_{k}^{\,\text{next}\,}$ refer to PSCs from the previous trial, the target trial, and the next trial(s), which reproduce the ‘overlapping trials’ effect when stimulating at high speeds. Note that ${c}_{k}^{\,\text{prev}\,}$ is not the same as ${c}_{k-1}^{\,\text{tar}\,}$, as each trial was simulated entirely independently. Here *g*_*k*_(*t*) represents temporally correlated noise from a Gaussian process,

7 $${g}_{k} \sim \,\text{Normal}\,({\boldsymbol{0}},{\bf{K}}),$$

with the covariance matrix **K** defined by the radial basis function kernel with noise variance *σ*_scale_ and characteristic lengthscale *ℓ*_gp_

8 $${{\bf{K}}}_{{t}_{1},{t}_{2}}={\sigma }_{{\rm{scale}}}\exp \left(\frac{-{({t}_{1}-{t}_{2})}^{2}}{2{\ell }_{\,\text{gp}\,}^{2}}\right).$$

Finally, *ϵ*_*k*_(*t*) represents uncorrelated noise sampled from a univariate Gaussian,

9 $${\epsilon }_{k}(t) \sim \,\text{Normal}\left(0,{\sigma }_{\text{noise}\,}^{2}\right)$$

where ${\sigma }_{\,\text{noise}}^{2} \sim U({\sigma }_{{\rm{noise,min}}}^{2},{\sigma }_{\text{noise,max}\,}^{2})$. The NWD network is trained to infer ${c}_{k}^{\,\text{tar}\,}$ from *c*_*k*_ by minimizing the mean-squared error loss.

The time constants of the training data were selected to match the PSCs recorded in experiments via an interactive simulator that overlaid the template waveform on experimental traces. We found that the NWD network performed best when trained separately on training data matched to either excitatory or inhibitory synaptic currents depending on the data being processed. Indeed, because opsin is expressed only in a specific, genetically defined cell type at once, we would not expect stimulation to recruit combinations of monosynaptic currents from multiple different cell types within the same experiment. As the combination of presynaptic and postsynaptic cell types are known ahead of time, in practice we are free to select an NWD network trained to demix synaptic currents of a specific type; however, we note that in future work one could adapt the NWD network to account for multiple cell types simultaneously.

Further, the timescale and variance of the temporally correlated noise *g*_*k*_ can be adjusted to match experimental conditions. For example, in the context of in vivo experiments, it may be critical to account for neuromodulatory signals, which could be approximated by sampling from Gaussian processes with a range of timescales and amplitudes. Similarly, the uncorrelated noise *ϵ*_*k*_ can be tuned to match the electrical noise from the recording electrode.

The NWD network intrinsically performs a denoising of the evoked PSCs as each target PSC in the training data was uncorrupted by the noise terms *g*_*k*_ and *ϵ*_*k*_. We found that we could obtain further improvement in PSC denoising by randomly incorporating 45-ms snippets of pure electrical noise taken from experiments during periods without stimulation. For such ‘negative’ templates, the network was trained to produce an output of zeros.

Finally, we performed a minor correction step to guarantee that the output of the NWD network monotonically decayed after a predefined time^50^, which we found marginally improved accuracy with little computational cost. Namely, after a user-specified time *t*_monotone_, the network output *N*_*t*_ for all *t* > *t*_monotone_ was adjusted by recursively setting ${N}_{t}=\min \{{N}_{t},{N}_{t-1}\}$.

We implemented NWD using PyTorch Lightning. Networks were trained using stochastic gradient descent with a batch size of 64 and learning rate of 0.01 for 3,000–6,000 epochs, which was sufficiently long for the optimizer to converge in our training regime.

#### Statistical model

CAVIaR aims to fit a statistical model to the demixed optogenetic data that relates patterns of holographic stimulation to the resulting measurements of postsynaptic current. Let ${{\bf{c}}}_{k}\in {{\mathbb{R}}}^{T}$ represent the PSC trace on trial *k*. In accordance with the trial structure used for NWD, we assume that *T* = 900 frames (or 45 ms), with photostimulation beginning at *t* = 100 (or 5 ms). To make use of compressed sensing-style techniques, we collapse the demixed, denoised PSC trace into a single number by integrating over the duration of the trial,

10 $${y}_{k}=\mathop{\int}\nolimits_{0}^{T}{c}_{k}^{\,\text{NWD}\,}(t)dt,\quad k=1,\ldots ,K,$$

where ${c}_{k}^{\,\text{NWD}\,}$ represents the demixed trace on trial *k*. The measurements *y*_*k*_ therefore represent the total synaptic charge transfer resulting from the transmission of presynaptic spikes. Assuming the postsynaptic neuron is held under voltage clamp and that space clamp imperfections do not induce substantial nonlinearities, we may model these measurements as a simple sum of optogenetically evoked presynaptic spikes weighted by the strengths of the corresponding synapses,

11 $${y}_{k} \sim \,\text{Normal}\,\left({{\bf{w}}}^{\top }{{\bf{s}}}_{:,k},{\sigma }^{2}\right),\qquad {\sigma }^{-2} \sim \,\text{Gamma}\,\left({t}_{{\rm{sh}}},{t}_{{\rm{ra}}}\right).$$

Here ${\bf{w}}\in {{\mathbb{R}}}^{N}$ is a vector of synaptic weights encoding the charge transfer resulting from a single spike for each neuron *n* = 1, …*N*. Because opsin is expressed in a specific presynaptic cell type (in this study exactly one of SST, PV or pyramidal), there is no risk of potentially confounding ‘cancellation’ effects where both excitatory and inhibitory PSCs are elicited and sum to zero.

Next, **s**_:,*k*_ is the vector of presynaptic spikes on trial *k*. Note that, as described below, spike generation is highly stochastic, and hence *s*_*nk*_ is a latent variable. The synaptic weights are regularized by imposing a Gaussian prior,

12 $${w}_{n} \sim \,\text{Normal}\,(u,{b}^{2}),$$

with hyperparameters *u* and *b*^2^, and the variance *σ*^2^ is intended to account for electrical noise from the recording electrode and variability in the amplitude of the evoked PSCs. Model parameters are listed in Table 1.

**Table 1:** Table of model parameters and their interpretations

Symbol	Interpretation	Hyperparameters	Posterior
**w**	Connection strengths	*u*, *b*^2^	***μ***, ***Ω***
*s*_*nk*_	Presynaptic spike	–	*λ*_*nk*_
${\phi }_{n}^{0},\,{\phi }_{n}^{1}$	Presynaptic sigmoid coefficients	**v**, **L**	***ν***_*n*_, ***Σ***_*n*_
*σ*^2^	Observation noise variance	*t*_sh_, *t*_ra_	*θ*_sh_, *θ*_ra_
*z*_*k*_	Spontaneous synaptic current		
*y*_*k*_	Observed response (integral of the PSC)		
*I*_*nk*_	Laser power applied to neuron *n* on trial *k*		
*N* (*n*)	Number of neurons (neuron index)		
*K* (*k*)	Number of trials (trial index)		
*f*	Sigmoid function		

The variability of spike generation in our experimental data shows that the probability of generating a spike is not only different between cells, but also across power levels, and is therefore effectively impossible to know ahead of time. We propose a structured presynaptic model that can flexibly adjust to varying spike probabilities on a cell-by-cell basis. In particular, presynaptic spikes are assumed to be driven by holographic photostimulation according to a linear–nonlinear-Bernoulli model,

13 $${s}_{nk}| {{\boldsymbol{\phi }}}_{n} \sim \,\text{Bernoulli}\,\left(\;f\left({\phi }_{n}^{0}{I}_{nk}-{\phi }_{n}^{1}\right)\right)$$

where $f(x)=1/(1+\exp (-x))$ is the sigmoid nonlinearity, *I*_*nk*_ denotes the power of the stimulation laser focused on cell *n* on trial *k*, and the sigmoid coefficients follow bivariate normal distributions

14 $${{\boldsymbol{\phi }}}_{n} \sim \,\text{Normal}\,({{\bf{v}}}_{n},{{\bf{L}}}_{n}).$$

During inference we thus effectively perform a Bayesian logistic regression of each cell’s inferred presynaptic spikes on the laser targets. This presynaptic model allows spike probabilities to adapt to variation in opsin expression via the sigmoid coefficient term ${\phi }_{n}^{0}$, as well as to variation in rheobase via the intercept term ${\phi }_{n}^{1}$.

#### Posterior inference

**Variational approximation**. The goal of CAVIaR is to infer the posterior distribution over the hidden variables, including the synaptic weights and presynaptic spikes. The posterior describes the distribution of parameter values that are consistent with the observed data, given our prior assumptions. Notably, this provides a description of uncertainty in the inferred synaptic weights. It is not computationally feasible to compute the exact posterior distribution, but standard methods have been developed to approximate the posterior^51^. Here we modify the conventional coordinate-ascent variational inference algorithm by augmenting it with several steps that account for spontaneous synaptic currents and biophysical plausibility of the inferred spikes. To this end, we note that the posterior distribution over the latent variables factorizes as

15 $$\begin{array}{r}p({\bf{w}},{\bf{s}},{\boldsymbol{\phi }},{\sigma }^{2}\;|\;{\bf{y}},{\mathcal{I}})\propto p({\sigma }^{2})\mathop{\prod }\limits_{n=1}^{N}p({w}_{n})p({{\boldsymbol{\phi }}}_{n})\mathop{\prod }\limits_{k=1}^{K}p({s}_{nk}\;|\;{I}_{nk},{{\boldsymbol{\phi }}}_{n})p({\;y}_{k}\;|\;{\bf{w}},{{\bf{s}}}_{:,k},{\sigma }^{2}).\end{array}$$

We approximate the posterior distribution $p({\mathcal{Z}}| {\bf{y}},{\mathcal{I}})$ by a variational model $q({\mathcal{Z}})$ (where ${\mathcal{Z}}$ represents the set of latent variables) that obeys a similar factorization,

16 $$q({\bf{w}},{\bf{s}},{\boldsymbol{\phi}},{\sigma }^{2})=q({\sigma}^{2}\;|\;{\theta}_{{\rm{sh}}},{\theta}_{{\rm{ra}}})q({\bf{w}}\;|\;{\boldsymbol{\mu}},{\boldsymbol{\Omega}})\mathop{\prod}\limits_{n=1}^{N}q({{\boldsymbol{\phi}}}_{n}| {{\boldsymbol{\nu}}}_{n},\mathop{\boldsymbol{\Sigma}}\nolimits_{n})\mathop{\prod}\limits_{k=1}^{K}q({s}_{nk}\;|\;{\lambda}_{nk})$$

where the individual factors are *q*(**w**∣***μ***, ***Ω***) = Normal(**w**∣***μ***, ***Ω***), *q*(*s*_*nk*_∣*λ*_*nk*_) = Bernoulli(*s*_*nk*_∣*λ*_*nk*_), *q*(***ϕ***_*n*_∣***ν***_*n*_, ***Σ***_***n***_) = Normal(***ϕ***_*n*_∣***ν***_*n*_, ***Σ***_*n*_) and *q*(*σ*^−2^∣*θ*_sh_, *θ*_ra_) = Gamma(*σ*^−2^∣*θ*_sh_, *θ*_ra_).

Given the above approximation, coordinate-ascent variational inference seeks to perform an update of each factor $q({{\mathcal{Z}}}_{i})$ one by one for all ${{\mathcal{Z}}}_{i}\in {\mathcal{Z}}$, with the idea being that each update moves the approximate posterior $q({\mathcal{Z}})$ closer to the true posterior $p({\mathcal{Z}}| {\bf{y}},{\mathcal{I}})$ in the sense of the Kullback–Leibler (KL) divergence^51^. We take a variety of approaches to updating each factor depending on the relative tractability of the update. The complete algorithm is given in algorithm 1 (Supplementary Information).

**Inference of synaptic weights**. First, it can be shown that the optimal variational update for the synaptic strengths **w**, conditional on all other latent variables ${\mathcal{Z}}\setminus {\bf{w}}$, obeys the equation

17 $$\begin{array}{r}q({\bf{w}}\;|\;{\boldsymbol{\mu }},{\boldsymbol{\Omega }})\propto \exp {{\mathbb{E}}}_{q({\mathcal{Z}}\setminus {\bf{w}})}\left[\ln p({\bf{y}},{\mathcal{Z}}\;|\;{\mathcal{I}})\right],\end{array}$$

see, for example,^52^. As both the prior on **w** and the observations are Gaussian-distributed, one can evaluate equation (17) analytically and solve for ***μ*** and ***Ω*** by ‘completing the square’, yielding the block update

18 $${\boldsymbol{\Omega }}={\left(\frac{{\theta }_{{\rm{sh}}}}{{\theta }_{{\rm{ra}}}}\mathop{\sum }\limits_{k = 1}^{K}({{\bf{D}}}_{k}+{{\bf{M}}}_{k})+\frac{1}{{b}^{2}}{{\bf{I}}}_{n}\right)}^{-1},\,{\boldsymbol{\mu }}={\boldsymbol{\Omega }}\left(\frac{{\theta }_{{\rm{sh}}}}{{\theta }_{{\rm{ra}}}}\mathop{\sum }\limits_{k=1}^{K}{y}_{k}{{\boldsymbol{\lambda }}}_{:,k}+\frac{1}{{b}^{2}}u\right)$$

where ${{\bf{D}}}_{k}=\,\text{diag}\,\left({\lambda }_{1,k}(1-{\lambda }_{1,k}),\ldots ,{\lambda }_{Nk}(1-{\lambda }_{Nk})\right)\in {{\mathbb{R}}}^{N\times N}$ is diagonal and ${{\bf{M}}}_{k}={{\boldsymbol{\lambda }}}_{:,k}{{\boldsymbol{\lambda }}}_{:,k}^{\top }\in {{\mathbb{R}}}^{N\times N}$. Inspecting the update for ***μ***, one sees that a neuron’s synaptic weight is only determined by the magnitude of the postsynaptic responses on trials for which that neuron actually elicited a spike. However, those spikes are not directly observed, and must themselves be inferred from the data.

**Inference of presynaptic spikes**. Given a measurement *y*_*k*_ resulting from the photostimulus *I*_:,*k*_ and our current estimates of synaptic strengths, electrical noise, and a priori presynaptic spike probabilities, we must decide which of the targeted cells actually elicited a spike. In early trials, this depends more on the prior (the presynaptic model) rather than on the likelihood, and this balance shifts in favor of the likelihood the more experimental data that is collected. However, unlike in equation (17), the optimal variational update for the presynaptic spike probabilities *λ*_*nk*_ cannot be solved for analytically. Instead, one can recognize (see, for example, ref. ^52^) that the KL divergence from the approximate to the true posterior, KL(*q*∥*p*), can be decomposed into the sum

19 $$\,\text{KL}\,(q\parallel p)=\ln p({\bf{y}})-{\mathcal{L}}({\mathcal{Z}})$$

where $\ln p({\bf{y}})=\ln \int\,p({\bf{y}},{\mathcal{Z}})d{\mathcal{Z}}$ is the logarithmic model evidence and where

20 $${\mathcal{L}}({\mathcal{Z}})={{\mathbb{E}}}_{q}\left[\ln p({\bf{y}},{\bf{w}},{\bf{s}},{\boldsymbol{\phi }},{\sigma }^{2})\right]+{{\mathcal{H}}}_{q}\left[{\bf{w}},{\bf{s}},{\boldsymbol{\phi }},{\sigma }^{2}\right],$$

with ${{\mathcal{H}}}_{q}[x]$ the Shannon entropy of variable *x* under density *q*. As the KL divergence is always non-negative, the inequality ${\mathcal{L}}({\mathcal{Z}})\le \ln p({\bf{y}})$ always holds, and therefore ${\mathcal{L}}({\mathcal{Z}})$ is a lower bound on the logarithmic model evidence (referred to as the ‘evidence lower bound’). By maximizing the evidence lower bound as a function of the variational model parameters, ${\mathcal{L}}({\mathcal{Z}})$ approaches $\ln p({\bf{y}})$, and hence drives KL(*q*∥*p*) toward zero. If the bound is tight, that is, if ${\mathcal{L}}({\mathcal{Z}})=\ln p({\bf{y}})$, the KL divergence is zero and the variational approximation to the posterior becomes exact. To this end, differentiating ${\mathcal{L}}$ and solving for *λ*_*nk*_ yields the update

$$\begin{array}{l}\ln \left(\frac{{\lambda }_{nk}}{1-{\lambda }_{nk}}\right)=\,{{\mathbb{E}}}_{q({\boldsymbol{\phi }})}\left[\ln \left(\frac{{f}_{nk}}{1-{f}_{nk}}\right)\right]\\\qquad\qquad\;\;-\frac{{\theta}_{{\rm{sh}}}}{2{\theta }_{{\rm{ra}}}}\left\{-2{y}_{k}{\mu }_{n}+2{\mu }_{n}\mathop{\sum}\limits_{j\ne n}{\mu }_{j}{\lambda }_{jk}-\left({\mu }_{n}^{2}+{\beta }_{n}^{2}\right)\right\}\end{array}$$

where we have defined ${f}_{nk}=f\left({\phi }_{n}^{0}{I}_{nk}-{\phi }_{n}^{1}\right)$. The expectation in the above term is analytically intractable due to multiple nonlinearities. We thus make a Monte Carlo approximation to the expectation

21 $${{\mathbb{E}}}_{q({\boldsymbol{\phi }})}\left[\ln \left(\frac{{f}_{nk}}{1-{f}_{nk}}\right)\right]\approx \frac{1}{M}\mathop{\sum }\limits_{m=1}^{M}\ln \left(\frac{{f}_{nk}[m]}{1-{f}_{nk}[m]}\right)$$

with $${f}_{nk}[m]=f\left({\phi }_{n}^{0}[m]{I}_{nk}-{\phi }_{n}^{1}[m]\right)$$, and for each pair $${({\phi}_{n}^{0}[m],{\phi }_{n}^{1}[m])}^{\top}\sim$$$$q({{\boldsymbol{\phi }}}_{n}| {{\boldsymbol{\nu }}}_{n},{{\boldsymbol{\Sigma }}}_{n})$$. As discussed below, the sigmoid coefficients are sampled from a truncated multivariate normal distribution; however, sampling from truncated multivariate normal distributions is computationally demanding and remains an open area of research^53^. Instead, the terms $${\phi }_{n}^{0}$$ and $${\phi }_{n}^{1}$$ are sampled independently from their truncated marginal distributions.

Inspecting the updates for *λ*_*nk*_, one notices that each update depends on all other neurons in the population, but is independent of all other trials. Thus, we update the spike probabilities for all trials simultaneously, but perform this one neuron at a time (where the order of the neuron updates is randomized every iteration). Immediately following the inference of a neuron’s spikes, we evaluate their biophysical plausibility as a function of laser power. Patch-clamp studies with ChroME2 opsins show that the probability of eliciting an action potential increase monotonically with laser power^16^. We therefore perform an isotonic regression through the inferred spike rate using the pool adjacent violators algorithm (PAVA)^54^.

Concretely, let ${K}_{n}^{p}=\{k\in \{1,\ldots ,K\;\}:{I}_{nk}=p\}$ be the set of all trials on which neuron *n* was stimulated with power *p*, and let

22 $${\bar{\lambda }}_{n}^{p}=\frac{1}{| {K}_{n}^{\,p}| }\sum _{k\in {K}_{n}^{p}}{\lambda }_{nk}$$

denote the average inferred spike probability for neuron *n* at power *p*. PAVA computes an isotonic regression function ${\hat{F}}_{n}$ by solving the optimization problem

23 $$\,\text{minimize}\,\sum _{p\in {\mathcal{P}}}{\left({\bar{\lambda }}_{n}^{p}-{\hat{F}}_{n}(p)\right)}^{2}\,\,\text{such that}\,\,{\hat{F}}_{n}\,\,\text{is non-decreasing}\,,$$

where ${\mathcal{P}}$ is the set of non-zero powers used in the experiment. This provides us with an isotonically constrained optogenetic ‘power curve’ ${\hat{F}}_{n}$ that we use to judge whether or not the putative synapse connecting neuron *n* to the patched neuron is sufficiently plausible. In particular, we require that at the maximal power used in the experiment the inferred probability of successfully initiating and transmitting a spike is greater than some threshold *θ*_PAVA_ ∈ [0, 1], which we refer to as the ‘minimum spike rate at maximum power’; however, some fraction of the inferred spikes may result from spontaneous synaptic currents, which can lead to false positives if left unaccounted for. We therefore infer the rate of spontaneous currents (*λ*_spont_, described below) and add this to the user-defined threshold *θ*_PAVA_ to adaptively adjust the plausibility criterion. Neurons with ${\hat{F}}_{n}(\mathop{\max }\limits_{k}{I}_{nk}) < {\theta }_{{\rm{PAVA}}}+{\lambda }_{{\rm{spont}}}$ (whose adjusted maximal spike rate is below *θ*_PAVA_) are considered unconnected and have their synaptic weights and inferred spike probabilities set to zero. We used *θ*_PAVA_ = 0.4 for mapping inhibitory presynaptic neurons and *θ*_PAVA_ = 0.3 for mapping excitatory presynaptic neurons.

Note that the likelihood of spurious connections arising from spontaneous PSCs only at the highest laser power is vanishingly small (Supplementary Note 1).

We also use a ‘masking’ procedure to prevent small, noise-driven postsynaptic measurements from causing spurious spike inferences. To do this, we evaluate a test statistic *τ* on each trace *c*_*k*_. If $\tau ({c}_{k}) < {\tau }_{\min }$ we force *λ*_*nk*_ = 0 for all *n* = 1, …, *N*. The statistic *τ* is typically chosen to be the sample autocorrelation.

**Inference of presynaptic sigmoid coefficients**. The presynaptic sigmoid coefficients ***ϕ*** (governing the presynaptic models) are updated using Laplace’s method. Namely, rather than solving for the optimal variational approximation or optimizing the evidence lower bound, we learn a posterior distribution over each ***ϕ***_*n*_ by making a second-order Taylor approximation about the posterior mode, with the posterior covariance matrix given by the inverse of the Hessian matrix appearing in the Taylor series. In some cases, we encountered pathological spike inference when the sigmoid coefficients ***ϕ***_*n*_ became negative, and hence we enforce a positivity constraint in the mode-finding algorithm described below. Moreover, the Monte Carlo approximation described in equation (21) depends closely on the non-negativity of the sampled variates. Thus, we truncate the Laplace approximation to the non-negative real line,

24 $$q({{\boldsymbol{\phi }}}_{n}\;|\;{{\boldsymbol{\nu }}}_{n},\mathop{\boldsymbol{\Sigma}}\nolimits_{n})=\,\text{TruncNorm}\,({{\boldsymbol{\phi }}}_{n}\;|\;{{\boldsymbol{\nu }}}_{n},\mathop{\boldsymbol{\Sigma}}\nolimits_{n})$$

with ${\nu }_{n}^{i} > 0$ for *i* ∈ {0, 1} and $\,\text{supp}\,\,q({{\boldsymbol{\phi}}}_{n}\;|\;{{\boldsymbol{\nu }}}_{n},{{\boldsymbol{\Sigma}}}_{n})={{\mathbb{R}}}_{\ > \ 0}^{2}$.

Note that, while PAVA is a nonparametric estimator of the spike rate, its use differs from the presynaptic spike model which takes a parametric form. The presynaptic model is used to evaluate the prior probability of eliciting a spike, whereas PAVA is used to evaluate the plausibility of the inferred spikes.

We use Newton’s method with a log barrier and backtracking line search to identify the posterior mode ***ν***_*n*_. To this end, define the objective function with barrier penalty *t* as

$${{\Psi}}_{n}^{t}=-{{\mathbb{E}}}_{q({{\bf{s}}}_{n})}\left[\mathop{\sum}\limits_{k=1}^{K}\ln p({s}_{nk}\;|\;{{\boldsymbol{\phi }}}_{n},{I}_{nk})+\ln p({{\boldsymbol{\phi }}}_{n}\;|\;{\bf{v}},{\bf{L}})\right]-\frac{1}{t}\mathop{\sum }\limits_{i=0}^{1}\ln ({\phi }_{n}^{i}),$$

where we average over the spike uncertainty. Note that the presynaptic models depend only on the spikes **s**, and are independent conditional on **s**, allowing us to compute Laplace approximations in parallel across cells.

The mode-finding algorithm starts with an initial *t*. We then solve for the mode by iterating the Newton steps

25 $${{\boldsymbol{\nu }}}_{n}^{(t)}\leftarrow {{\boldsymbol{\nu }}}_{n}^{(t)}-\kappa {{\bf{H}}}_{nt}^{-1}{{\bf{J}}}_{nt}$$

until convergence, where **J** and **H** are respectively the Jacobian and Hessian of the presynaptic model for neuron *n* with barrier sharpness *t*

26 $${{\bf{J}}}_{nt}={\nabla}_{{{\boldsymbol{\phi }}}_{n}}{{\Psi}}_{n}^{t},\quad {{\bf{H}}}_{nt}=\nabla {\nabla }_{{{\boldsymbol{\phi }}}_{n}}{{\Psi}}_{n}^{t}.$$

The stepsize *κ* is adaptively selected using a standard backtracking line search rule. We then increase *t* to sharpen the log barrier and repeat as required. Typically we only sharpen *t* two or three times as this proved sufficient for our data.

**Inference of observation noise variance**. To infer the observation noise variance, we use the same approach as with the update rule for the synaptic weights (equation (17)). In particular, noting that

$$q({\sigma }^{-2}\;|\;{\theta}_{{\rm{sh}}},{\theta}_{{\rm{ra}}})\propto \exp {{\mathbb{E}}}_{q({\mathcal{Z}}\setminus {\sigma }^{-2})}\left[\ln p({\bf{y}},{\mathcal{Z}}\;|\;{\mathcal{I}})\right],$$

and recognizing that the gamma prior is conjugate to the Gaussian likelihood, one obtains the variational update

$${\theta }_{{\rm{sh}}}={t}_{{\rm{sh}}}+\frac{K}{2},\quad {\theta }_{{\rm{ra}}}={t}_{{\rm{ra}}}+\frac{1}{2}\mathop{\sum }\limits_{k=1}^{K}{{\mathbb{E}}}_{q}\left[{\left({y}_{k}-{{\bf{w}}}^{\top }{{\bf{s}}}_{:,k}\right)}^{2}\right]$$

where the expectation above can be evaluated analytically, though we leave it in the above form for legibility.

**Inference of spontaneous synaptic currents**. Finally, we estimate spontaneous synaptic currents *z*_*k*_ using soft-thresholding. The idea is that, given a numerical tolerance *ϵ* (where typically *ϵ* = 0.05) and positively rectified residuals

27 $${e}_{k}:= {[{\;y}_{k}-{{\boldsymbol{\mu}}}^{\top }{{\boldsymbol{\lambda }}}_{:,k}]}_{+}$$

we apply a soft-thresholding function *S* with penalty *γ* defined by

28 $$S(e,\gamma):=\left\{\begin{array}{ll}0\quad &\,\text{if}\,e\le \gamma\\e-\gamma\quad&\,\text{otherwise.}\,\end{array}\right.$$

We then iteratively shrink the penalty *γ* until the norm of the residual data comprises no more than *ϵ* of the norm of the observed data; that is, until

29 $$\frac{\mathop{\sum }\nolimits_{k = 1}^{K}{({\;y}_{k}-{{\boldsymbol{\mu }}}^{\top }{{\boldsymbol{\Lambda }}}_{:,k}-{z}_{k})}^{2}}{\mathop{\sum }\nolimits_{k = 1}^{K}{y}_{k}^{2}}\le \epsilon$$

where *z*_*k*_ is obtained via the soft-thresholded residuals and where ***Λ*** is the *N* *× K* matrix of inferred spikes. We also require that (*z*_1_, …, *z*_*K*_) be approximately orthogonal to ***λ***_*n*_ for all *n*, and apply the masking procedure as noted in the spike inference step above, leading to the spontaneous synaptic current estimator

30 $${z}_{k}=\left\{\begin{array}{ll}0\quad &\,\text{if}\mathop{\sum}\limits_{n=1}^{N}{\lambda }_{nk} > {\theta }_{{\rm{orthog}}}\;\text{or}\;\tau({c}_{k})<{\tau}_{\min}\\ S({e}_{k},\gamma)\quad &\,\text{otherwise}\,\end{array}\right.$$

where *θ*_orthog_ ≈ 0, but is not exactly zero to allow for numerical imprecision. The spontaneous events are constrained to be approximately orthogonal to the inferred spikes to prevent variability in PSC amplitude causing false positives.

#### Post hoc scan for false negatives

Occasionally the CAVIaR algorithm will erroneously declare a neuron to be disconnected, either due to a failure to meet the PAVA-based plausibility criterion in the earliest iterations of the algorithm, or due to the biological data violating the assumptions of the underlying statistical model. To correct for this, following inference we perform a post hoc scan for potential false negatives. The idea is to reconnect neurons that CAVIaR originally declared to be disconnected by checking whether spontaneous PSCs coinciding with the stimulation of a selected neuron could constitute valid postsynaptic responses when the rigidity of the statistical model is relaxed. As the overwhelming bulk of connections are already identified by the first CAVIaR pass, missed connections are comparatively rare and thus we found a simple greedy algorithm to be effective.

The false-negative scanning algorithm is given in algorithm 3 (Supplementary Information). Briefly, the algorithm begins by collecting all neurons *S*_disc_ declared to be disconnected by the first CAVIaR pass. It then selects the neuron n^*^ ∈ *S*_disc_ with the greatest number of coincidental spontaneous PSCs, and checks if assigning these PSCs to neuron n^*^ would satisfy the PAVA criterion. If so, neuron n^*^ is declared connected, with the posterior distribution of the reconnected neuron’s parameters determined by sample statistics of the corresponding spontaneous PSCs. Neuron n^*^ is then removed from *S*_disc_ (whether reconnected or not), and the algorithm repeats until ${S}_{{\rm{disc}}}={{\emptyset}}$.

#### Inference of canonical postsynaptic current waveforms

Once we have inferred the presynaptic spike matrix ***Λ***, we can obtain accurate PSC waveforms ${{\bf{r}}}_{n}\in {{\mathbb{R}}}^{T}$ using ridge regression. Collecting the waveforms in the rows of a matrix **R**, they can be obtained simultaneously by solving the non-negative *L*_2_ problem

31 $$\hat{{\bf{R}}}=\mathop{{\rm{argmin}}}\limits_{{\bf{R}}\ge {\boldsymbol{0}}}\left\{\parallel \!\!{\bf{C}}-{{\boldsymbol{\Lambda }}}^{\top }{\bf{R}}{\parallel }_{F}+\gamma \parallel \!\!{\bf{R}}{\parallel }_{F}\right\}$$

where ${\bf{C}}\in {{\mathbb{R}}}^{K\times T}$ is the matrix of PSC traces (**c**_1_, …, **c**_*K*_), *γ* > 0 is the ridge penalty, and ∥ ⋅ ∥_*F*_ is the Frobenius norm. Note that by using the spike matrix ***Λ*** instead of the optogenetic ‘design matrix’ (where each element of the matrix determines which neurons were merely stimulated as in ref. ^8^, rather than which spiked as a result of photostimulation), the estimated waveforms are much less biased by trials in which neurons were not photoactivated. We used this ridge regression approach to determine synaptic weights from PSCs throughout the paper.

#### Comparing estimated connectivity vectors

To quantify the similarity between connectivity vectors obtained using single-target stimulation and ensemble stimulation, we used the coefficient of determination (*R*^2^, computed using the scikit-learn Python package) as well as the precision and recall, defined as

32 $$\,\text{Precision}=\frac{\text{true positives}}{\text{true positives}+\text{false positives}\,},$$

33 $$\,\text{Recall}=\frac{\text{true positives}}{\text{true positives}+\text{false negatives}\,}.$$

Intuitively, the precision represents the fraction of connections found by compressed sensing (CAVIaR) that are ‘true’ and the recall represents the fraction of ‘true’ connections that are correctly found by compressed sensing. As we do not have access to the ground-truth connectivity in real experiments, we treat the connections identified using single-target stimulation as the target for compressed sensing.

#### Leave-one-hologram-out cross-validation

We assess the accuracy of the CAVIaR inferences using leave-one-hologram-out cross-validation (LOHO-CV; algorithm 4 in Supplementary Information). Let ${\mathcal{H}}$ represent the complete set of hologram designs; that is, $h\in {\mathcal{H}}$ determines which neurons will be targeted for stimulation, regardless of laser power. LOHO-CV proceeds by selecting $h\in {\mathcal{H}}$, fitting CAVIaR to the PSC traces and stimuli corresponding to all other holograms ${\mathcal{H}}\setminus \{h\}$, and then averaging samples from the posterior predictive distribution over responses to hologram *h* to obtain an estimate of the postsynaptic response (at each power level).

### Simulated circuit mapping experiments

We used simulated data to characterize the performance of the techniques we tested. To ensure the accuracy of this characterization, rather than sampling directly from the generative model that we propose, we added several layers of biophysical realism (further physiological detail could be included by using parameters from the NeuroElectro database^33^). In particular, we sampled noisy synaptic currents that were first demixed using NWD before being supplied as input to the connectivity inference algorithms. This way we could test for the combined accuracy of NWD and connectivity inference.

The simulated data was generated as follows. First, sigmoid parameters ${\phi }_{n}^{X}$ were sampled uniformly from $U({\phi }_{\min }^{X},{\phi }_{\max }^{X})$ for *X* ∈ {0, 1}. Then, presynaptic spikes *s*_*nk*_ were sampled from a linear–nonlinear-Bernoulli model

34 $${s}_{nk} \sim \,\text{Bernoulli}\,\left(\sigma \left({\phi }_{n}^{0}{I}_{nk}-{\phi }_{n}^{1}\right)\right)$$

where the laser power on each trial was randomly selected from a discrete set matched to the experimental data. Each neuron had a canonical PSC transient $h(t,{\Delta }_{nk};{\tau }_{r}^{n},{\tau }_{d}^{n})$ that took a form similar to that used for training the NWD networks. The unnormalized transient ${\tilde{h}}_{n}$ was defined by

35 $$\tilde{h}\left(t,{\Delta}_{nk};{\tau}_{r}^{n},{\tau}_{d}^{n}\right)=\left[\exp \left(-\frac{t-{\Delta }_{nk}}{{\tau }_{d}^{n}}\right)-\exp \left(-\frac{t-{\Delta }_{nk}}{{\tau }_{r}^{n}}\right)\right]{{\mathbb{1}}}_{[t\ge {\Delta }_{nk}]}$$

where ${\tau }_{r}^{n}$ and ${\tau }_{d}^{n}$ are rise and decay time constants and *Δ*_*nk*_ represents the spike delay (the combined spike activation and transmission latencies) for neuron *n* on trial *k*. The transient was then normalized to take integral 1,

36 $$h\left(t,{\Delta}_{nk};{\tau }_{r}^{n},{\tau }_{d}^{n}\right)=\tilde{h}\left(t,{\Delta }_{nk};{\tau }_{r}^{n},{\tau }_{d}^{n}\right)/\int\,{\tilde{h}}_{n}\left({t}^{{\prime} },{\Delta }_{nk};{\tau }_{r}^{n},{\tau }_{d}^{n}\right)d{t}^{{\prime} },$$

such that the synaptic weight for that neuron (*w*_*n*_, defined as the synaptic charge transfer below) was preserved when multiplying the weight by the PSC.

The spike delays were laser power-dependent so that, in accordance with experimental data for the opsins used in this study^16^, neurons initiated and propagated spikes faster if stimulated at higher powers. Concretely, for laser power *I*_*nk*_ targeted at neuron *n* on trial *k*, the spike time was sampled from a right-shifted gamma distribution,

37 $${\Delta }_{nk} \sim \,\text{Gamma}\,\left(\frac{\alpha }{{I}_{nk}^{2}},\beta ,{\Delta }_{\min }\right)$$

where

38 $$\,\text{Gamma}\,(x\;|\;a,b,t)=\frac{{b}^{a}}{{\rm{\Gamma }}(a)}{(x-t)}^{a-1}\exp (-b(x-t)){{\mathbb{1}}}_{[x\ > \ t]}.$$

Note that as the mean of the shifted gamma distribution is *t* + *a*/*b*, the optogenetically evoked spikes followed (in expectation) an inverse-square dependence of time on power, consistent with the physics of two-photon absorption^25^:

39 $${\mathbb{E}}[{\Delta }_{nk}]={\Delta }_{\min }+\frac{\alpha }{\beta {I}_{nk}^{2}}.$$

The synaptic weights *w*_*n*_ are intended to model the total synaptic charge transfer resulting from the transmission of a presynaptic spike to the postsynaptic neuron. We sampled the weights in a way that reflects the typical observations from our experiments. Namely, of the neurons that were chosen to be synaptically connected, a small number of them were strongly connected (in our simulations this was 20% of the connected population, though the precise value did not notably impact our results) and a large number of them were more weakly connected (80%). Specifically, for a given connectivity rate *α* ∈ (0, 1), a subset of ⌈*αN*⌉ neurons were randomly selected as being connected to the postsynaptic neuron. Then, if a neuron *n* was chosen to be strongly connected, its weights were sampled as

40 $${w}_{n}\sim\,U\left({w}_{\min}^{\,\text{strong}},{w}_{\max}^{\text{strong}\,}\right)$$

where ${w}_{\min }^{\,\text{strong}\,}$ and ${w}_{\max }^{\,\text{strong}\,}$ respectively represent the lower and upper bounds of the uniform distribution. If *n* was weakly connected then its weights were sampled as

41 $${w}_{n}\sim\,\text{Exp}\left({w}_{{\rm{mean}}}^{{\rm{weak}}},{w}_{\min}^{\text{weak}\,}\right)$$

where the exponential distribution above is in its two-parameter, right-shifted form

42 $$\,\text{Exp}\,(x\;|\;\lambda ,\Delta )=\frac{1}{\lambda }\exp \left(-\frac{1}{\lambda }(x-\Delta )\right){{\mathbb{1}}}_{[x\ge \Delta ]}$$

with *Δ*≥0.

We used two approaches to generating PSC traces. Either we directly simulated individual 45-ms trials for each *k* = 1, …, *K*, or we simulated continuous circuit mapping experiments at 20-kHz sampling resolution (matched to the experimental data) that lasted for tens of minutes. Using the latter approach, we could very closely mimic the exact contribution of confounding synaptic currents arising from stimulation at very high frequencies. Simulations performed in this continuous manner were subsequently restructured into the usual 45-ms snippets of activity.

In the first ‘trial-wise’ approach to simulating data, the presynaptic spikes, synaptic weights, and PSC kernels were used to generate the postsynaptic responses *y*_*k*_ as

43 $${y}_{k}=\int\left({w}_{n}{m}_{nk}{s}_{nk}{h}_{n}(t)+{h}_{k}^{\,\text{spont}\,}(t)+{g}_{k}(t)+{\epsilon }_{k}(t)\right)dt.$$

Here *m*_*nk*_ is a multiplicative noise term, ${h}_{k}^{\,\text{spont}\,}(t)$ is a spontaneous PSC term, *g*_*k*_(*t*) is a temporally correlated noise term, and *ϵ*_*k*_(*t*) is an additive noise term.

The multiplicative noise term *m*_*nk*_ accounted for the fact that, in our experimental data, the precise amplitudes of PSC transients were observed to vary from trial to trial. We sampled *m*_*nk*_ from a log-normal distribution,

44 $${m}_{nk}\sim\,\text{LogNormal}\left(0,{\sigma}_{\text{mult}\,}^{2}\right),$$

such that the median postsynaptic charge transfer following a presynaptic spike still took the value *w*_*n*_. The spontaneous term ${h}_{k}^{\,\text{spont}\,}$ was either a PSC as in equation (36) with random time constants, amplitudes, and spike times, or the zero vector, depending on the probability of spontaneous events. Temporally correlated noise, ${{\bf{g}}}_{k}\in {{\mathbb{R}}}^{T}$, was sampled from a Gaussian process,

45 $${{\bf{g}}}_{k} \sim \,\text{Normal}\,({\boldsymbol{0}},{\bf{K}})$$

where **K** was defined by the radial basis function kernel,

46 $${{\bf{K}}}_{{t}_{1},{t}_{2}}={\sigma }_{{\rm{scale}}}\exp \left(\frac{-{({t}_{1}-{t}_{2})}^{2}}{2{\ell }_{\,\text{gp}\,}^{2}}\right)$$

for *t*_1_, *t*_2_ = 1, …, *T*. Finally, the additive noise *ϵ*_*k*_(*t*) was sampled independently and identically from a zero-mean Gaussian, $${\epsilon}_{k}(t)\sim$$$$\text{Normal}(0,{\sigma}_{\text{noise}\,}^{2})$$. Due to high computational tractability this trial-wise approach was used to generate the heatmaps in Fig. 3.

The second ‘continuous experiment’ approach to simulation was much more computationally demanding. Simulating a 30-min experiment at 20 KHz requires 36,000,000 time points, making, for example, the use of Gaussian processes prohibitive. Our approach was to generate vectors encoding spike times for each neuron and convolve this with the corresponding neuron’s PSC kernel while batching over blocks of time. To this end, an experiment of length *T* was evenly subdivided into trials according to the stimulation frequency *f*. For each such trial, we sampled spikes, spike latencies, and multiplicative noise for each neuron as described above. Then, using this collection of variables, we defined spike vectors ${{\boldsymbol{\zeta }}}_{n}\in {{\mathbb{R}}}^{T}$ for *n* = 1, …, *N* by setting

47 $${\zeta }_{n}(\lceil {t}_{k}+{\Delta }_{nk}\rceil )={w}_{n}{m}_{nk}{s}_{nk},\quad \,\text{for each}\,k=1,\ldots ,K,$$

where *t*_*k*_ is the timebin at which the *k*th trial begins, and *ζ*_*n*_(*t*) = 0 at all other timebins. Similarly, we generated spontaneous spike vectors ${\{{{\boldsymbol{\zeta }}}_{j}^{\,\text{spont}\,}\}}_{j = 1}^{J}$ at a specified rate *λ*_spont_ (in Hz), where each ${{\boldsymbol{\zeta }}}_{j}^{\,\text{spont}\,}$ encodes the activation of a single spontaneous PSC with waveforms ${{\bf{h}}}_{j}^{\,\text{spont}\,}$ defined by randomly sampled time constants, amplitudes, and onset times. The full-length postsynaptic measurement vector was then obtained by convolving the spike vectors with the PSC waveforms and summing across neurons and spontaneous inputs,

48 $${\bf{c}}=\mathop{\sum }\limits_{n=1}^{N}{{\bf{h}}}_{n}* {{\boldsymbol{\zeta }}}_{n}+\mathop{\sum }\limits_{j=1}^{J}{{\bf{h}}}_{j}^{\,\text{spont}\,}* {{\boldsymbol{\zeta }}}_{j}^{\,\text{spont}\,}+{\epsilon}.$$

Here the noise follows a first-order autoregressive process

49 $$\epsilon(t)\sim\,\text{Normal}\left(\gamma \epsilon (t-1),{\sigma}_{\text{noise}\,}^{2}\right)$$

with autoregressive coefficient *γ* ∈ (0, 1), which was suitably scalable for continuous simulated experiments.

The postsynaptic responses *y*_*k*_ used by the connectivity inference algorithms were extracted from **c** by setting

50 $${{\bf{c}}}_{k}=(c({t}_{k}-{t}_{{\rm{pre}}}),c({t}_{k}-{t}_{{\rm{pre}}}+1),\ldots ,c({t}_{k}+{t}_{{\rm{post}}})),$$

51 $${y}_{k}=\int\,{c}_{k}^{\,\text{NWD}\,}(t)dt,$$

where *t*_*k*_ represents the beginning of the *k*th trial, *t*_pre_ = 100 and *t*_post_ = 800, and ${{\bf{c}}}_{k}^{\,\text{NWD}\,}$ represents the trace **c**_*k*_ after demixing. Note that as the rate of stimulation increases, the interstimulus interval decreases, and the trial windows increasingly overlap in time. This leads to a confounding of the observed PSCs as in Fig. 2, which the NWD network must suppress.

### Combinatorics of expected interstimulus intervals

Let *N* represent the total number of potential presynaptic neurons, *R* the size of the ensemble and *f* the rate of stimulation (in Hz). Assuming neurons are chosen uniformly at random, the probability of selecting neuron *n* ∈ {1, …, *N*} is

52 $$\frac{\left(\begin{array}{c}N-1\\ R-1\end{array}\right)}{\left(\begin{array}{c}N\\ R\end{array}\right)}=\frac{R}{N}.$$

Hence, one must stimulate *N*/*R* times on average to return to the same neuron. If stimulation occurs at *f* Hz, this takes *N*/*Rf* seconds.

### Reporting summary

Further information on research design is available in the Nature Portfolio Reporting Summary linked to this article.

## Online content

Any methods, additional references, Nature Portfolio reporting summaries, source data, extended data, supplementary information, acknowledgements, peer review information; details of author contributions and competing interests; and statements of data and code availability are available at 10.1038/s41593-025-02053-7.

## Supplementary information

Supplementary Information Supplementary Discussion, Algorithms 1–4, Tables 1–3, Figs. 1–15, Notes 1–3 and References.

Reporting Summary

## Data Availability

The experimental data for this manuscript are available via figshare at https://figshare.com/s/1315937c4ce1c2552262 (ref. ^55^).

## Code availability

CAVIaR is implemented in Python using JAX^56^, can be run on GPU and is freely available at https://github.com/marcustriplett/circuitmap together with a PyTorch Lightning implementation of NWD.
