# Supplementary material for: Rapid learning of neural circuitry from holographic ensemble stimulation enabled by model-based compressed sensing
Source: Nat Neurosci. 2025 Sep 17;28(10):2154–65. doi: 10.1038/s41593-025-02053-7 (PMC12497653; doi:10.1038/s41593-025-02053-7)
Supplement: Supplementary file 1 — Supplementary Discussion, Algorithms 1–4, Tables 1–3, Figs. 1–15, Notes 1–3 and References. [file 41593_2025_2053_MOESM1_ESM.pdf]

nature neuroscience

Supplementary information https://doi.org/10.1038/s41593-025-02053-7

Rapid learning of neural circuitry from holographic ensemble stimulation enabled by model-based compressed sensing

In the format provided by the authors and unedited

Rapid learning of neural circuitry from holographic ensemble stimulation enabled by model-based compressed sensing

Marcus A. Triplett[1,2,3,4,†,*], Marta Gajowa[5,*], Benjamin Antin[1,2,3,4], Masato Sadahiro[5], Hillel Adesnik[5,†], and Liam Paninski[1,2,3,4]

[1]Mortimer B. Zuckerman Mind Brain Behavior Institute, Columbia University, NY
[2]Grossman Center for the Statistics of Mind, Columbia University, NY
[3]Center for Theoretical Neuroscience, Columbia University, NY
[4]Department of Statistics, Columbia University, NY
[5]Department of Molecular and Cell Biology, University of California, Berkeley, CA
† Correspondence: marcus.triplett@columbia.edu, hadesnik@berkeley.edu
* These authors contributed equally

Supplementary Information

Supplementary Discussion

Using model-based compressed sensing in practice

To implement and validate our computational methods in real mapping experiments we used cutting-edge optogenetic techniques that offered several key advantages. Namely, since the ChroME2f opsin is potent with fast off-kinetics, we could titrate the pulse width and range of stimulation laser powers such that with high probability stimulation generated either 0 or 1 presynaptic spike(s), with minimal instances of more than one spike (Extended Data Fig. 1), facilitating tractable presynaptic spike inference. By comparison, potent opsins with slow off-kinetics (such as ChRmine [1]) elicit larger and less predictable presynaptic spike counts [2] that could make precisely inferring synaptic weights more computationally challenging. Finally, an important assumption of CAVIaR is that, everything else held equal, the probability of eliciting a spike increases monotonically with laser power (on average). While the ChroME2 opsins used in this study exhibit such behavior, some opsins have been shown to change the probability of spiking a neuron non-monotonically under certain laser powers (described in ref. [2]). It is therefore critical that an appropriate opsin or range of powers is determined during a calibration session prior to mapping using CAVIaR, such that monotonicity is upheld and that no more than one spike is elicited by stimulation.

We also relied on the fact that ChroME2f expression is targeted to the soma through a fusion with the Kv2.1 tag [2, 3], reducing the likelihood of triggering spikes in untargeted neurons by off-target stimulation of nearby neurites [4, 5]. Moreover, we used two-photon holography to illuminate entire somas at once [6–8] and randomly switched the stimulation laser between three or more powers, effectively probing for presynaptic neurons at multiple excitation levels. Stimulating with low power further reduces the risk of off-target stimulation, but may fail to elicit spikes in neurons with low opsin expression or high rheobases. Notably, it cannot in general be known ahead of time whether a lack of optogenetically-evoked PSCs was due to the stimulated neuron not being connected or because of a failure to elicit spikes, an ambiguity that can only be resolved through high power stimulation. On the other hand, stimulating with high power increases the risk of off-target stimulation but elicits spikes with high probability. We therefore elected to use a randomized design that probed neurons at multiple power levels, but that still minimized the risk of off-target stimulation (Extended Data Fig. 1).

A further point of consideration is that if both single-target and ensemble stimulation cause off-target activation of a connected neuron, the probed target could be erroneously classified as a true positive connection even if the target

itself is not connected. While our steps to improve the precision of our stimulation system minimize this effect, off-target stimulation could still occasionally occur [9], which should be kept in mind when interpreting connectivity maps obtained using two-photon optogenetics. Computational approaches to optimizing stimulation sites and laser power could help to further reduce or eliminate off-target activation [10].

In our validation experiments we mapped the same populations of neurons using both single-target and ensemble stimulation. In practice, we would not expect an experimenter to perform both kinds of stimulation within each experiment, as this would greatly inflate the required experiment time and defeat the purpose of our approach. Instead, to determine whether mapping results are accurate when only having access to ensemble stimulation, we recommend using LOHO-CV – this enables calibration of the accuracy of the inferred connections without the need to reference single-target stimulation. If the LOHO-CV R^2 is poor (due to e.g. deteriorating recording quality or a potential miscalibration in the optical system), the associated experiments could be excluded from further analysis.

What is the right number of neurons to stimulate per hologram to maximize accuracy and throughput using compressed sensing? Our simulations indicated that, generally, stimulating 2-5% of the total population size per trial leads to robust and fast convergence across both smaller (N=250 neurons) and larger (N=1000) population sizes for a range of connection densities (Supplementary Fig. 11). In rarer cases where connection density is expected to approach or exceed 0.5, one would reduce the stimulated ensemble size (eventually approaching single-target stimulation, though performed quickly using NWD) as compressed sensing methods are unlikely to achieve high performance in this regime (also see ref. [11] for related analysis). Alternatively, experimenters could consider stimulation strategies that balance the distribution of targets in space: distant neurons tend to have a substantially reduced connection density [12], and so by stimulating ensembles consisting of targets that are both distant and nearby the effective connection density remains low, facilitating the use of compressive connectivity mapping even in densely connected networks.

Our results indicate that NWD and CAVIaR can substantially reduce the cumulative time spent stimulating tissue to map connectivity. However, the speed of a mapping experiment can also be impacted by other factors, including the time required to identify and segment neurons, monitor the access resistance of the patch clamp, and compute hologram phase masks. Each of these factors similarly affects mapping performed using single-target stimulation and ensemble stimulation. While acquiring an image stack to identify presynaptic candidates is required for all experiments using targeted stimulation, measuring access resistance poses an additional trade-off between the speed of a mapping experiment and the desire to make regular quality checks. An ideal experimental design would therefore proceed with the longest periods of uninterrupted stimulation possible to yield the maximal benefits of NWD and CAVIaR, but with brief ($\sim$200 ms) automatic pauses included to check access resistance. The attending experimenter could then be prompted to address inadequate access only when necessary. In the case of phase mask generation, efficient computation is critical since otherwise experiment time could be dominated by this step. We expect that high-speed phase mask estimation methods, such as those based on deep learning [13, 14], could play an increasingly important role in rapid connectivity mapping.

Concerns and limitations

A potential concern with our proposed connectivity mapping approach is that it may engage intrinsic or short-term plasticity, given our suggested high speed of stimulation and the fact that entire ensembles of neurons are stimulated at once. To help calibrate mapping protocols, we derived a simple combinatorial expression for the expected interstimulus interval (ISI, in seconds) for any given neuron (Methods). Assuming connectivity from N total neurons is mapped by stimulating a random ensemble of size R on each trial at a speed of f Hz, the mean ISI is $\frac{N}{Rf}$. Thus, a typical experiment mapping $\sim$300 neurons with 10-target stimulation at 30 Hz yields an expected stimulation return time of 1 second. We expect that the risk of engaging intrinsic plasticity (resulting from repeated depolarization of presynaptic candidate neurons) at this ISI is low, as prior work has shown that depolarizing ChroME2-expressing neurons at rates even up to 40 Hz only minimally induces intrinsic plasticity [2]. Further, direct analysis of our data did not suggest recruitment of intrinsic plasticity (Supplementary Note 2). However, short-term plasticity (i.e. synaptic facilitation or depression) can operate over a much broader range of timescales [15]. While short-term plasticity did not appear to impact our ability to use compressed sensing to map PV-pyramidal connectivity, it could have played a role in other cell-type combinations (cf. further discussion on

cell types below), and could have contributed to the trial-to-trial variability we observed in PSCs (e.g. Fig. 4a, b).

An additional concern associated with ensemble stimulation is the recruitment of potential polysynaptic effects arising due to recurrent connectivity in the cortex [16]. However, in slice experiments we do not expect this to be a major factor influencing the quality of our inferences compared to *in vivo* conditions. Further, as CAVIaR is designed to model monosynaptic connections only, we could use LOHO-CV to assess whether polysynaptic effects were confounding our analysis. This was possible because CAVIaR would not be able to capture the variance generated by polysynaptic pathways. While LOHO-CV therefore suggested that polysynaptic effects did not compromise our compressed sensing approach (Fig. 5b,c), the presence of polysynaptic effects likely depends on brain region and number of simultaneously illuminated targets, and definitively determining whether polysynaptic effects occur would likely require a different recording modality such as voltage imaging.

In order to validate our compressed sensing approach, we mapped each neural population using both single-target and ensemble stimulation and examined how closely the resulting connectivity maps aligned. This approach relies on earlier work validating single-target stimulation-based connectivity mapping using the traditional approach of paired recordings [17, 18]. As such, it should be kept in mind that different choices of opsins and stimulation techniques between our study and in earlier work could affect how closely our optogenetic connectivity maps reflect paired recordings. Nevertheless, our calibration experiments suggest that our optical resolution is comparable to prior work, and our ability to precisely evoke single spikes is considerably improved due to combined advances in scanless two-photon holography and opsin engineering [2, 8], suggesting that our connectivity mapping results should not deviate markedly from paired recordings compared to prior research.

A critical challenge in validating compressed sensing approaches using single-target stimulation is ensuring that the power delivered to target neurons is consistent between single-target stimulation and ensemble stimulation. Even if an optical system has been calibrated, there can still be small yet unavoidable differences in the amount of two-photon excitation ultimately delivered to neurons (Extended Data Fig. 2). These subtle differences in power can sometimes evoke spikes in single-target stimulation differently to ensemble stimulation, leading to differences in which neurons are identified as being presynaptic. While our results almost always show high precision (maximum precision in this study, 0.95 for PV-pyramidal experiments; minimum, 0.76 for pyramidal-pyramidal experiments), the recall is almost always lower (maximum recall, 0.84 for PV-pyramidal; minimum, 0.67 for pyramidal-PV). This suggests that although the connections found by compressed sensing are almost always replicated by single-target connectivity mapping, disagreements between the two approaches primarily stem from putative connections being missed by compressed sensing.

What could explain this discrepancy? Our LOHO-CV analysis shows that for holograms targeting novel combinations of neurons (i.e. groups of neurons that have never been stimulated together before), we achieve high predictive performance (average R^2 across 14 PV-E experiments, 0.71). If CAVIaR were simply missing connections it should have found, this would be reflected in the distribution of LOHO-CV R^2 values, which did not appear to be the case in our data (Fig. 5c). A possible explanation for the discrepancy between single-target and compressed sensing approaches under some experimental conditions is therefore that there is a small decrease in the amount of laser power ultimately delivered to some target neurons when performing ensemble stimulation compared to single-target stimulation, despite our best efforts to equalize this in calibration experiments. The extent to which such "power gaps" have been addressed during calibration should therefore be kept in mind when interpreting the precision and recall of compressed sensing methods.

Performance differences between cell types

Our experiments revealed that CAVIaR and NWD achieved a range of performance levels depending on cell type, including a particularly high level of precision (0.95) when mapping PV-pyramidal connections compared to pyramidal-pyramidal connections (0.76). This difference could be due to the lower PSC amplitudes typically associated with pyramidal-pyramidal synapses, which can make them more difficult to detect and differentiate from background noise. Additionally, direct photocurrent artifacts may occasionally interfere with signal interpretation in experiments where both presynaptic and postsynaptic neurons are opsin-expressing [19], despite the suppressive effect of NWD. However, due to the extremely high number of pyramidal neurons in cortex and their sparse connectivity, the need for high-throughput probing of large numbers of potential connections within individual experiments is crucial. Thus, the significantly increased

throughput enabled by model-based compressed sensing remains a critical advantage, even with reduced precision in this experimental regime.

Compressive approaches to connectivity mapping rely on linear postsynaptic integration of presynaptic inputs [20]. Thus, nonlinearities arising from space-clamp imperfections [21] and/or dendritic integration [22, 23] could impact the performance of any compressed sensing method. While we have taken steps to mitigate space-clamp errors by monitoring access resistance during our electrophysiological recordings, we cannot rule out the possibility that remaining nonlinearities could also underlie performance differences between the cell type combinations used in this study.

Algorithm 1: Coordinate-ascent variational inference and isotonic regularization (CAVIaR)

input: PSC traces $\mathbf{c}$, stimulus information $\mathcal{I}$, PAVA threshold θ_{PAVA}, spontaneous penalty backtracking scalar α, soft orthogonality threshold θ_{orthog}, minimal test statistic τ_{min}, number of iterations *iters*

initialise $\lambda_{nk} \leftarrow 1$ for all n, k such that $I_{nk} > 0$ and $\tau_{\text{test}}(\mathbf{c}_k) \geq \tau_{\text{min}}$
$\lambda_{\text{spont}} \leftarrow 0$ // initialize spontaneous rate to 0
$i \leftarrow 1$
while $i \leq iters$ **do**
  update $q(\mathbf{w} \mid \boldsymbol{\mu}, \boldsymbol{\Omega}) \propto \exp \mathbb{E}_{q(\mathcal{Z} \setminus \mathbf{w})}\left[\ln p(\mathbf{y}, \mathcal{Z} \mid \mathcal{I})\right]$ // variational solution for synaptic weights
  for $n = 1, \ldots, N$ **do** // infer spikes via Monte Carlo ELBO solution
    sample $\boldsymbol{\phi}_n[m] \sim q(\boldsymbol{\phi}_n \mid \boldsymbol{\nu}, \boldsymbol{\Sigma})$ for $m = 1, \ldots, M$
    for $k = 1, \ldots, K$ **do**
      $\lambda_{nk} \leftarrow \text{argmax}_{\lambda_{nk}}$ ELBO$(\lambda_{nk} \mid \{\boldsymbol{\phi}_n[m]\}_{m=1}^{M})$
    end
    $\hat{F}_n \leftarrow$ PAVA$(\mathcal{I}, \boldsymbol{\lambda}_n)$ // estimate optogenetic power curve
    if $\hat{F}_n(\max_k I_{nk}) < \theta_{\textit{PAVA}} + \lambda_{\textit{spont}}$ **then** // check plausibility criterion
      $\mu_n \leftarrow 0$, $\boldsymbol{\lambda}_n \leftarrow \mathbf{0}$
    end
  end
  for $n = 1, \ldots, N$ **do** // Laplace approx of receptive field posterior
    $\boldsymbol{\nu}_n, \boldsymbol{\Sigma}_n \leftarrow$ RECEPTIVEFIELDLAPLACE$(\mathbf{s}_n, \boldsymbol{\phi}_n, \mathbf{v}, \mathbf{L})$
  end
  update $q(\sigma^{-2} \mid \theta_{\text{sh}}, \theta_{\text{ra}}) \propto \exp \mathbb{E}_{q(\mathcal{Z} \setminus \sigma^{-2})}\left[\ln p(\mathbf{y}, \mathcal{Z} \mid \mathcal{I})\right]$ // variational solution for noise precision
  while $\|\mathbf{y} - \boldsymbol{\mu}^{\top}\boldsymbol{\Lambda} - \mathbf{z}\|_2^2 / \|\mathbf{y}\|_2^2 \geq \epsilon$ **do** // begin spontaneous PSC inference
    for $k = 1, \ldots, K$ **do**
      if $\sum_{n=1}^{N} \lambda_{nk} \leq \theta_{\textit{orthog}}$ *and* $\tau_{\textit{test}}(\mathbf{c}_k) \geq \tau_{\textit{min}}$ **then**
        $z_k \leftarrow S([y_k - \boldsymbol{\mu}^{\top}\boldsymbol{\lambda}_{:,k}]_+, \gamma)$ // soft-threshold residual with penalty γ
      end
    end
    $\gamma \leftarrow \alpha\gamma$ // shrink penalty
  end
  $\lambda_{\text{spont}} \leftarrow \frac{1}{K} \sum_{k=1}^{K} \mathbb{1}_{[z_k \neq 0]}$ // update spontaneous rate
  $i \leftarrow i + 1$
end
$\boldsymbol{\mu}, \boldsymbol{\Omega}, \boldsymbol{\Lambda}, \boldsymbol{\phi}, \mathbf{z} \leftarrow$ FNSCAN$(\boldsymbol{\mu}, \mathbf{z})$ // scan resulting spontaneous PSCs for potential false negatives

Algorithm 2: RECEPTIVEFIELDLAPLACE

for $n = 1, \dots, N$ **do**
  for $t = 1, \dots, T_{max}$ **do**
    $\kappa \leftarrow 1$ // Newton stepsize
    $\Psi_n(\boldsymbol{\phi}_n) = -\mathbb{E}_{q(s_n \mid \boldsymbol{\lambda}_n)} \left[\sum_{k=1}^{K} \ln p(\mathbf{s}_{nk} \mid \boldsymbol{\phi}_n, I_{nk}) + \ln p(\boldsymbol{\phi}_n \mid \mathbf{v}, \mathbf{L}) \right] - \frac{1}{\alpha_{\text{barrier}}} \sum_{i=0}^{1} \ln(\phi_n^i)$
    $\mathbf{J} = \nabla_{\boldsymbol{\phi}_n} \Psi_n$, $\mathbf{H} = \nabla\nabla_{\boldsymbol{\phi}_n} \Psi_n$
    $\mathbf{d} = \mathbf{H}_n^{-1} \mathbf{J}_n$ // search direction
    while $\Psi_n(\boldsymbol{\phi}_n + \kappa \mathbf{d}) > \Psi_n(\boldsymbol{\phi}_n) + \alpha_{backtrack}\, \kappa\, \mathbf{J}_n^{\top} \mathbf{d}$ **do** // backtrack
      $\kappa \leftarrow \beta_{\text{backtrack}}\, \kappa$
    end
    $\boldsymbol{\phi}_n \leftarrow \boldsymbol{\phi}_n - \kappa \mathbf{d}$ // make step
  end
  $\boldsymbol{\nu}_n \leftarrow \boldsymbol{\phi}_n$
  $\boldsymbol{\Sigma}_n \leftarrow \mathbf{H}_n^{-1}$
end
$q(\boldsymbol{\phi} \mid \boldsymbol{\nu}_n, \boldsymbol{\Sigma}_n) =$ TruncNormal$(\boldsymbol{\nu}_n, \boldsymbol{\Sigma}, 0, \infty)$ // truncate support to $(0, \infty)$

Algorithm 3: FNSCAN

input: Synaptic weights $\boldsymbol{\mu}$, spontaneous synaptic currents $\mathbf{z}$, stimulus information $\mathcal{I}$, PAVA threshold θ_{PAVA}
$S_{\text{disc}} \leftarrow \{n \in \{1, \dots, N\} : \mu_n = 0\}$ // initialize pool of candidate neurons
while $|S_{disc}| > 0$ **do**
  for $n = 1, \dots, N$ **do** // collect spontaneous PSC indices aligning with neuron stim times
    spont$_n \leftarrow \{k \in \{1, \dots, K\} : z_k \neq 0$ and $I_{nk} > 0\}$
  end
  $n^* \leftarrow \text{argmax}_n\, |\text{spont}_n|$ // select neuron with most coincidental spontaneous PSCs
  $\hat{F}_{n^*}^{\text{spont}} \leftarrow$ PAVA $\left((I_{n^*k})_{k \in \text{spont}_n}, (z_k)_{k \in \text{spont}_n} \right)$ // estimate putative power curve
  if $\hat{F}_{n^*}^{spont}(\max_k I_{n^*k}) \geq \theta_{PAVA}$ **then**
    $\mu_{n^*} \leftarrow$ mean$(\{z_k : k \in \text{spont}_{n^*}\})$ // neuron passes PAVA criterion, declare connected
    $\beta_{n^*} \leftarrow$ s.e.m.$(\{z_k : k \in \text{spont}_{n^*}\})$
    for $k \in spont_{n^*}$ **do**
      $\lambda_{n^*k} \leftarrow 1$ // declare spontaneous PSC to be spike from neuron n^*
      $z_k \leftarrow 0$ // remove spontaneous PSC from vector $\mathbf{z}$
    end
  end
  $S_{\text{disc}} \leftarrow S_{\text{disc}} \setminus \{n^*\}$ // remove n^* from pool of disconnected neurons
end

Algorithm 4: Leave-one-hologram-out cross-validation (LOHO-CV)

input: Demixed PSCs $\mathbf{c}$, holographic stimulation patterns $\mathcal{H}$, number of posterior samples J

for $h \in \mathcal{H}$ **do**
  estimate variational parameters $\boldsymbol{\mu}, \boldsymbol{\beta}, \boldsymbol{\nu}, \boldsymbol{\Sigma}$ from $\{(\mathbf{c}_k, I_{:,k}) : I_{:,k}$ is not an instance of $h\}$ with CAVIaR
  for $j = 1, \ldots, J$ **do**
    for $n = 1, \ldots, N$ **do** // begin ancestral sampling from posterior predictive distribution
      sample $(\phi_n^0, \phi_n^1) \sim$ TruncNorm$(\boldsymbol{\nu}_n, \boldsymbol{\Sigma}_n)$
      sample $w_n \sim$ Normal$(\mu_n, \beta_n^2)\mathbb{1}_{[\mu_n \neq 0]} + \delta_0 \mathbb{1}_{[\mu_n = 0]}$
      for *each laser power* p **do**
        sample $s_n^p \sim$ Bernoulli$(\sigma(\phi_n^0 h_n^p - \phi_n^1))$, where h_n^p is the power delivered to neuron n assuming hologram h is applied with power p
      end
    end
    for *each laser power* p **do**
      set $y^p[j] \leftarrow \mathbf{w}^\top \mathbf{s}^p$
    end
  end
  predict response to hologram h at power p as $\frac{1}{J}\sum_{j=1}^{J} y^p[j]$
end

Symbol	Interpretation	Value
τ_r^{min}	Rise time constant minimum	10
τ_r^{max}	Rise time constant maximum	40
$\tau_{\text{diff}}^{\text{min}}$	Difference between rise and decay time constants minimum	150
$\tau_{\text{diff}}^{\text{max}}$	Difference between rise and decay time constants maximum	340
$\Delta_{\text{tar}}^{\text{min}}$	PSC onset time minimum (target trial)	160
$\Delta_{\text{tar}}^{\text{max}}$	PSC onset time maximum (target trial)	400
$\Delta_{\text{prev}}^{\text{min}}$	PSC onset time minimum (previous trial effects)	-400
$\Delta_{\text{prev}}^{\text{max}}$	PSC onset time maximum (previous trial effects)	159
$\Delta_{\text{next}}^{\text{min}}$	PSC onset time minimum (next trial effects)	400
$\Delta_{\text{next}}^{\text{max}}$	PSC onset time maximum (next trial effects)	899
σ_{scale}	GP variance	0.045
ℓ_{gp}	GP lengthscale	45
$\sigma^2_{\text{noise,min}}$	Noise variance minimum	0.001
$\sigma^2_{\text{noise,max}}$	Noise variance maximum	0.02

Supplementary Table 1: Parameters used for training an NWD network for inhibitory-to-excitatory mapping experiments. Assumes timesteps measured at 20 kHz.

Symbol	Interpretation	Value
τ_r^{min}	Rise time constant minimum	10
τ_r^{max}	Rise time constant maximum	40
$\tau_{\text{diff}}^{\text{min}}$	Difference between rise and decay time constants minimum	60
$\tau_{\text{diff}}^{\text{max}}$	Difference between rise and decay time constants maximum	120
$\Delta_{\text{tar}}^{\text{min}}$	PSC onset time minimum (target trial)	160
$\Delta_{\text{tar}}^{\text{max}}$	PSC onset time maximum (target trial)	400
$\Delta_{\text{prev}}^{\text{min}}$	PSC onset time minimum (previous trial effects)	-400
$\Delta_{\text{prev}}^{\text{max}}$	PSC onset time maximum (previous trial effects)	159
$\Delta_{\text{next}}^{\text{min}}$	PSC onset time minimum (next trial effects)	400
$\Delta_{\text{next}}^{\text{max}}$	PSC onset time maximum (next trial effects)	899
σ_{scale}	GP variance	0.045
ℓ_{gp}	GP lengthscale	45
$\sigma^2_{\text{noise,min}}$	Noise variance minimum	0.001
$\sigma^2_{\text{noise,max}}$	Noise variance maximum	0.02

Supplementary Table 2: Parameters used for training an NWD network for excitatory-to-excitatory mapping experiments. Assumes timesteps measured at 20 kHz.

Symbol	Interpretation	Default value
ϕ^0_{min}	Presynaptic sigmoid coefficient (0) minimum	0.2
ϕ^0_{max}	Presynaptic sigmoid coefficient (0) maximum	0.25
ϕ^1_{min}	Presynaptic sigmoid coefficient (1) minimum	10
ϕ^1_{max}	Presynaptic sigmoid coefficient (1) maximum	15
$\tau_{r,\text{min}}$	Rise time constant minimum	10
$\tau_{r,\text{max}}$	Rise time constant maximum	40
$\tau_{\Delta,\text{min}}$	Difference between rise and decay time constants minimum	250
$\tau_{\Delta,\text{max}}$	Difference between rise and decay time constants maximum	300
α	Rate parameter for gamma-distributed spike times	10^4
β	Shape parameter for gamma-distributed spike times	15
Δ_{min}	Minimum spike time	60
$w^{\text{strong}}_{\text{min}}$	Strong synaptic weight minimum	20
$w^{\text{strong}}_{\text{max}}$	Strong synaptic weight maximum	40
$w^{\text{weak}}_{\text{mean}}$	Weak synaptic weight mean (of the unshifted distribution)	4
$w^{\text{weak}}_{\text{min}}$	Weak synaptic weight minimum	5
ℓ_{gp}	GP lengthscale	50
σ_{scale}	GP noise variance	4^{-3}

Supplementary Table 3: Table of default parameters for simulation studies.

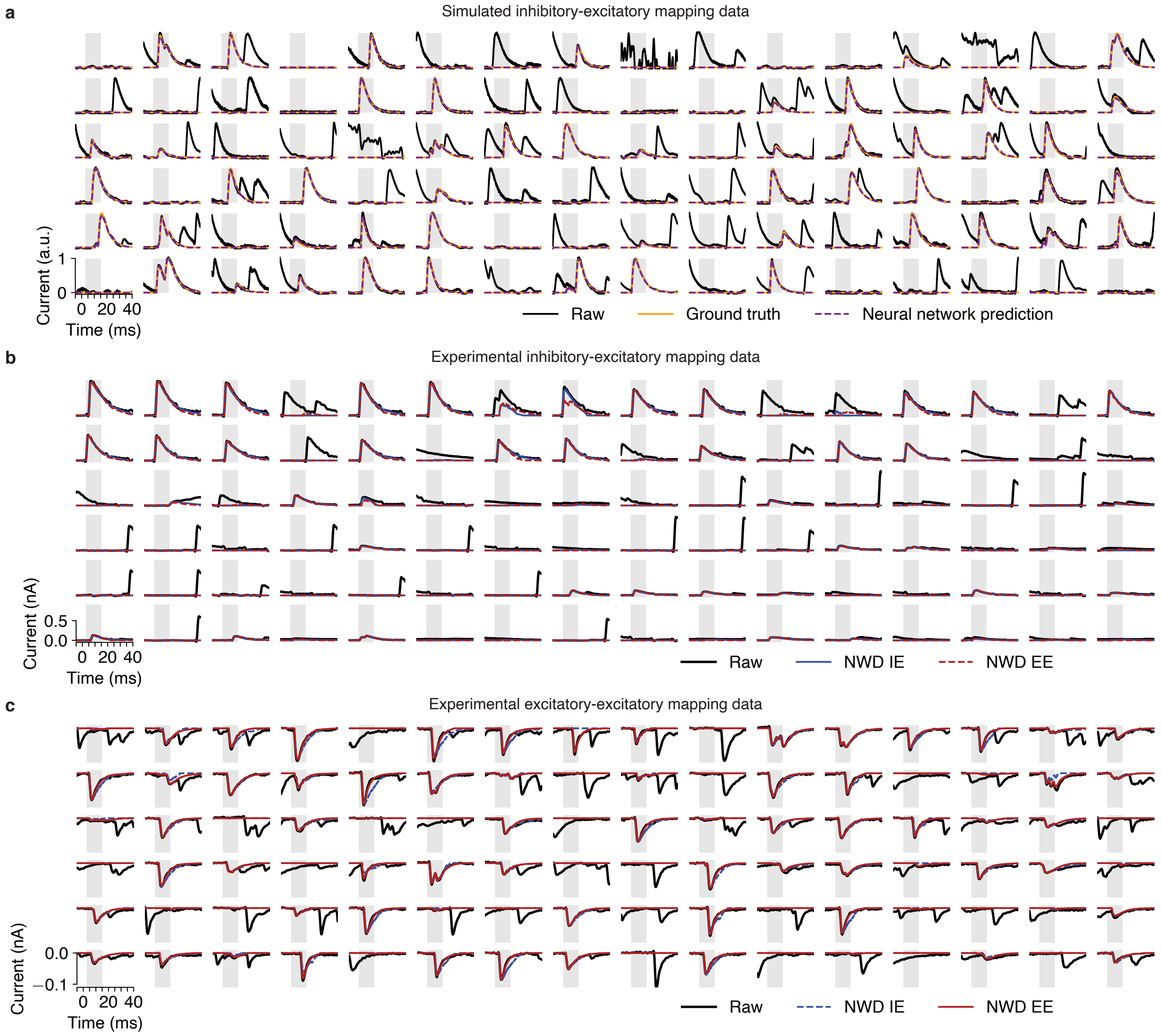

Supplementary Figure 1: **a**, NWD network performance on simulated test data. Solid black line shows raw data given as input to the neural network. Orange line shows target trace. Dashed purple line shows prediction from the NWD network. **b**, Performance of NWD trained on simulated data matched to IE mapping experiments (solid, blue). For comparison, NWD trained on simulated data matched to EE mapping experiments shown as dashed red line. **c**, Performance of NWD trained on simulated data matched to EE mapping experiments (solid, red). For comparison, NWD trained on simulated data matched to IE mapping experiments shown as dashed blue line.

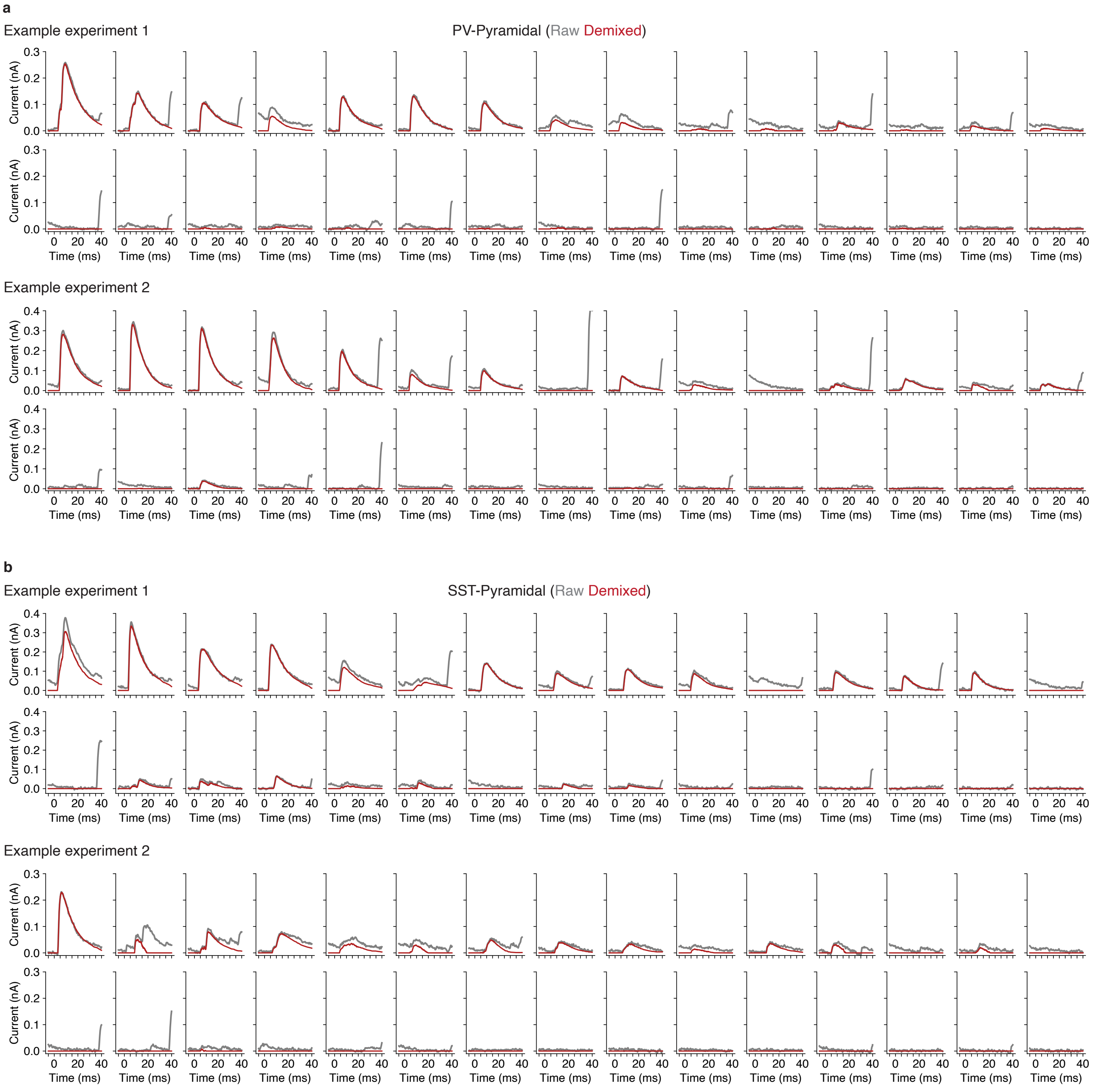

Supplementary Figure 2: Example application of NWD to individual PSCs evoked by holographic ensemble stimulation in PV (a) and SST (b) to pyramidal mapping experiments. PSCs selected uniformly at random and sorted by magnitude of postsynaptic response.

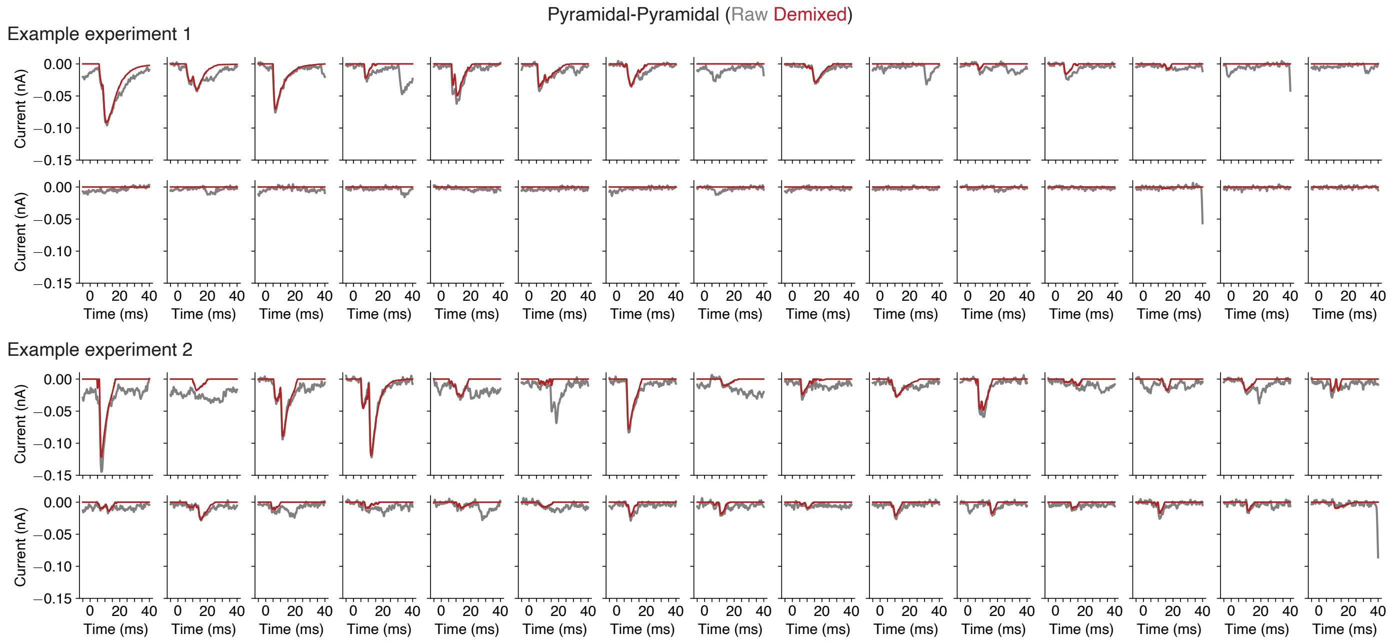

Supplementary Figure 3: Example application of NWD to individual PSCs evoked by holographic ensemble stimulation in a pyramidal-pyramidal mapping experiment. PSCs selected uniformly at random and sorted by magnitude of postsynaptic response.

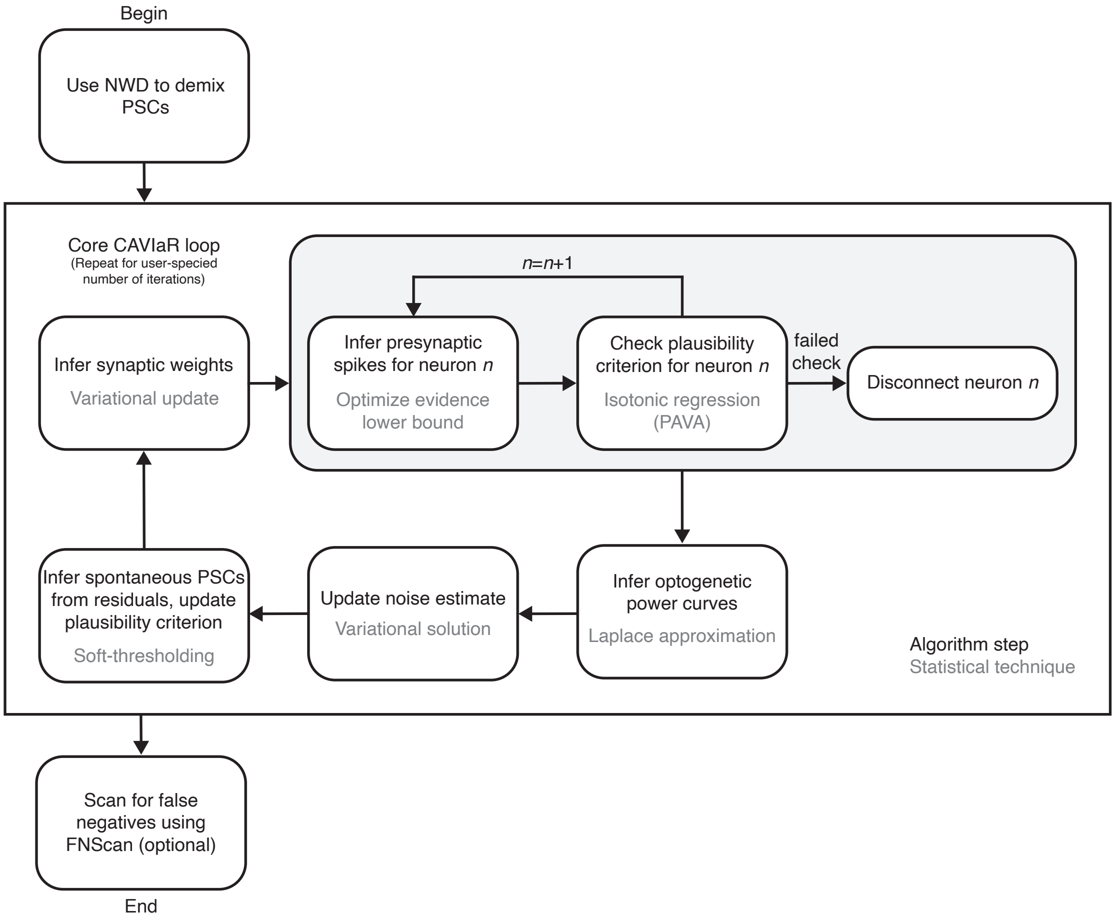

Supplementary Figure 4: Flowchart detailing the basic logic of the CAVIaR algorithm (cf. Algorithm 1).

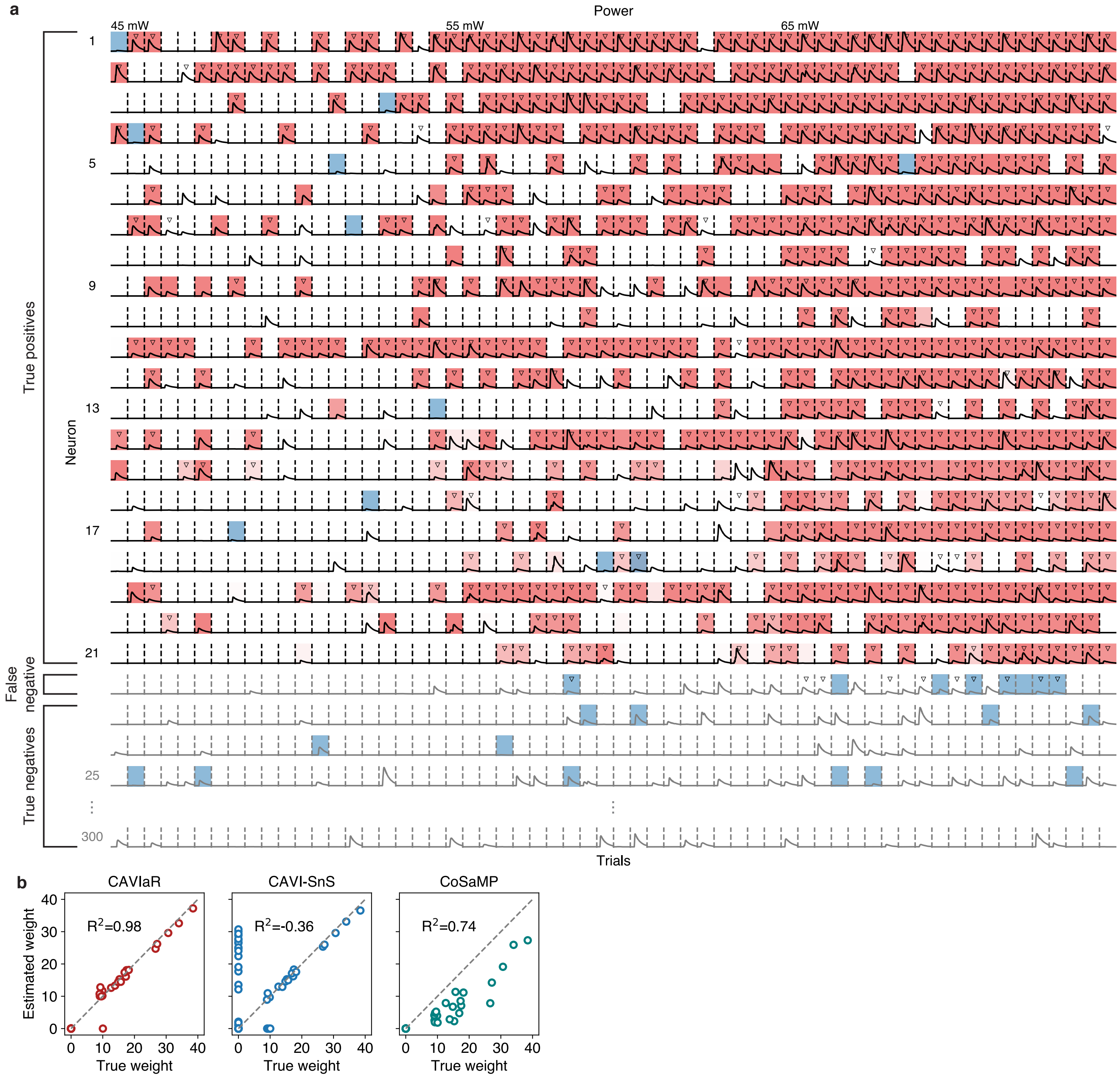

Supplementary Figure 5: Large-scale classification of presynaptic spikes, spontaneous synaptic currents, and synaptic connectivity in a 3 minute simulated connectivity mapping experiment. N =300 neurons mapped using 10-target ensemble stimulation at 10 Hz. Spontaneous PSCs occur at a rate of 5 Hz. **a**, "Checkerboard" visualization of CAVIaR model inference. Each row shows sample of PSCs evoked by stimulating the listed neuron across multiple powers. In this example, 9 neurons in addition to the listed neuron are stimulated on each trial. Shaded red cells indicate detected presynaptic spike for listed neuron. Shaded blue cells indicate detected spontaneous PSC. Triangles indicate ground-truth presynaptic spikes. Accuracy of inferred presynaptic spikes for this simulation, 94.3%. Traces shown in gray indicate neurons that the model declared disconnected. Out of 300 neurons mapped, one putative connection was a false negative (neuron 22) due to CAVIaR incorrectly assigning its PSCs to other stimulated neurons or to spontaneous activity. **b**, Comparison of connectivity inference accuracy between CAVIaR, CAVI-SnS, and CoSaMP.

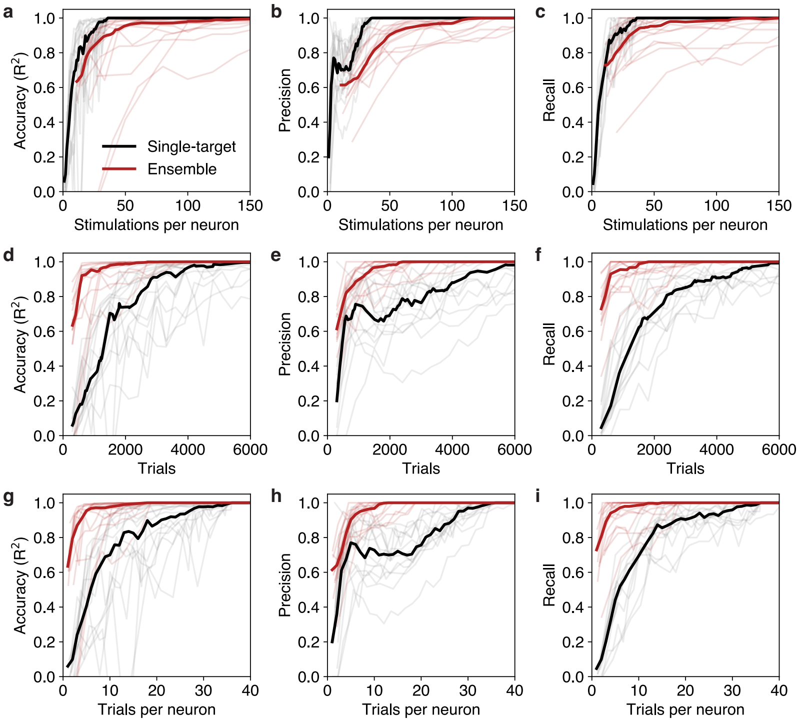

Supplementary Figure 6: Analysis of convergence speed using single-target stimulation (black lines) and compressed sensing (red lines). **a-c**, Convergence of accuracy metrics (R^2, precision, recall) as a function of number of times any given neuron is stimulated. **d-f**, Same as a-c, but for number of stimulation trials. **g-i**, same as d-f, but where trial counts have been normalized by population size (due to each experiment mapping different numbers of neurons). Dark lines show medians over 14 PV-pyramidal mapping experiments, faint lines show individual experiments. Note that performance metrics are defined with respect to the final estimates of each method, and therefore necessarily converge to 1. Hence "convergence" indicates "convergence to each method's final estimate of connectivity".

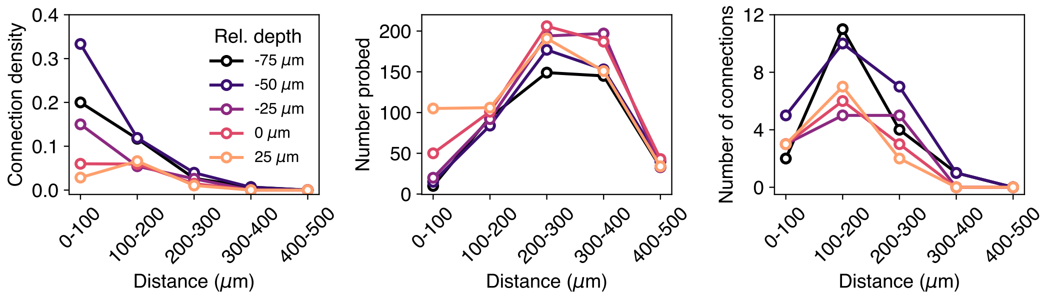

Supplementary Figure 7: Connection density, number of probed targets, and number of identified connections across 14 PV-pyramidal mapping experiments. Each line represents the result of probing for connectivity among PV neurons at a different depth relative to the postsynaptic pyramidal neuron. Distance from the postsynaptic neuron is evaluated in two dimensions (cf. Fig. 5f). Depth is with respect to the mediolateral dimension. Pyramidal neurons were patched in L2/3, but could be at different depths within L2/3 themselves.

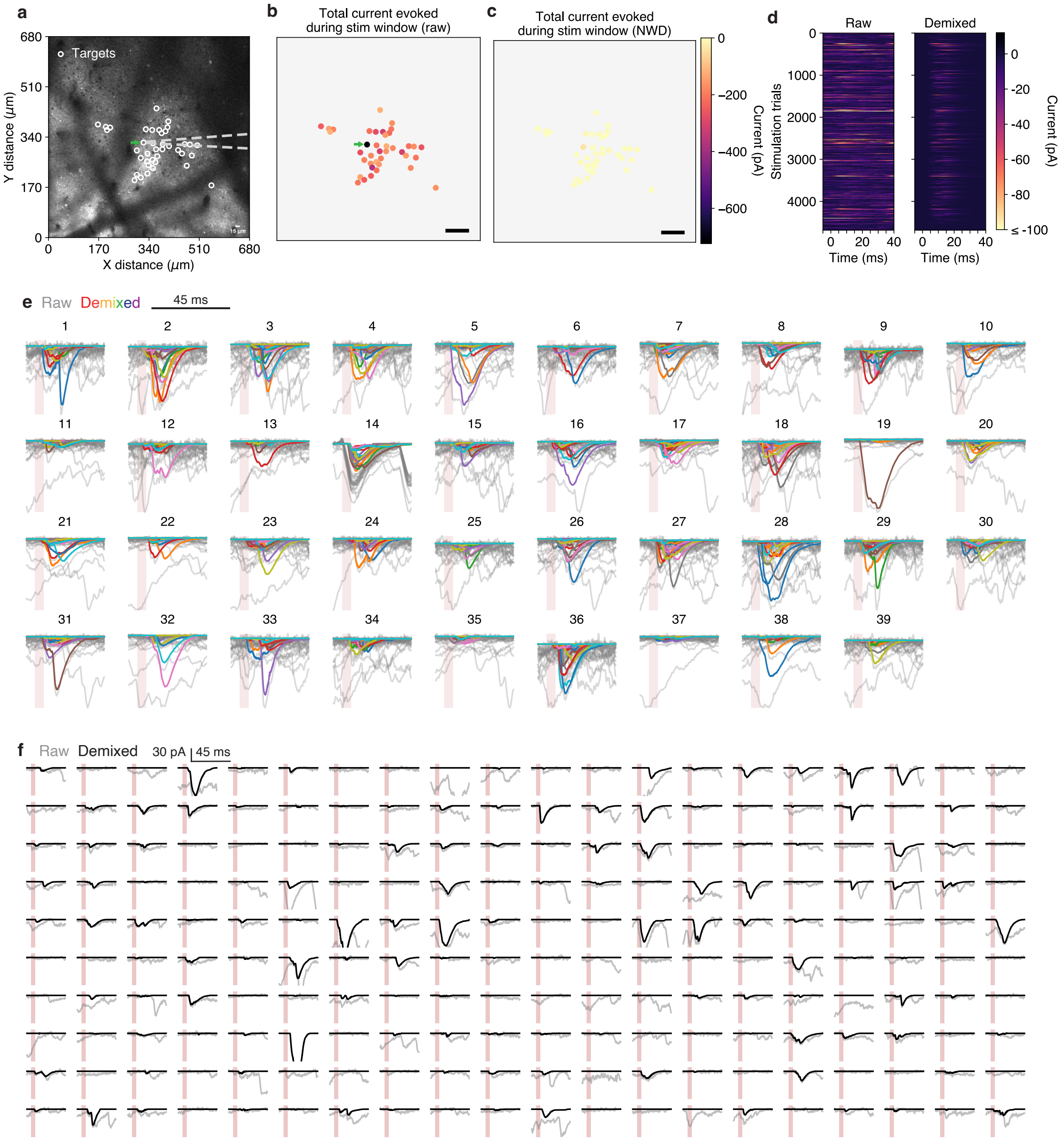

Supplementary Figure 8: Application of NWD to an *in vivo* connectivity mapping experiment. **a**, Field of view for an experiment mapping pyramidal-to-SST connections. Pyramidal neurons and some SST neurons (including the patched postsynaptic neuron) express a GCaMP8m-ChroME2s fusion (though no calcium imaging was performed). White circles represent stimulation targets. **b**, Map of the total current measured when stimulating at each location during the 5 ms stimulation window. Total current defined as the cumulative sum of the current (resulting from photostimulation or spontaneous input) over time. **c**, Same as b, but after applying NWD to suppress photocurrents. **d**, Matrix visualization of the postsynaptic current evoked by stimulation before (left) and after (right) demixing. **e**, Comparison of raw (gray traces) and demixed (colored traces) synaptic currents evoked by stimulation. Each cluster of traces corresponds to the stimulation of a different target (indicated by number above traces). Traces normalized between 0 and 1 for each target. Cf. raw vs demixed EPSCs in Extended Data Fig. 9 for an analogous *in vitro* experiment. **f**, Comparison of raw (gray) and demixed (black) traces for individual trials. Shaded red bars correspond to 5 ms stimulation periods. NWD successfully isolates plausible EPSCs under *in vivo* conditions.

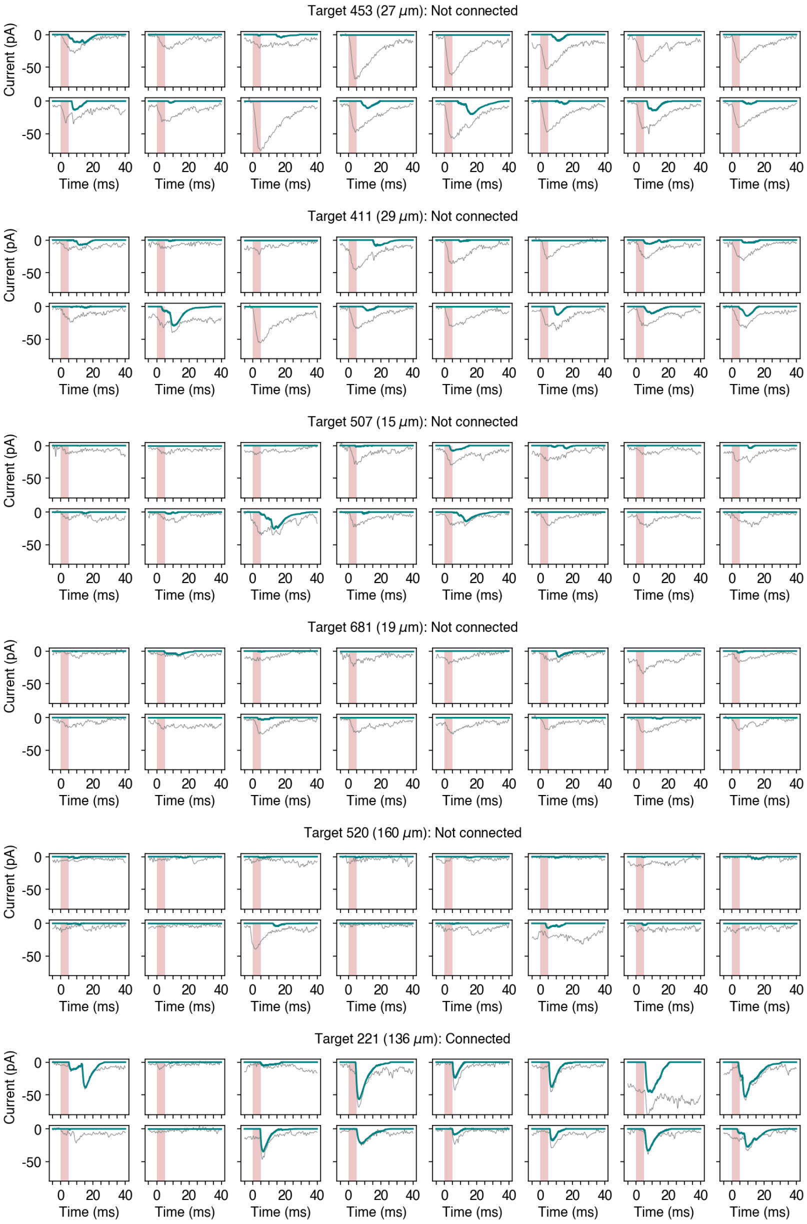

Supplementary Figure 9: Comparison between raw (gray) and demixed (teal) current traces in the presence of photocurrent artifacts. Traces correspond to all shown targets in Extended Data Fig. 9a (i.e. PSCs evoked by single-target stimulation, ordered by largest photocurrents).

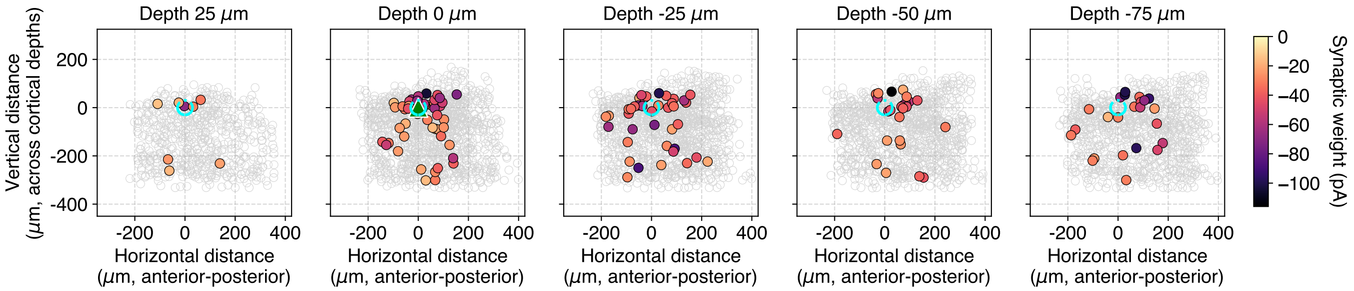

Supplementary Figure 10: Pyramidal-pyramidal connections across 10 experiments, split by plane. Cyan circle denotes region with 30 μm radius where photocurrents are most likely.

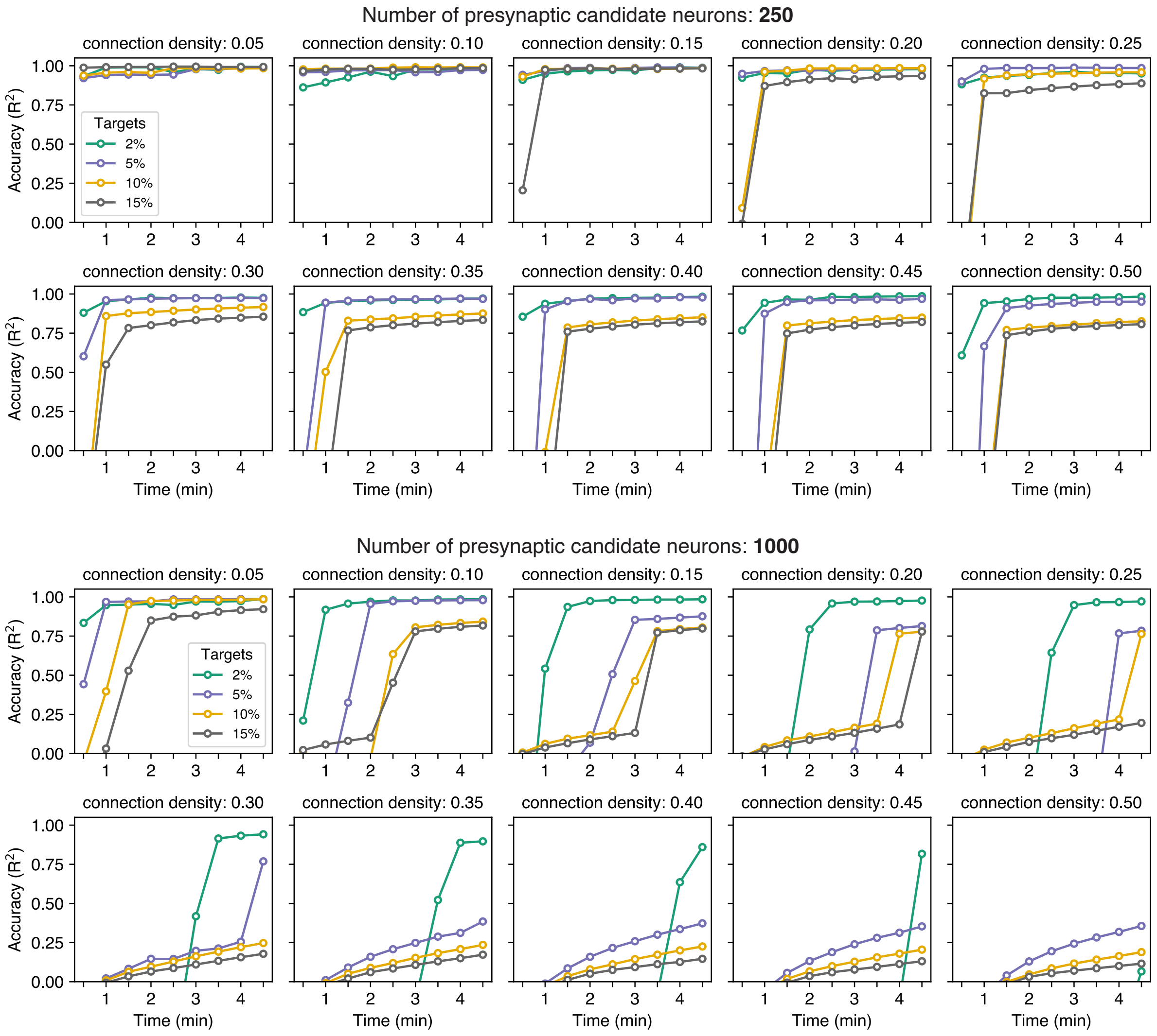

Supplementary Figure 11: The optimal number of stimulation targets depends on both connection density and population size. The ideal number of neurons to simultaneously stimulate depends on connection density. Simulation using CAVIaR with N=250 neurons probed (top two rows) or N=1000 (bottom two rows). Targets tested for N=250: 5, 13, 25, 38 (as percentages: 2%, 5%, 10%, 15% of total population stimulated per trial). Targets tested for N=1000: 20, 50, 100, 150. Spontaneous activity rate, 1 Hz. Stimulation frequency, 50 Hz. Each data point is an average of 10 simulations.

Supplementary Note 1

In this note, we consider the probability of encountering a spurious connection where spontaneous PSCs arrive only when stimulating an unconnected neuron at the maximum laser power, but not at any of the lower powers. In particular, we show that the probability of a spurious connection only appearing at the highest laser power is vanishingly low.

Assume we stimulate a putative neuron 10 times per power at three different powers, and that the probability of a spontaneous PSC arriving within the stimulus response window is p. The probability that at least 3/10 trials show a spontaneous PSC is given by summing the binomial probability

$$\mathbb{P}(\text{at least 3/10 PSCs}) = \sum_{k=3}^{10} \binom{10}{k} p^k (1-p)^{10-k}$$

Thus, the probability of at least 3/10 spontaneous PSCs at the highest power and none at the lower two powers is

$$\mathbb{P}(\text{at least 3/10 PSCs at max power only}) = \left(\sum_{k=3}^{10} \binom{10}{k} p^k (1-p)^{10-k} \right) \cdot (1-p)^{10} \cdot (1-p)^{10}.$$

Plotting the above expression as a function of spontaneous PSC probability p yields Supplementary Fig. 12.

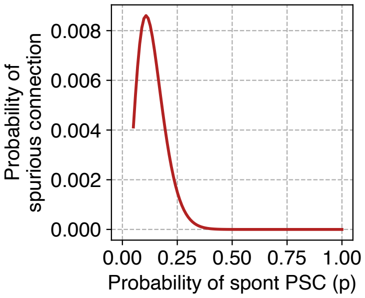

Supplementary Figure 12: Probability of a spurious connection arising due to spontaneous PSCs at maximum power only.

From Supplementary Fig. 12 one can see that as the rate of spontaneous activity increases, it becomes increasingly unlikely for spontaneous PSCs to arrive only at the maximum power. The maximum value of the probability curve is just 0.86% (attained when the probability of a spontaneous PSC coinciding with a stimulation trial is 0.1). Hence this scenario is so rare as to not be worth consideration in practice.

Supplementary Note 2

In this note, we consider whether intrinsic plasticity could have occurred as a result of repeated ensemble stimulation. Note that, as we argue in the discussion, the typical rate of stimulation for any given neuron is low – less than 3 Hz in our experiments, which is too slow to induce intrinsic plasticity for neurons expressing the ChroME2 opsins [2]. Nevertheless, we sought to confirm that no such intrinsic plasticity effects were present in our data.

To precisely determine variables related to intrinsic plasticity (e.g. the spiking threshold and the current-to-spike relationship) in our experiments, one would need to know exactly when neurons are spiking in response to ensemble stimulation. Technically, this would require challenging experiments based on voltage imaging, which are beyond the scope of our study. Fortunately, CAVIaR performs inference of when neurons spike in response to stimulation, thereby providing a useful approximation.

Since we cannot directly access the input currents and action potentials for the neurons we map, we have approximated the spiking threshold and the current-to-spike relationship in the following way. First, the spike threshold was approximated as the minimal laser power required to spike a neuron with at least 0.25 probability. Second, the current-to-spike relationship was approximated by the “power-to-spike relationship” (i.e., spike probability as a function of laser power). In both cases, the spikes were inferred using CAVIaR.

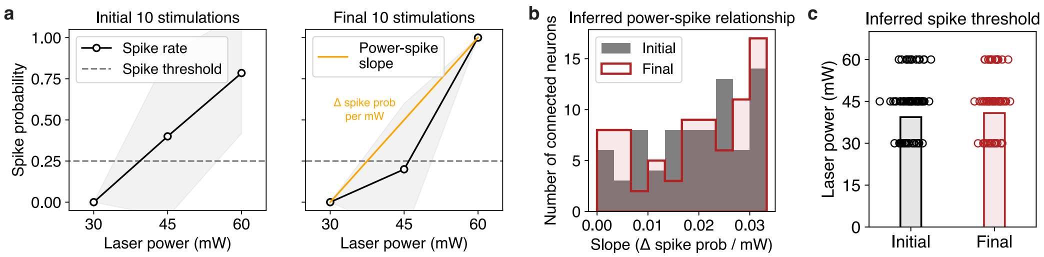

Supplementary Figure 13: Analysis of potential changes in spike threshold or power-dependence as a result of repeated ensemble stimulation. a, Probability of spiking as a function of laser power using the initial 10 spikes (left) vs the final 10 spikes (right). Shaded error bars represent one standard deviation. Dashed horizontal line represents a spike probability of 0.25. The least power required to cross a spike probability of 0.25 is considered the "spike threshold" laser power. Orange line represents the "power-spike" slope; i.e. the change in spike probability per mW laser power. The power-spike slope is intended to approximate the slope of the current-to-spike relationship. **b**, No statistically significant difference between spike threshold before (initial) vs after (final) repeated ensemble stimulation (p=0.16, Wilcoxon signed-rank test). Shaded bars show means across all neurons and all experiments. **c**, No statistically significant difference between power-spike slope before vs after ensemble stimulation (p=0.57, Wilcoxon signed-rank test). All spikes are inferred from PSCs using CAVIaR. Analysis performed on all 78 detected presynaptic neurons across all PV-pyramidal experiments.

Next, to determine whether such variables changed with repeated stimulation, we estimated the power-spike relationship using both the initial 10 ensemble stimulation trials and the final 10 ensemble stimulation trials (Supplementary Fig. 13a) for every neuron across every experiment. This showed firstly that spike threshold did not significantly change over the course of the experiments (p=0.16, Wilcoxon signed-rank test; Supplementary Fig. 13b), and secondly that the slope of the power-spike relationship (i.e. the change in spike probability per mW laser power) also did not significantly change (p=0.57, Wilcoxon signed-rank test; Supplementary Fig. 13c). We repeated the analysis based on responses to single-target stimulation and found that there was a negligible but statistically significant change in the slope of the power-spike relationship (average difference in slope, 6×10^{-4}; p=0.02, Wilcoxon signed-rank test), and no difference in spike threshold (p=0.74). However, we note that these metrics rely on CAVIaR's inferences (which are not necessarily perfect), and that with perfect knowledge of how each neuron spiked these results could change.

We also verified that the input resistance of the postsynaptic neuron at the beginning and end of the experiment were not significantly different (mean input resistance at beginning of experiments, 105 MΩ; mean input resistance at end of experiments 109 MΩ; p-value of difference=0.85, independent t-test).

Supplementary Note 3

In this note we address the fact that in some experimental preparations, opsin-expressing neurons do not also express a separate nuclear fluorophore for straightforward identification of photosensitive cell nuclei [24]. We therefore considered whether our connectivity mapping system could be used in the case where neuron locations are not known *a priori* (i.e., when mapping is performed "blind"). In this context, the field of view is divided into a three-dimensional grid and locations are stimulated one grid point at a time [4, 25, 26].

We performed both single-target and 10-target stimulation of grid points at 30 Hz and applied NWD and CAVIaR independently to the two sets of mapping data. Both single-target and ensemble stimulation identified a common ROI, and overlaying the stimulation grid on an image of the underlying tissue showed that this ROI aligned with a unique opsin-expressing neuron (Supplementary Fig. 14; see Supplementary Fig. 15 for full, multi-plane analysis of blind grid mapping experiment). This confirmed that NWD and CAVIaR could also facilitate rapid connectivity mapping in the blind stimulation regime.

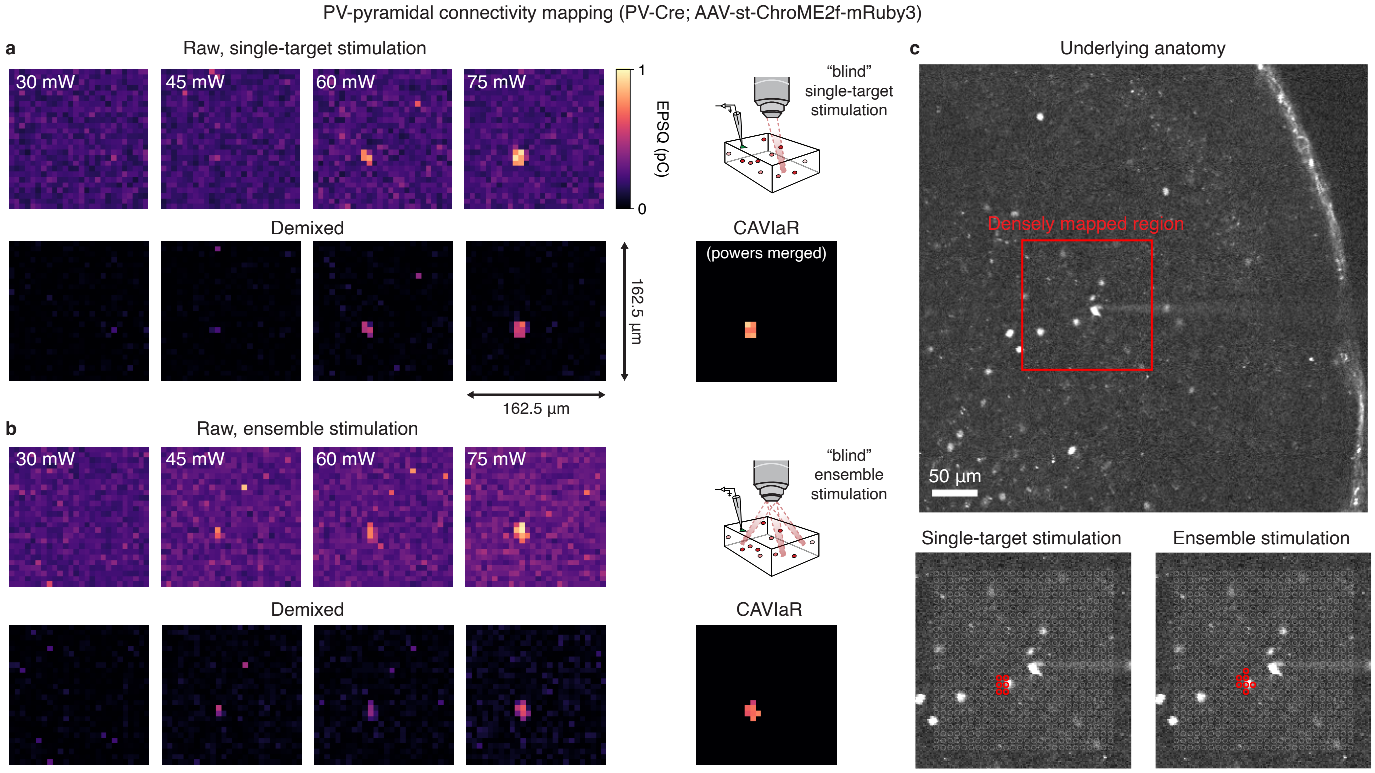

Supplementary Figure 14: Demixing and connectivity inference for "blind" grid mapping experiments. **a**, Grid mapping of PV-pyramidal connectivity using single-target stimulation over four powers (30-75 mW). One out of five planes are shown as an example. Note that maps of synaptic connectivity obtained by CAVIaR "merge" the multi-power demixed maps into a single map. **b**, Grid mapping of same PV-pyramidal experiment using holographic ensemble stimulation (on five planes over 25 to -75 μm, separated by 25 μm each; only plane 0 μm shown for comparison) over four powers. Agreement between the two maps validates the use of CAVIaR in this regime. Each pixel in the raw maps shown in (a) and (b) is obtained by averaging across all PSCs evoked by stimulation of an ensemble containing that pixel. **c**, Overlay of connected pixels (red circles) on underlying anatomy (obtained by imaging expression of mRuby) confirms that grid mapping using both single-target and ensemble stimulation correctly identifies an opsin-expressing neuron. Also note that this example experiment includes laser powers up to 75 mW (exceeding the range used to characterize PPSFs in Extended Data Fig. 1), which should be considered when interpreting the sizes of the connected regions.

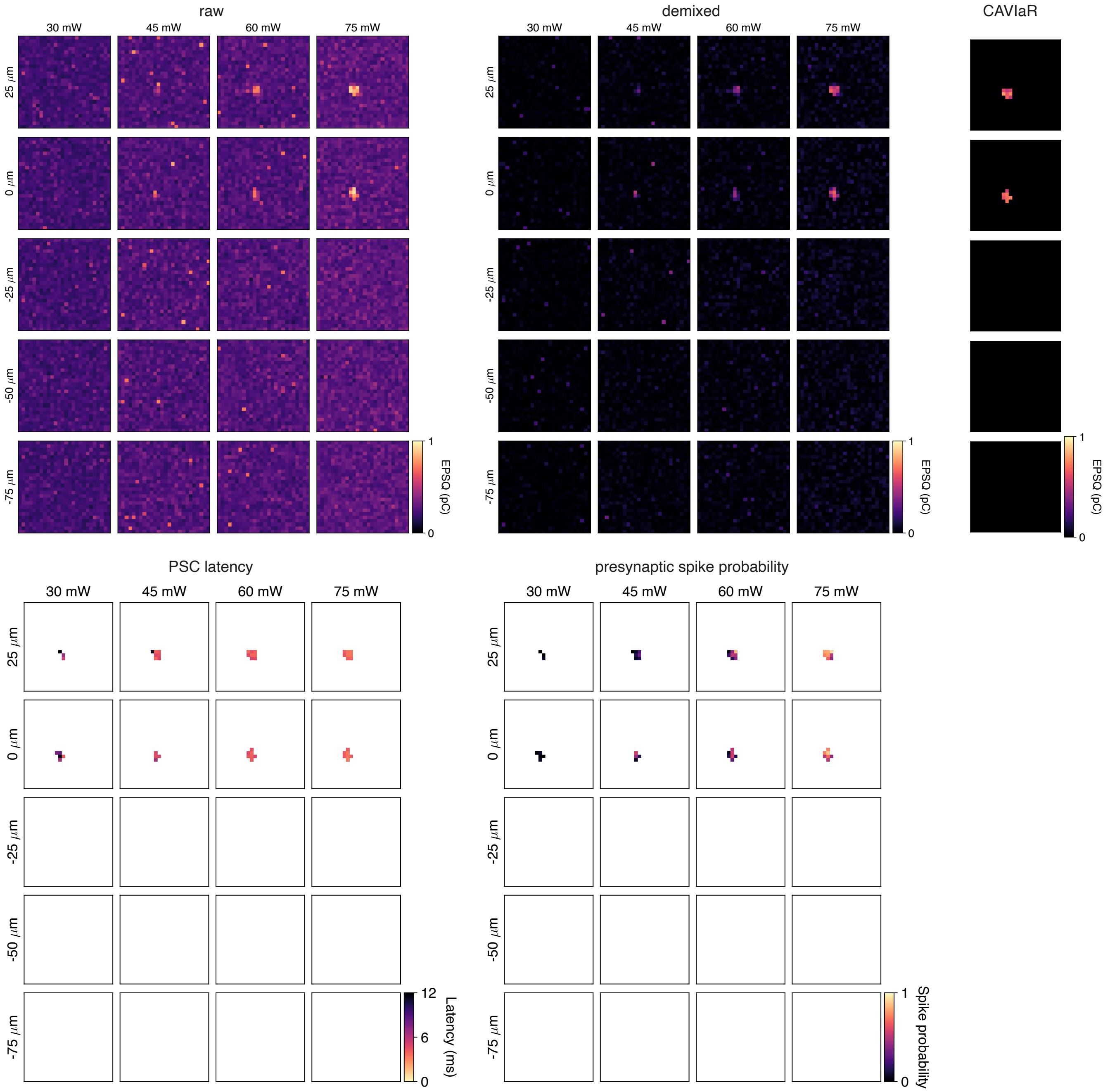

Supplementary Figure 15: Demixing and denoising of optogenetic "blind" grid mapping data using NWD and CAVIaR. Example shows PV-pyramidal connectivity mapping (PV-Cre ChroME2f). Postsynaptic cell is on plane 0 μm.

References

[1] James H Marshel, Yoon Seok Kim, Timothy A Machado, Sean Quirin, Brandon Benson, Jonathan Kadmon, Cephra Raja, Adelaida Chibukhchyan, Charu Ramakrishnan, Masatoshi Inoue, et al. Cortical layer–specific critical dynamics triggering perception. *Science*, 365(6453):eaaw5202, 2019.

[2] Savitha Sridharan, Marta A Gajowa, Mora B Ogando, Uday K Jagadisan, Lamiae Abdeladim, Masato Sadahiro, Hayley A Bounds, William D Hendricks, Toby S Turney, Ian Tayler, et al. High-performance microbial opsins for spatially and temporally precise perturbations of large neuronal networks. *Neuron*, 2022.

[3] Alan R Mardinly, Ian Antón Oldenburg, Nicolas C Pégard, Savitha Sridharan, Evan H Lyall, Kirill Chesnov, Stephen G Brohawn, Laura Waller, and Hillel Adesnik. Precise multimodal optical control of neural ensemble activity. *Nature neuroscience*, 21(6):881–893, 2018.

[4] Christopher A Baker, Yishai M Elyada, Andres Parra, and M McLean Bolton. Cellular resolution circuit mapping with temporal-focused excitation of soma-targeted channelrhodopsin. *Elife*, 5:e14193, 2016.

[5] Or A Shemesh, Dimitrii Tanese, Valeria Zampini, Changyang Linghu, Kiryl Piatkevich, Emiliano Ronzitti, Eirini Papagiakoumou, Edward S Boyden, and Valentina Emiliani. Temporally precise single-cell-resolution optogenetics. *Nature neuroscience*, 20(12):1796–1806, 2017.

[6] Eirini Papagiakoumou, Francesca Anselmi, Aurélien Bègue, Vincent De Sars, Jesper Glückstad, Ehud Y Isacoff, and Valentina Emiliani. Scanless two-photon excitation of channelrhodopsin-2. *Nature methods*, 7(10):848–854, 2010.

[7] Oscar Hernandez, Eirini Papagiakoumou, Dimitrii Tanese, Kevin Fidelin, Claire Wyart, and Valentina Emiliani. Three-dimensional spatiotemporal focusing of holographic patterns. *Nature communications*, 7(1):1–11, 2016.

[8] Nicolas C Pégard, Alan R Mardinly, Ian Antón Oldenburg, Savitha Sridharan, Laura Waller, and Hillel Adesnik. Three-dimensional scanless holographic optogenetics with temporal focusing (3d-shot). *Nature communications*, 8(1):1–14, 2017.

[9] Hillel Adesnik and Lamiae Abdeladim. Probing neural codes with two-photon holographic optogenetics. *Nature Neuroscience*, 24(10):1356–1366, 2021.

[10] Marcus Triplett, Marta Gajowa, Hillel Adesnik, and Liam Paninski. Bayesian target optimisation for high-precision holographic optogenetics. *Advances in Neural Information Processing Systems*, 36, 2024.

[11] Phillip Navarro and Karim Oweiss. Compressive sensing of functional connectivity maps from patterned optogenetic stimulation of neuronal ensembles. *Patterns*, 2023.

[12] Rodrigo Perin, Thomas K Berger, and Henry Markram. A synaptic organizing principle for cortical neuronal groups. *Proceedings of the National Academy of Sciences*, 108(13):5419–5424, 2011.

[13] M Hossein Eybposh, Nicholas W Caira, Mathew Atisa, Praneeth Chakravarthula, and Nicolas C Pégard. Deepcgh: 3d computer-generated holography using deep learning. *Optics Express*, 28(18):26636–26650, 2020.

[14] M Hossein Eybposh, Vincent R Curtis, Jose Rodríguez-Romaguera, and Nicolas C Pégard. Advances in computer-generated holography for targeted neuronal modulation. *Neurophotonics*, 9(4):041409, 2022.

[15] Ami Citri and Robert C Malenka. Synaptic plasticity: multiple forms, functions, and mechanisms. *Neuropsychopharmacology*, 33(1):18–41, 2008.

[16] Ian Antón Oldenburg, William D Hendricks, Gregory Handy, Kiarash Shamardani, Hayley A Bounds, Brent Doiron, and Hillel Adesnik. The logic of recurrent circuits in the primary visual cortex. *bioRxiv*, pages 2022–09, 2022.

[17] Adam M Packer, Darcy S Peterka, Jan J Hirtz, Rohit Prakash, Karl Deisseroth, and Rafael Yuste. Two-photon optogenetics of dendritic spines and neural circuits. *Nature methods*, 9(12):1202–1205, 2012.

[18] Travis A Hage, Alice Bosma-Moody, Christopher A Baker, Megan B Kratz, Luke Campagnola, Tim Jarsky, Hongkui Zeng, and Gabe J Murphy. Synaptic connectivity to l2/3 of primary visual cortex measured by two-photon optogenetic stimulation. *Elife*, 11:e71103, 2022.

[19] Benjamin Antin, Masato Sadahiro, Marta Gajowa, Marcus A Triplett, Hillel Adesnik, and Liam Paninski. Removing direct photocurrent artifacts in optogenetic connectivity mapping data via constrained matrix factorization. *PLOS Computational Biology*, 20(5):e1012053, 2024.

[20] Tao Hu and Dmitri Chklovskii. Reconstruction of sparse circuits using multi-neuronal excitation (rescume). *Advances in Neural Information Processing Systems*, 22:790–798, 2009.

[21] Stephen R Williams and Simon J Mitchell. Direct measurement of somatic voltage clamp errors in central neurons. *Nature neuroscience*, 11(7):790–798, 2008.

[22] Attila Losonczy and Jeffrey C Magee. Integrative properties of radial oblique dendrites in hippocampal ca1 pyramidal neurons. *Neuron*, 50(2):291–307, 2006.

[23] Alexandra Tran-Van-Minh, Romain D Cazé, Therése Abrahamsson, Laurence Cathala, Boris S Gutkin, and David A DiGregorio. Contribution of sublinear and supralinear dendritic integration to neuronal computations. *Frontiers in cellular neuroscience*, 9:67, 2015.

[24] Hayley A Bounds, Masato Sadahiro, William D Hendricks, Marta Gajowa, Ian Antón Oldenburg, Karthika Gopakumar, Daniel Quintana, Tanya Daigle, Hongkui Zeng, and Hillel Adesnik. Multifunctional cre-dependent transgenic mice for high-precision all-optical interrogation of neural circuits. *bioRxiv*, 2021.

[25] H Wang, J Peca, M Matsuzaki, K Matsuzaki, J Noguchi, L Qiu, D Wang, F Zhang, E Boyden, K Deisseroth, et al. High-speed mapping of synaptic connectivity using photostimulation in channelrhodopsin-2 transgenic mice. *Proceedings of the National Academy of Sciences*, 104(19):8143–8148, 2007.

[26] Alexander Naka, Julia Veit, Ben Shababo, Rebecca K Chance, Davide Risso, David Stafford, Benjamin Snyder, Andrew Egladyous, Desiree Chu, Savitha Sridharan, et al. Complementary networks of cortical somatostatin interneurons enforce layer specific control. *Elife*, 8:e43696, 2019.
