# Supplementary material for: Rapid learning of neural circuitry from holographic ensemble stimulation enabled by model-based compressed sensing
Source: Nat Neurosci. 2025 Sep 17;28(10):2154–65. doi: 10.1038/s41593-025-02053-7 (PMC12497653; doi:10.1038/s41593-025-02053-7)
Supplement: Supplementary file 2 — Reporting Summary [file 41593_2025_2053_MOESM2_ESM.pdf]

nature portfolio

Corresponding author(s): Marcus Triplett, Hillel Adesnik

Last updated by author(s): Jun 13, 2025

Reporting Summary

Nature Portfolio wishes to improve the reproducibility of the work that we publish. This form provides structure for consistency and transparency in reporting. For further information on Nature Portfolio policies, see our Editorial Policies and the Editorial Policy Checklist.

Statistics

For all statistical analyses, confirm that the following items are present in the figure legend, table legend, main text, or Methods section.

n/a	Confirmed	
☐	☒	The exact sample size (*n*) for each experimental group/condition, given as a discrete number and unit of measurement
☐	☒	A statement on whether measurements were taken from distinct samples or whether the same sample was measured repeatedly
☐	☒	The statistical test(s) used AND whether they are one- or two-sided<br>*Only common tests should be described solely by name; describe more complex techniques in the Methods section.*
☐	☒	A description of all covariates tested
☐	☒	A description of any assumptions or corrections, such as tests of normality and adjustment for multiple comparisons
☐	☒	A full description of the statistical parameters including central tendency (e.g. means) or other basic estimates (e.g. regression coefficient) AND variation (e.g. standard deviation) or associated estimates of uncertainty (e.g. confidence intervals)
☐	☒	For null hypothesis testing, the test statistic (e.g. *F*, *t*, *r*) with confidence intervals, effect sizes, degrees of freedom and *P* value noted<br>*Give P values as exact values whenever suitable.*
☐	☒	For Bayesian analysis, information on the choice of priors and Markov chain Monte Carlo settings
☐	☒	For hierarchical and complex designs, identification of the appropriate level for tests and full reporting of outcomes
☐	☒	Estimates of effect sizes (e.g. Cohen's *d*, Pearson's *r*), indicating how they were calculated

Our web collection on statistics for biologists contains articles on many of the points above.

Software and code

Policy information about availability of computer code

Data collection	MATLAB 2019, ScanImage 2018
Data analysis	MATLAB 2019, Python >= 3.8, NumPy >=1.21, SciPy >= 1.7.2, JAX, scikit-learn, Torch, PyTorch Lightning 1.9, tqdm, Pandas

For manuscripts utilizing custom algorithms or software that are central to the research but not yet described in published literature, software must be made available to editors and reviewers. We strongly encourage code deposition in a community repository (e.g. GitHub). See the Nature Portfolio guidelines for submitting code & software for further information.

Data

Policy information about availability of data

All manuscripts must include a data availability statement. This statement should provide the following information, where applicable:

- Accession codes, unique identifiers, or web links for publicly available datasets
- A description of any restrictions on data availability
- For clinical datasets or third party data, please ensure that the statement adheres to our policy

The experimental data for this manuscript are available via figshare at https://figshare.com/s/1315937c4ce1c2552262.

Research involving human participants, their data, or biological material

Policy information about studies with human participants or human data. See also policy information about sex, gender (identity/presentation), and sexual orientation and race, ethnicity and racism.

Reporting on sex and gender	N/A
Reporting on race, ethnicity, or other socially relevant groupings	N/A
Population characteristics	N/A
Recruitment	N/A
Ethics oversight	N/A

Note that full information on the approval of the study protocol must also be provided in the manuscript.

Field-specific reporting

Please select the one below that is the best fit for your research. If you are not sure, read the appropriate sections before making your selection.

☒ Life sciences ☐ Behavioural & social sciences ☐ Ecological, evolutionary & environmental sciences

For a reference copy of the document with all sections, see nature.com/documents/nr-reporting-summary-flat.pdf

Life sciences study design

All studies must disclose on these points even when the disclosure is negative.

Sample size	The main validation of the manuscript features synaptic connectivity maps obtained from the same designated postsynaptic neurons using two approaches: one based on single-target stimulation and one based on ensemble stimulation (i.e., compressed sensing). In both cases, the same pool of targets in the local FOV was probed. Therefore, the sample size (number of probed presynaptic candidate neurons) was equal to the number of opsin-expressing neurons in the FOV (ranging from approximately 100 to 700).
Data exclusions	Trials were excluded if patch access resistance changed by more than 35% relative to the initial value (averaged from first 5 measurements). Experiments were excluded if less than 20% of experimental trials remained, as this did not provide sufficient data to estimate connectivity maps. Finally, experiments finding 0 or 1 synaptic connection(s) were excluded as they were likely caused by severed connections and/or insufficient opsin expression.
Replication	The computational methods (NWD, CAVIaR) were first validated using simulations. Then, we applied these methods to mapping data collected using single-target and ensemble stimulation to determine the extent to which they identify the same connections. We then replicated this process across a variety of cell-type combinations: pyramidal-pyramidal (n=10), PV-pyramidal (n=14), pyramidal-PV (n=6), and SST-pyramidal (n=9). All data, besides those excluded as described above, are featured in the manuscript.
Randomization	All experiments randomize between sweeps of single-target and ensemble stimulation, across laser powers, and the order in which presynaptic candidate neurons are probed.
Blinding	Each presynaptic candidate neuron was probed on multiple trials using both single-target and ensemble stimulation during the same, randomized experiment, and therefore blinding was not applicable. The knowledge of pre- and postsynaptic cell types was used to apply the appropriate NWD network, and therefore this information could not be blinded during analysis.

Reporting for specific materials, systems and methods

We require information from authors about some types of materials, experimental systems and methods used in many studies. Here, indicate whether each material, system or method listed is relevant to your study. If you are not sure if a list item applies to your research, read the appropriate section before selecting a response.

Materials & experimental systems

n/a	Involved in the study
☒	☐ Antibodies
☒	☐ Eukaryotic cell lines
☒	☐ Palaeontology and archaeology
☐	☒ Animals and other organisms
☒	☐ Clinical data
☒	☐ Dual use research of concern
☒	☐ Plants

Methods

n/a	Involved in the study
☒	☐ ChIP-seq
☒	☐ Flow cytometry
☒	☐ MRI-based neuroimaging

Animals and other research organisms

Policy information about studies involving animals; ARRIVE guidelines recommended for reporting animal research, and Sex and Gender in Research

Laboratory animals	Mice used for experiments in this study were transgenic Emx-Cre, PV-Cre, or SST-Cre mice obtained by crossing the corresponding lines in-house with a wild type (CD-1 (ICR) white strain, obtained from Jackson Labs). Mice were housed in cohorts of five or fewer in a reverse light:dark cycle of 12:12 hours, with experiments occurring during the dark phase. In vitro slice recordings were performed on 300 µm-thick coronal slices coming from 4–6-week-old animals expressing opsin in V1.
Wild animals	N/A
Reporting on sex	In all experiments we attempted to use male and female mice equally.
Field-collected samples	N/A
Ethics oversight	All experiments on animals were conducted with approval of the Animal Care and Use Committee of the University of California, Berkeley.

Note that full information on the approval of the study protocol must also be provided in the manuscript.

Plants

Seed stocks	*Report on the source of all seed stocks or other plant material used. If applicable, state the seed stock centre and catalogue number. If plant specimens were collected from the field, describe the collection location, date and sampling procedures.*
Novel plant genotypes	*Describe the methods by which all novel plant genotypes were produced. This includes those generated by transgenic approaches, gene editing, chemical/radiation-based mutagenesis and hybridization. For transgenic lines, describe the transformation method, the number of independent lines analyzed and the generation upon which experiments were performed. For gene-edited lines, describe the editor used, the endogenous sequence targeted for editing, the targeting guide RNA sequence (if applicable) and how the editor was applied.*
Authentication	*Describe any authentication procedures for each seed stock used or novel genotype generated. Describe any experiments used to assess the effect of a mutation and, where applicable, how potential secondary effects (e.g. second site T-DNA insertions, mosiacism, off-target gene editing) were examined.*
